# Supplementary material for: A workflow reproducibility scale for automatic validation of biological interpretation results
Source: Gigascience. 2023 May 8;12:giad031. doi: 10.1093/gigascience/giad031 (PMC10164546; doi:10.1093/gigascience/giad031)
Supplement: giad031_GIGA-D-22-00300_Revision_1 [file giad031_giga-d-22-00300_revision_1.pdf]

GigaScience

A workflow reproducibility scale for automatic validation of biological interpretation results

--Manuscript Draft--

**Manuscript Number:**	GIGA-D-22-00300R1
**Full Title:**	A workflow reproducibility scale for automatic validation of biological interpretation results
**Article Type:**	Research
**Funding Information:**	Japan Society for the Promotion of Science London (20J22439) — Mr. Hirotaka Suetake<br>Core Research for Evolutional Science and Technology (JPMJCR17A1) — Dr. Takeo Igarashi<br>National Bioscience Database Center — Dr. Tazro Ohta
**Abstract:**	Background<br>Reproducibility of data analysis workflow is a key issue in the field of bioinformatics. Recent computing technologies, such as virtualization, have made it possible to reproduce workflow execution with ease. However, the reproducibility of results is not well discussed; that is, there is no standard way to verify whether the biological interpretation of reproduced results are the same. Therefore, it still remains a challenge to automatically evaluate the reproducibility of results.<br>Results<br>We propose a new metric, a reproducibility scale of workflow execution results, to evaluate the reproducibility of results. This metric is based on the idea of evaluating the reproducibility of results using biological feature values (e.g., number of reads, mapping rate, and variant frequency) representing their biological interpretation. We also implemented a prototype system that automatically evaluates the reproducibility of results using the proposed metric. To demonstrate our approach, we conducted an experiment using workflows used by researchers in real research projects and the use cases that are frequently encountered in the field of bioinformatics.<br>Conclusions<br>Our approach enables automatic evaluation of the reproducibility of results using a fine-grained scale. By introducing our approach, it is possible to evolve from a binary view of whether the results are superficially identical or not to a more graduated view. We believe that our approach will contribute to more informed discussion on reproducibility in bioinformatics.
**Corresponding Author:**	Tazro Ohta<br><br>JAPAN
**Corresponding Author Secondary Information:**	
**Corresponding Author's Institution:**	
**Corresponding Author's Secondary Institution:**	
**First Author:**	Hirotaka Suetake
**First Author Secondary Information:**	
**Order of Authors:**	Hirotaka Suetake<br>Tsukasa Fukusato<br>Takeo Igarashi<br>Tazro Ohta
**Order of Authors Secondary Information:**	

Powered by Editorial Manager® and ProduXion Manager® from Aries Systems Corporation

Response to Reviewers:	GIGA-D-22-00300<br>A workflow reproducibility scale for automatic validation of biological interpretation results<br>Hirotaka Suetake; Tsukasa Fukusato; Takeo Igarashi; Tazro Ohta<br>GigaScience<br><br>We would like to thank the reviewers for their positive and constructive feedback. The revised manuscript highlighted the changes in red color. Our responses to the issues pointed out by each reviewer are as follows.<br><br>## Reviewer #1<br><br>> Make explicit why these 3 workflows where selected (see Q2)<br><br>> (From Q2:) It is not explicit in the text why these particular workflows were selected, beyond being realistic pipelines used in research. I would suggest something like "these workflows have been selected as fairly representative and mature current best-practice for sequencing pipelines, implemented in different but typical workflow systems, and have similar set of genomics features that we can assess for provenance comparison.<br><br>We are appreciated for the suggestion. We added the suggested sentence in the first paragraph of the Result section.<br><br>> Make pipeline software citations consistent in manuscript (see Q2, Q5)<br><br>We fixed the citation with the appropriate publications and the URL for the software.<br><br>> Avoid declaring CC0 within generated RO-Crate -- move this to only apply to the ro-crate-metadata.json<br><br>We fixed the indicated license issue in our Zenodo repository by updating the contents. We also updated the DOI in the manuscript. We added the following paragraph to the Discussion section to explain the license issue related to an auto-generated RO-crate:<br><br>*When generating workflow provenance using a format, such as RO-Crate, it is important to consider licensing issues. The provenance includes not only the execution results, but also the executed workflow, input datasets, and software used internally. These files and software may have different licenses, and combining them under a single license can cause relicensing problems. In RO-Crate, a license can be specified for each entity; however, this approach is not currently possible as Sapporo automatically generates provenance from run requests and execution results without the original license information. This limitation can be overcome if data and software are consistently able to present their licenses, but this would require a generic method to get the license information of files retrieved from the internet.*<br><br>> Add an outer RO-Crate metadata file to Zenodo deposit to carry the correct licenses and pipeline licenses for each of rnaseq_1st.zip, trimming.zip, etc.<br><br>We added the License.txt and ro-crate-metadata.json files to declare the licenses in the zip archived files.<br><br>> Improve discussion to better reflect limitations of the features and its own reproducibility issues (see Q7, Q9)<br><br>We added the following paragraph to the Discussion section:<br><br>*Though Tonkaz aims to improve the reproducibility of data analysis, the system itself also has a challenge in the reproducibility of its function. The system uses the file extension to check the file type, then specifies an external tool to extract the biological features from the file to compare the workflow outputs. However, the extracted features may change by the updates in the external tools, which results in the inconsistency of the results of comparison by Tonkaz. Another issue we see in the reproducibility of the comparison is the system's dependency on Sapporo, our WES implementation. Ideally,

the results, analysis summaries, logs, etc. generated by analysis tools should be in a standardized format so any system can generate comparable statistics. The bioinformatics community needs to have a consensus for such outputs of data analysis. As a related project, MultiQC attempts to summarize the results of multiple analysis tools~\cite{ewels_multiqc_2016}. The Tonkaz system may improve its future consistency by integrating with a community effort like MultiQC, which can share the effort to extract the information from the analysis tools.*

> Consider improvements to the RO-Crate context (see Q10) - this may just be noted as Future Work in the manuscript rather than regenerating the crates

We added the following paragraph to the Discussion section:

We used RO-Crate to express the provenance of our study and added additional terms and properties to the "@context" declaration for verification purposes. These terms are currently located on our own GitHub repository, but we are discussing with the RO-Crate community moving them to a more authoritative location, such as https://github.com/ResearchObject/ro-terms. In future work, we are also considering using the Workflow Run RO-Crate profile, which is currently under development to capture the provenance of executing a computational workflow, instead of our custom terms.

> p2: Add citation for claim on file checksums different depending on software versions etc., for instance, <https://doi.org/10.1145/3186266>
> p3. "We converted Sapporo's provenance into RO-Crate" -- re-cite (20) as this is the paragraph explaining what it is.
> p10. Citations 7, 8 are missing authors
> p10. Citation 15 is now published, replace with https://doi.org/10.1145/3486897
> p0. Citations 28, 33 is missing DOI

We updated the manuscript following the reviewer's suggestion. Citations 7 and 8 miss the author information because they are the editorials without authors also in their specified citation form. We thank the reviewer for checking in detail.

Reviewer #2

> The manuscript indicates that it's not feasible to compare images automatically. However, this is pretty easy. For example, using the Pillow package in Python, you can calculate a percentage similarity between two image files. I'm not suggesting that the authors should do this in their study. But the text should not preclude this as a possibility.

We agree with the reviewer's suggestion. We updated the second paragraph of the "Automatic verification of reproducibility" section as follows:

Among the various types of output files, including analysis results, summary reports, or execution logs, the system needs to select the files to compare. We aimed to compare the analysis results that led to a biological interpretation and to avoid the comparison of the output files that are not in a standard format. Therefore, we selected the file types to be compared as an initial set and selected the corresponding EDAM ontology terms listed in Table~\ref{tab:edamFileTypes}. With this selection, for example, the nf-core RNA-seq workflow produces 872 files, but only 25 files are assigned to the EDAM ontology and compared.

> The authors describe scenarios where the outputs might be different but these differences would be immaterial to the overall conclusions. They also describe a few scenarios where the outputs differ for biological features but the differences are relatively small and could be considered to be acceptable. Examples include when BAM files are sorted differently. I think it would be helpful to add a bit more discussion of scenarios where differences in biological features could occur and what would cause those differences.

We added the following paragraph in the Discussion section:

Powered by Editorial Manager® and ProduXion Manager® from Aries Systems Corporation

In the Result section, we showed the cases where the differences are found in the outputs but the biological interpretation will be identical. However, there are cases where users find differences that affect the interpretation even when comparing the same workflow definitions. For example, the output results may change when the workflow has a tool that dynamically uses external databases, which may be regularly updated over time. Another case when the impact on the results can be observed is a comparison of runs of the workflow which does not explicitly specify the software version nor properly packaged.

> Although a person checking the outputs can change the numeric threshold, it would be difficult to know what that threshold should be. Perhaps the authors could describe the additional situation(s) where having relatively large differences would be acceptable and other situation(s) where they would not. For example, you could have a single difference in the biological feature outputs and perhaps that would make a huge difference in the interpretation in some cases. Additional discussion would be helpful.

We added the following paragraph to the Discussion section:

As the system allows users to change the reporting threshold in comparison to the outputs, users need to be aware of the acceptable differences in the outputs of the given workflow. Although the threshold needs to be low for workflows used in applications that require severe quality control, such as medical data analysis, users can set it higher for workflows that can generate different outputs per run. For example, workflows using external databases, or used for environmental monitoring purposes, may have outputs that vary per run. The system alerts when a change was found, however, as the interpretation depends on the cases, users need to understand the reason from the workflow description.

> This paper focuses on automating the verification process. I think the big picture could be explained more. Who might perform this verification process in a scientific context? In what context would they do it?

We added the following paragraph to the Discussion section:

In a scientific context, automated verification is a crucial process that should be performed for various reasons. Workflow developers can use it to easily add or update code and improve development efficiency. Administrators of workflow registries can use it to perform quality control, such as checking for broken links between the analysis tools and data used internally. Users of the workflow can also use it to validate the behavior of the workflow as an acceptance test in their own environment, thereby improving the reliability of their research projects. Tonkaz aims to support these validation efforts in different use cases and promote open science.

> Please add brief discussion about generalizing this methodology beyond Tonkaz.

We added the following paragraph to the Discussion section:

The proposed method is currently dependent on our software implementation; however, it can be generalized by the following three steps: A) Extract the statistics of biological features from the output, B) Represent the statistics in a standardized format, C) Compare the statistics, and report in the reproducibility scale. Although we implemented A and B in Sapporo, it is ideal to let workflow execution systems have those two steps rather than a WES implementation. Once steps A and B became common, step C can be implemented in many kinds of data analysis platforms, while Tonkaz only provides a CLI interface. However, the bioinformatics community needs to have a consensus on the standardized scale for reproducibility.

Again, we thank the reviewers for their comments and suggestions that improved the manuscript.

Additional Information:

Powered by Editorial Manager® and ProduXion Manager® from Aries Systems Corporation

Question	Response
Are you submitting this manuscript to a special series or article collection?	No
**Experimental design and statistics**<br><br>Full details of the experimental design and statistical methods used should be given in the Methods section, as detailed in our Minimum Standards Reporting Checklist. Information essential to interpreting the data presented should be made available in the figure legends.<br><br>Have you included all the information requested in your manuscript?	Yes
**Resources**<br><br>A description of all resources used, including antibodies, cell lines, animals and software tools, with enough information to allow them to be uniquely identified, should be included in the Methods section. Authors are strongly encouraged to cite Research Resource Identifiers (RRIDs) for antibodies, model organisms and tools, where possible.<br><br>Have you included the information requested as detailed in our Minimum Standards Reporting Checklist?	Yes
**Availability of data and materials**<br><br>All datasets and code on which the conclusions of the paper rely must be either included in your submission or deposited in publicly available repositories (where available and ethically appropriate), referencing such data using a unique identifier in the references and in the "Availability of Data and Materials" section of your manuscript.	Yes

Have you have met the above requirement as detailed in our Minimum Standards Reporting Checklist?	

Powered by Editorial Manager® and ProduXion Manager® from Aries Systems Corporation

LaTeX - Main Document (TeX file)
Click here to access/download;LaTeX - Main Document (TeX file);main.tex

```
This is pdfTeX, Version 3.141592653-2.6-1.40.24 (TeX Live 2022)
(preloaded format=pdflatex 2023.1.2)  21 APR 2023 23:39
entering extended mode
 restricted \write18 enabled.
 %&-line parsing enabled.
**main.tex
(./main.tex
LaTeX2e <2022-11-01> patch level 1
L3 programming layer <2022-12-17> (./zHenriquesLab-StyleBioRxiv.cls
Document Class `HenriquesLab' <18-April-2018>.
Document Class: zHenriquesLab-StyleBioRxiv 2018/04/18 revision by RH
(c:/TeXLive/2022/texmf-dist/tex/latex/base/article.cls
Document Class: article 2022/07/02 v1.4n Standard LaTeX document class
(c:/TeXLive/2022/texmf-dist/tex/latex/base/size10.clo
File: size10.clo 2022/07/02 v1.4n Standard LaTeX file (size option)
)
\c@part=\count185
\c@section=\count186
\c@subsection=\count187
\c@subsubsection=\count188
\c@paragraph=\count189
\c@subparagraph=\count190
\c@figure=\count191
\c@table=\count192
\abovecaptionskip=\skip48
\belowcaptionskip=\skip49
\bibindent=\dimen140
) (c:/TeXLive/2022/texmf-dist/tex/latex/amsmath/amsmath.sty
Package: amsmath 2022/04/08 v2.17n AMS math features
\@mathmargin=\skip50
For additional information on amsmath, use the `?' option.
(c:/TeXLive/2022/texmf-dist/tex/latex/amsmath/amstext.sty
Package: amstext 2021/08/26 v2.01 AMS text
(c:/TeXLive/2022/texmf-dist/tex/latex/amsmath/amsgen.sty
File: amsgen.sty 1999/11/30 v2.0 generic functions
\@emptytoks=\toks16
\ex@=\dimen141
)) (c:/TeXLive/2022/texmf-dist/tex/latex/amsmath/amsbsy.sty
Package: amsbsy 1999/11/29 v1.2d Bold Symbols
\pmbraise@=\dimen142
) (c:/TeXLive/2022/texmf-dist/tex/latex/amsmath/amsopn.sty
Package: amsopn 2022/04/08 v2.04 operator names
)
\inf@bad=\count193
LaTeX Info: Redefining \frac on input line 234.
\uproot@=\count194
\leftroot@=\count195
LaTeX Info: Redefining \overline on input line 399.
LaTeX Info: Redefining \colon on input line 410.
\classnum@=\count196
\DOTSCASE@=\count197
LaTeX Info: Redefining \ldots on input line 496.
LaTeX Info: Redefining \dots on input line 499.
LaTeX Info: Redefining \cdots on input line 620.
```

```
\Mathstrutbox@=\box51
\strutbox@=\box52
LaTeX Info: Redefining \big on input line 722.
LaTeX Info: Redefining \Big on input line 723.
LaTeX Info: Redefining \bigg on input line 724.
LaTeX Info: Redefining \Bigg on input line 725.
\big@size=\dimen143
LaTeX Font Info:    Redeclaring font encoding OML on input line 743.
LaTeX Font Info:    Redeclaring font encoding OMS on input line 744.
\macc@depth=\count198
LaTeX Info: Redefining \bmod on input line 905.
LaTeX Info: Redefining \pmod on input line 910.
LaTeX Info: Redefining \smash on input line 940.
LaTeX Info: Redefining \relbar on input line 970.
LaTeX Info: Redefining \Relbar on input line 971.
\c@MaxMatrixCols=\count199
\dotsspace@=\muskip16
\c@parentequation=\count266
\dspbrk@lvl=\count267
\tag@help=\toks17
\row@=\count268
\column@=\count269
\maxfields@=\count270
\andhelp@=\toks18
\eqnshift@=\dimen144
\alignsep@=\dimen145
\tagshift@=\dimen146
\tagwidth@=\dimen147
\totwidth@=\dimen148
\lineht@=\dimen149
\@envbody=\toks19
\multlinegap=\skip51
\multlinetaggap=\skip52
\mathdisplay@stack=\toks20
LaTeX Info: Redefining \[ on input line 2953.
LaTeX Info: Redefining \] on input line 2954.
) (c:/TeXLive/2022/texmf-dist/tex/latex/amsfonts/amsfonts.sty
Package: amsfonts 2013/01/14 v3.01 Basic AMSFonts support
\symAMSa=\mathgroup4
\symAMSb=\mathgroup5
LaTeX Font Info:    Redeclaring math symbol \hbar on input line 98.
LaTeX Font Info:    Overwriting math alphabet `\mathfrak' in version
`bold'
(Font)                  U/euf/m/n --> U/euf/b/n on input line 106.
) (c:/TeXLive/2022/texmf-dist/tex/latex/amsfonts/amssymb.sty
Package: amssymb 2013/01/14 v3.01 AMS font symbols
) (c:/TeXLive/2022/texmf-dist/tex/latex/psnfss/mathptmx.sty
Package: mathptmx 2020/03/25 PSNFSS-v9.3 Times w/ Math, improved (SPQR,
WaS)
LaTeX Font Info:    Redeclaring symbol font `operators' on input line 28.
LaTeX Font Info:    Overwriting symbol font `operators' in version
`normal'
(Font)                  OT1/cmr/m/n --> OT1/ztmcm/m/n on input line 28.
LaTeX Font Info:    Overwriting symbol font `operators' in version `bold'
```

```
(Font)                  OT1/cmr/bx/n --> OT1/ztmcm/m/n on input line 28.
LaTeX Font Info:    Redeclaring symbol font `letters' on input line 29.
LaTeX Font Info:    Overwriting symbol font `letters' in version `normal'
(Font)                  OML/cmm/m/it --> OML/ztmcm/m/it on input line 29.
LaTeX Font Info:    Overwriting symbol font `letters' in version `bold'
(Font)                  OML/cmm/b/it --> OML/ztmcm/m/it on input line 29.
LaTeX Font Info:    Redeclaring symbol font `symbols' on input line 30.
LaTeX Font Info:    Overwriting symbol font `symbols' in version `normal'
(Font)                  OMS/cmsy/m/n --> OMS/ztmcm/m/n on input line 30.
LaTeX Font Info:    Overwriting symbol font `symbols' in version `bold'
(Font)                  OMS/cmsy/b/n --> OMS/ztmcm/m/n on input line 30.
LaTeX Font Info:    Redeclaring symbol font `largesymbols' on input line
31.
LaTeX Font Info:    Overwriting symbol font `largesymbols' in version
`normal'
(Font)                  OMX/cmex/m/n --> OMX/ztmcm/m/n on input line 31.
LaTeX Font Info:    Overwriting symbol font `largesymbols' in version
`bold'
(Font)                  OMX/cmex/m/n --> OMX/ztmcm/m/n on input line 31.
\symbold=\mathgroup6
\symitalic=\mathgroup7
LaTeX Font Info:    Redeclaring math alphabet \mathbf on input line 34.
LaTeX Font Info:    Overwriting math alphabet `\mathbf' in version
`normal'
(Font)                  OT1/cmr/bx/n --> OT1/ptm/bx/n on input line 34.
LaTeX Font Info:    Overwriting math alphabet `\mathbf' in version `bold'
(Font)                  OT1/cmr/bx/n --> OT1/ptm/bx/n on input line 34.
LaTeX Font Info:    Redeclaring math alphabet \mathit on input line 35.
LaTeX Font Info:    Overwriting math alphabet `\mathit' in version
`normal'
(Font)                  OT1/cmr/m/it --> OT1/ptm/m/it on input line 35.
LaTeX Font Info:    Overwriting math alphabet `\mathit' in version `bold'
(Font)                  OT1/cmr/bx/it --> OT1/ptm/m/it on input line 35.
LaTeX Info: Redefining \hbar on input line 50.
) (c:/TeXLive/2022/texmf-dist/tex/latex/hyperref/hyperref.sty
Package: hyperref 2023-02-07 v7.00v Hypertext links for LaTeX
(c:/TeXLive/2022/texmf-dist/tex/generic/ltxcmds/ltxcmds.sty
Package: ltxcmds 2020-05-10 v1.25 LaTeX kernel commands for general use
(HO)
) (c:/TeXLive/2022/texmf-dist/tex/generic/iftex/iftex.sty
Package: iftex 2022/02/03 v1.0f TeX engine tests
) (c:/TeXLive/2022/texmf-dist/tex/generic/pdftexcmds/pdftexcmds.sty
Package: pdftexcmds 2020-06-27 v0.33 Utility functions of pdfTeX for
LuaTeX (HO
)
(c:/TeXLive/2022/texmf-dist/tex/generic/infwarerr/infwarerr.sty
Package: infwarerr 2019/12/03 v1.5 Providing info/warning/error messages
(HO)
)
Package pdftexcmds Info: \pdf@primitive is available.
Package pdftexcmds Info: \pdf@ifprimitive is available.
Package pdftexcmds Info: \pdfdraftmode found.
) (c:/TeXLive/2022/texmf-dist/tex/latex/graphics/keyval.sty
Package: keyval 2022/05/29 v1.15 key=value parser (DPC)
```

```
\KV@toks@=\toks21
) (c:/TeXLive/2022/texmf-dist/tex/latex/kvsetkeys/kvsetkeys.sty
Package: kvsetkeys 2022-10-05 v1.19 Key value parser (HO)
) (c:/TeXLive/2022/texmf-dist/tex/generic/kvdefinekeys/kvdefinekeys.sty
Package: kvdefinekeys 2019-12-19 v1.6 Define keys (HO)
) (c:/TeXLive/2022/texmf-dist/tex/generic/pdfescape/pdfescape.sty
Package: pdfescape 2019/12/09 v1.15 Implements pdfTeX's escape features
(HO)
) (c:/TeXLive/2022/texmf-dist/tex/latex/hycolor/hycolor.sty
Package: hycolor 2020-01-27 v1.10 Color options for hyperref/bookmark
(HO)
) (c:/TeXLive/2022/texmf-dist/tex/latex/letltxmacro/letltxmacro.sty
Package: letltxmacro 2019/12/03 v1.6 Let assignment for LaTeX macros (HO)
) (c:/TeXLive/2022/texmf-dist/tex/latex/auxhook/auxhook.sty
Package: auxhook 2019-12-17 v1.6 Hooks for auxiliary files (HO)
) (c:/TeXLive/2022/texmf-dist/tex/latex/hyperref/nameref.sty
Package: nameref 2022-05-17 v2.50 Cross-referencing by name of section
(c:/TeXLive/2022/texmf-dist/tex/latex/refcount/refcount.sty
Package: refcount 2019/12/15 v3.6 Data extraction from label references
(HO)
) (c:/TeXLive/2022/texmf-
dist/tex/generic/gettitlestring/gettitlestring.sty
Package: gettitlestring 2019/12/15 v1.6 Cleanup title references (HO)
(c:/TeXLive/2022/texmf-dist/tex/latex/kvoptions/kvoptions.sty
Package: kvoptions 2022-06-15 v3.15 Key value format for package options
(HO)
))
\c@section@level=\count271
)
\@linkdim=\dimen150
\Hy@linkcounter=\count272
\Hy@pagecounter=\count273
(c:/TeXLive/2022/texmf-dist/tex/latex/hyperref/pd1enc.def
File: pd1enc.def 2023-02-07 v7.00v Hyperref: PDFDocEncoding definition
(HO)
Now handling font encoding PD1 ...
... no UTF-8 mapping file for font encoding PD1
) (c:/TeXLive/2022/texmf-dist/tex/generic/intcalc/intcalc.sty
Package: intcalc 2019/12/15 v1.3 Expandable calculations with integers
(HO)
) (c:/TeXLive/2022/texmf-dist/tex/generic/etexcmds/etexcmds.sty
Package: etexcmds 2019/12/15 v1.7 Avoid name clashes with e-TeX commands
(HO)
)
\Hy@SavedSpaceFactor=\count274
(c:/TeXLive/2022/texmf-dist/tex/latex/hyperref/puenc.def
File: puenc.def 2023-02-07 v7.00v Hyperref: PDF Unicode definition (HO)
Now handling font encoding PU ...
... no UTF-8 mapping file for font encoding PU
)
Package hyperref Info: Option `colorlinks' set `true' on input line 4060.
Package hyperref Info: Hyper figures OFF on input line 4177.
Package hyperref Info: Link nesting OFF on input line 4182.
Package hyperref Info: Hyper index ON on input line 4185.
```

```
Package hyperref Info: Plain pages OFF on input line 4192.
Package hyperref Info: Backreferencing OFF on input line 4197.
Package hyperref Info: Implicit mode ON; LaTeX internals redefined.
Package hyperref Info: Bookmarks ON on input line 4425.
\c@Hy@tempcnt=\count275
(c:/TeXLive/2022/texmf-dist/tex/latex/url/url.sty
\Urlmuskip=\muskip17
Package: url 2013/09/16  ver 3.4  Verb mode for urls, etc.
)
LaTeX Info: Redefining \url on input line 4763.
\XeTeXLinkMargin=\dimen151
(c:/TeXLive/2022/texmf-dist/tex/generic/bitset/bitset.sty
Package: bitset 2019/12/09 v1.3 Handle bit-vector datatype (HO)
(c:/TeXLive/2022/texmf-dist/tex/generic/bigintcalc/bigintcalc.sty
Package: bigintcalc 2019/12/15 v1.5 Expandable calculations on big
integers (HO
)
))
\Fld@menulength=\count276
\Field@Width=\dimen152
\Fld@charsize=\dimen153
Package hyperref Info: Hyper figures OFF on input line 6042.
Package hyperref Info: Link nesting OFF on input line 6047.
Package hyperref Info: Hyper index ON on input line 6050.
Package hyperref Info: backreferencing OFF on input line 6057.
Package hyperref Info: Link coloring ON on input line 6060.
Package hyperref Info: Link coloring with OCG OFF on input line 6067.
Package hyperref Info: PDF/A mode OFF on input line 6072.
(c:/TeXLive/2022/texmf-dist/tex/latex/base/atbegshi-ltx.sty
Package: atbegshi-ltx 2021/01/10 v1.0c Emulation of the original atbegshi
package with kernel methods
)
\Hy@abspage=\count277
\c@Item=\count278
\c@Hfootnote=\count279
)
Package hyperref Info: Driver (autodetected): hpdftex.
(c:/TeXLive/2022/texmf-dist/tex/latex/hyperref/hpdftex.def
File: hpdftex.def 2023-02-07 v7.00v Hyperref driver for pdfTeX
(c:/TeXLive/2022/texmf-dist/tex/latex/base/atveryend-ltx.sty
Package: atveryend-ltx 2020/08/19 v1.0a Emulation of the original
atveryend pac
kage
with kernel methods
)
\Fld@listcount=\count280
\c@bookmark@seq@number=\count281
(c:/TeXLive/2022/texmf-dist/tex/latex/rerunfilecheck/rerunfilecheck.sty
Package: rerunfilecheck 2022-07-10 v1.10 Rerun checks for auxiliary files
(HO)
(c:/TeXLive/2022/texmf-dist/tex/generic/uniquecounter/uniquecounter.sty
Package: uniquecounter 2019/12/15 v1.4 Provide unlimited unique counter
(HO)
)
```

```
Package uniquecounter Info: New unique counter `rerunfilecheck' on input
line 2
85.
)
\Hy@SectionHShift=\skip53
) (c:/TeXLive/2022/texmf-dist/tex/latex/xcolor/xcolor.sty
Package: xcolor 2022/06/12 v2.14 LaTeX color extensions (UK)
(c:/TeXLive/2022/texmf-dist/tex/latex/graphics-cfg/color.cfg
File: color.cfg 2016/01/02 v1.6 sample color configuration
)
Package xcolor Info: Driver file: pdftex.def on input line 227.
(c:/TeXLive/2022/texmf-dist/tex/latex/graphics-def/pdftex.def
File: pdftex.def 2022/09/22 v1.2b Graphics/color driver for pdftex
) (c:/TeXLive/2022/texmf-dist/tex/latex/graphics/mathcolor.ltx)
Package xcolor Info: Model `cmy' substituted by `cmy0' on input line
1353.
Package xcolor Info: Model `hsb' substituted by `rgb' on input line 1357.
Package xcolor Info: Model `RGB' extended on input line 1369.
Package xcolor Info: Model `HTML' substituted by `rgb' on input line
1371.
Package xcolor Info: Model `Hsb' substituted by `hsb' on input line 1372.
Package xcolor Info: Model `tHsb' substituted by `hsb' on input line
1373.
Package xcolor Info: Model `HSB' substituted by `hsb' on input line 1374.
Package xcolor Info: Model `Gray' substituted by `gray' on input line
1375.
Package xcolor Info: Model `wave' substituted by `hsb' on input line
1376.
) (c:/TeXLive/2022/texmf-dist/tex/latex/preprint/authblk.sty
Package: authblk 2001/02/27 1.3 (PWD)
\affilsep=\skip54
\@affilsep=\skip55
\c@Maxaffil=\count282
\c@authors=\count283
\c@affil=\count284
) (c:/TeXLive/2022/texmf-dist/tex/latex/base/inputenc.sty
Package: inputenc 2021/02/14 v1.3d Input encoding file
\inpenc@prehook=\toks22
\inpenc@posthook=\toks23
(c:/TeXLive/2022/texmf-dist/tex/latex/base/latin1.def
File: latin1.def 2021/02/14 v1.3d Input encoding file
) (c:/TeXLive/2022/texmf-dist/tex/latex/base/utf8.def
File: utf8.def 2022/06/07 v1.3c UTF-8 support
Now handling font encoding OML ...
... no UTF-8 mapping file for font encoding OML
Now handling font encoding OMS ...
... processing UTF-8 mapping file for font encoding OMS
(c:/TeXLive/2022/texmf-dist/tex/latex/base/omsenc.dfu
File: omsenc.dfu 2022/06/07 v1.3c UTF-8 support
   defining Unicode char U+00A7 (decimal 167)
   defining Unicode char U+00B6 (decimal 182)
   defining Unicode char U+00B7 (decimal 183)
   defining Unicode char U+2020 (decimal 8224)
   defining Unicode char U+2021 (decimal 8225)
```

```
   defining Unicode char U+2022 (decimal 8226)
)
Now handling font encoding OT1 ...
... processing UTF-8 mapping file for font encoding OT1
(c:/TeXLive/2022/texmf-dist/tex/latex/base/ot1enc.dfu
File: ot1enc.dfu 2022/06/07 v1.3c UTF-8 support
   defining Unicode char U+00A0 (decimal 160)
   defining Unicode char U+00A1 (decimal 161)
   defining Unicode char U+00A3 (decimal 163)
   defining Unicode char U+00AD (decimal 173)
   defining Unicode char U+00B8 (decimal 184)
   defining Unicode char U+00BF (decimal 191)
   defining Unicode char U+00C5 (decimal 197)
   defining Unicode char U+00C6 (decimal 198)
   defining Unicode char U+00D8 (decimal 216)
   defining Unicode char U+00DF (decimal 223)
   defining Unicode char U+00E6 (decimal 230)
   defining Unicode char U+00EC (decimal 236)
   defining Unicode char U+00ED (decimal 237)
   defining Unicode char U+00EE (decimal 238)
   defining Unicode char U+00EF (decimal 239)
   defining Unicode char U+00F8 (decimal 248)
   defining Unicode char U+0131 (decimal 305)
   defining Unicode char U+0141 (decimal 321)
   defining Unicode char U+0142 (decimal 322)
   defining Unicode char U+0152 (decimal 338)
   defining Unicode char U+0153 (decimal 339)
   defining Unicode char U+0174 (decimal 372)
   defining Unicode char U+0175 (decimal 373)
   defining Unicode char U+0176 (decimal 374)
   defining Unicode char U+0177 (decimal 375)
   defining Unicode char U+0218 (decimal 536)
   defining Unicode char U+0219 (decimal 537)
   defining Unicode char U+021A (decimal 538)
   defining Unicode char U+021B (decimal 539)
   defining Unicode char U+0237 (decimal 567)
   defining Unicode char U+2013 (decimal 8211)
   defining Unicode char U+2014 (decimal 8212)
   defining Unicode char U+2018 (decimal 8216)
   defining Unicode char U+2019 (decimal 8217)
   defining Unicode char U+201C (decimal 8220)
   defining Unicode char U+201D (decimal 8221)
   defining Unicode char U+FB00 (decimal 64256)
   defining Unicode char U+FB01 (decimal 64257)
   defining Unicode char U+FB02 (decimal 64258)
   defining Unicode char U+FB03 (decimal 64259)
   defining Unicode char U+FB04 (decimal 64260)
   defining Unicode char U+FB05 (decimal 64261)
   defining Unicode char U+FB06 (decimal 64262)
)
Now handling font encoding T1 ...
... processing UTF-8 mapping file for font encoding T1
(c:/TeXLive/2022/texmf-dist/tex/latex/base/t1enc.dfu
File: t1enc.dfu 2022/06/07 v1.3c UTF-8 support
```

```
defining Unicode char U+00A0 (decimal 160)
defining Unicode char U+00A1 (decimal 161)
defining Unicode char U+00A3 (decimal 163)
defining Unicode char U+00AB (decimal 171)
defining Unicode char U+00AD (decimal 173)
defining Unicode char U+00BB (decimal 187)
defining Unicode char U+00BF (decimal 191)
defining Unicode char U+00C0 (decimal 192)
defining Unicode char U+00C1 (decimal 193)
defining Unicode char U+00C2 (decimal 194)
defining Unicode char U+00C3 (decimal 195)
defining Unicode char U+00C4 (decimal 196)
defining Unicode char U+00C5 (decimal 197)
defining Unicode char U+00C6 (decimal 198)
defining Unicode char U+00C7 (decimal 199)
defining Unicode char U+00C8 (decimal 200)
defining Unicode char U+00C9 (decimal 201)
defining Unicode char U+00CA (decimal 202)
defining Unicode char U+00CB (decimal 203)
defining Unicode char U+00CC (decimal 204)
defining Unicode char U+00CD (decimal 205)
defining Unicode char U+00CE (decimal 206)
defining Unicode char U+00CF (decimal 207)
defining Unicode char U+00D0 (decimal 208)
defining Unicode char U+00D1 (decimal 209)
defining Unicode char U+00D2 (decimal 210)
defining Unicode char U+00D3 (decimal 211)
defining Unicode char U+00D4 (decimal 212)
defining Unicode char U+00D5 (decimal 213)
defining Unicode char U+00D6 (decimal 214)
defining Unicode char U+00D8 (decimal 216)
defining Unicode char U+00D9 (decimal 217)
defining Unicode char U+00DA (decimal 218)
defining Unicode char U+00DB (decimal 219)
defining Unicode char U+00DC (decimal 220)
defining Unicode char U+00DD (decimal 221)
defining Unicode char U+00DE (decimal 222)
defining Unicode char U+00DF (decimal 223)
defining Unicode char U+00E0 (decimal 224)
defining Unicode char U+00E1 (decimal 225)
defining Unicode char U+00E2 (decimal 226)
defining Unicode char U+00E3 (decimal 227)
defining Unicode char U+00E4 (decimal 228)
defining Unicode char U+00E5 (decimal 229)
defining Unicode char U+00E6 (decimal 230)
defining Unicode char U+00E7 (decimal 231)
defining Unicode char U+00E8 (decimal 232)
defining Unicode char U+00E9 (decimal 233)
defining Unicode char U+00EA (decimal 234)
defining Unicode char U+00EB (decimal 235)
defining Unicode char U+00EC (decimal 236)
defining Unicode char U+00ED (decimal 237)
defining Unicode char U+00EE (decimal 238)
defining Unicode char U+00EF (decimal 239)
```

```
defining Unicode char U+00F0 (decimal 240)
defining Unicode char U+00F1 (decimal 241)
defining Unicode char U+00F2 (decimal 242)
defining Unicode char U+00F3 (decimal 243)
defining Unicode char U+00F4 (decimal 244)
defining Unicode char U+00F5 (decimal 245)
defining Unicode char U+00F6 (decimal 246)
defining Unicode char U+00F8 (decimal 248)
defining Unicode char U+00F9 (decimal 249)
defining Unicode char U+00FA (decimal 250)
defining Unicode char U+00FB (decimal 251)
defining Unicode char U+00FC (decimal 252)
defining Unicode char U+00FD (decimal 253)
defining Unicode char U+00FE (decimal 254)
defining Unicode char U+00FF (decimal 255)
defining Unicode char U+0100 (decimal 256)
defining Unicode char U+0101 (decimal 257)
defining Unicode char U+0102 (decimal 258)
defining Unicode char U+0103 (decimal 259)
defining Unicode char U+0104 (decimal 260)
defining Unicode char U+0105 (decimal 261)
defining Unicode char U+0106 (decimal 262)
defining Unicode char U+0107 (decimal 263)
defining Unicode char U+0108 (decimal 264)
defining Unicode char U+0109 (decimal 265)
defining Unicode char U+010A (decimal 266)
defining Unicode char U+010B (decimal 267)
defining Unicode char U+010C (decimal 268)
defining Unicode char U+010D (decimal 269)
defining Unicode char U+010E (decimal 270)
defining Unicode char U+010F (decimal 271)
defining Unicode char U+0110 (decimal 272)
defining Unicode char U+0111 (decimal 273)
defining Unicode char U+0112 (decimal 274)
defining Unicode char U+0113 (decimal 275)
defining Unicode char U+0114 (decimal 276)
defining Unicode char U+0115 (decimal 277)
defining Unicode char U+0116 (decimal 278)
defining Unicode char U+0117 (decimal 279)
defining Unicode char U+0118 (decimal 280)
defining Unicode char U+0119 (decimal 281)
defining Unicode char U+011A (decimal 282)
defining Unicode char U+011B (decimal 283)
defining Unicode char U+011C (decimal 284)
defining Unicode char U+011D (decimal 285)
defining Unicode char U+011E (decimal 286)
defining Unicode char U+011F (decimal 287)
defining Unicode char U+0120 (decimal 288)
defining Unicode char U+0121 (decimal 289)
defining Unicode char U+0122 (decimal 290)
defining Unicode char U+0123 (decimal 291)
defining Unicode char U+0124 (decimal 292)
defining Unicode char U+0125 (decimal 293)
defining Unicode char U+0128 (decimal 296)
```

```
defining Unicode char U+0129 (decimal 297)
defining Unicode char U+012A (decimal 298)
defining Unicode char U+012B (decimal 299)
defining Unicode char U+012C (decimal 300)
defining Unicode char U+012D (decimal 301)
defining Unicode char U+012E (decimal 302)
defining Unicode char U+012F (decimal 303)
defining Unicode char U+0130 (decimal 304)
defining Unicode char U+0131 (decimal 305)
defining Unicode char U+0132 (decimal 306)
defining Unicode char U+0133 (decimal 307)
defining Unicode char U+0134 (decimal 308)
defining Unicode char U+0135 (decimal 309)
defining Unicode char U+0136 (decimal 310)
defining Unicode char U+0137 (decimal 311)
defining Unicode char U+0139 (decimal 313)
defining Unicode char U+013A (decimal 314)
defining Unicode char U+013B (decimal 315)
defining Unicode char U+013C (decimal 316)
defining Unicode char U+013D (decimal 317)
defining Unicode char U+013E (decimal 318)
defining Unicode char U+0141 (decimal 321)
defining Unicode char U+0142 (decimal 322)
defining Unicode char U+0143 (decimal 323)
defining Unicode char U+0144 (decimal 324)
defining Unicode char U+0145 (decimal 325)
defining Unicode char U+0146 (decimal 326)
defining Unicode char U+0147 (decimal 327)
defining Unicode char U+0148 (decimal 328)
defining Unicode char U+014A (decimal 330)
defining Unicode char U+014B (decimal 331)
defining Unicode char U+014C (decimal 332)
defining Unicode char U+014D (decimal 333)
defining Unicode char U+014E (decimal 334)
defining Unicode char U+014F (decimal 335)
defining Unicode char U+0150 (decimal 336)
defining Unicode char U+0151 (decimal 337)
defining Unicode char U+0152 (decimal 338)
defining Unicode char U+0153 (decimal 339)
defining Unicode char U+0154 (decimal 340)
defining Unicode char U+0155 (decimal 341)
defining Unicode char U+0156 (decimal 342)
defining Unicode char U+0157 (decimal 343)
defining Unicode char U+0158 (decimal 344)
defining Unicode char U+0159 (decimal 345)
defining Unicode char U+015A (decimal 346)
defining Unicode char U+015B (decimal 347)
defining Unicode char U+015C (decimal 348)
defining Unicode char U+015D (decimal 349)
defining Unicode char U+015E (decimal 350)
defining Unicode char U+015F (decimal 351)
defining Unicode char U+0160 (decimal 352)
defining Unicode char U+0161 (decimal 353)
defining Unicode char U+0162 (decimal 354)
```

```
defining Unicode char U+0163 (decimal 355)
defining Unicode char U+0164 (decimal 356)
defining Unicode char U+0165 (decimal 357)
defining Unicode char U+0168 (decimal 360)
defining Unicode char U+0169 (decimal 361)
defining Unicode char U+016A (decimal 362)
defining Unicode char U+016B (decimal 363)
defining Unicode char U+016C (decimal 364)
defining Unicode char U+016D (decimal 365)
defining Unicode char U+016E (decimal 366)
defining Unicode char U+016F (decimal 367)
defining Unicode char U+0170 (decimal 368)
defining Unicode char U+0171 (decimal 369)
defining Unicode char U+0172 (decimal 370)
defining Unicode char U+0173 (decimal 371)
defining Unicode char U+0174 (decimal 372)
defining Unicode char U+0175 (decimal 373)
defining Unicode char U+0176 (decimal 374)
defining Unicode char U+0177 (decimal 375)
defining Unicode char U+0178 (decimal 376)
defining Unicode char U+0179 (decimal 377)
defining Unicode char U+017A (decimal 378)
defining Unicode char U+017B (decimal 379)
defining Unicode char U+017C (decimal 380)
defining Unicode char U+017D (decimal 381)
defining Unicode char U+017E (decimal 382)
defining Unicode char U+01C4 (decimal 452)
defining Unicode char U+01C5 (decimal 453)
defining Unicode char U+01C6 (decimal 454)
defining Unicode char U+01C7 (decimal 455)
defining Unicode char U+01C8 (decimal 456)
defining Unicode char U+01C9 (decimal 457)
defining Unicode char U+01CA (decimal 458)
defining Unicode char U+01CB (decimal 459)
defining Unicode char U+01CC (decimal 460)
defining Unicode char U+01CD (decimal 461)
defining Unicode char U+01CE (decimal 462)
defining Unicode char U+01CF (decimal 463)
defining Unicode char U+01D0 (decimal 464)
defining Unicode char U+01D1 (decimal 465)
defining Unicode char U+01D2 (decimal 466)
defining Unicode char U+01D3 (decimal 467)
defining Unicode char U+01D4 (decimal 468)
defining Unicode char U+01E2 (decimal 482)
defining Unicode char U+01E3 (decimal 483)
defining Unicode char U+01E6 (decimal 486)
defining Unicode char U+01E7 (decimal 487)
defining Unicode char U+01E8 (decimal 488)
defining Unicode char U+01E9 (decimal 489)
defining Unicode char U+01EA (decimal 490)
defining Unicode char U+01EB (decimal 491)
defining Unicode char U+01F0 (decimal 496)
defining Unicode char U+01F4 (decimal 500)
defining Unicode char U+01F5 (decimal 501)
```

```
defining Unicode char U+0218 (decimal 536)
defining Unicode char U+0219 (decimal 537)
defining Unicode char U+021A (decimal 538)
defining Unicode char U+021B (decimal 539)
defining Unicode char U+0232 (decimal 562)
defining Unicode char U+0233 (decimal 563)
defining Unicode char U+0237 (decimal 567)
defining Unicode char U+02D9 (decimal 729)
defining Unicode char U+02DB (decimal 731)
defining Unicode char U+1E02 (decimal 7682)
defining Unicode char U+1E03 (decimal 7683)
defining Unicode char U+1E0D (decimal 7693)
defining Unicode char U+1E1E (decimal 7710)
defining Unicode char U+1E1F (decimal 7711)
defining Unicode char U+1E25 (decimal 7717)
defining Unicode char U+1E30 (decimal 7728)
defining Unicode char U+1E31 (decimal 7729)
defining Unicode char U+1E37 (decimal 7735)
defining Unicode char U+1E8E (decimal 7822)
defining Unicode char U+1E8F (decimal 7823)
defining Unicode char U+1E43 (decimal 7747)
defining Unicode char U+1E45 (decimal 7749)
defining Unicode char U+1E47 (decimal 7751)
defining Unicode char U+1E5B (decimal 7771)
defining Unicode char U+1E63 (decimal 7779)
defining Unicode char U+1E6D (decimal 7789)
defining Unicode char U+1E90 (decimal 7824)
defining Unicode char U+1E91 (decimal 7825)
defining Unicode char U+1E9E (decimal 7838)
defining Unicode char U+1EF2 (decimal 7922)
defining Unicode char U+1EF3 (decimal 7923)
defining Unicode char U+200C (decimal 8204)
defining Unicode char U+2010 (decimal 8208)
defining Unicode char U+2011 (decimal 8209)
defining Unicode char U+2012 (decimal 8210)
defining Unicode char U+2013 (decimal 8211)
defining Unicode char U+2014 (decimal 8212)
defining Unicode char U+2015 (decimal 8213)
defining Unicode char U+2018 (decimal 8216)
defining Unicode char U+2019 (decimal 8217)
defining Unicode char U+201A (decimal 8218)
defining Unicode char U+201C (decimal 8220)
defining Unicode char U+201D (decimal 8221)
defining Unicode char U+201E (decimal 8222)
defining Unicode char U+2030 (decimal 8240)
defining Unicode char U+2031 (decimal 8241)
defining Unicode char U+2039 (decimal 8249)
defining Unicode char U+203A (decimal 8250)
defining Unicode char U+2423 (decimal 9251)
defining Unicode char U+1E20 (decimal 7712)
defining Unicode char U+1E21 (decimal 7713)
defining Unicode char U+FB00 (decimal 64256)
defining Unicode char U+FB01 (decimal 64257)
defining Unicode char U+FB02 (decimal 64258)
```

```
   defining Unicode char U+FB03 (decimal 64259)
   defining Unicode char U+FB04 (decimal 64260)
   defining Unicode char U+FB05 (decimal 64261)
   defining Unicode char U+FB06 (decimal 64262)
)
Now handling font encoding TS1 ...
... processing UTF-8 mapping file for font encoding TS1
(c:/TeXLive/2022/texmf-dist/tex/latex/base/ts1enc.dfu
File: ts1enc.dfu 2022/06/07 v1.3c UTF-8 support
   defining Unicode char U+00A2 (decimal 162)
   defining Unicode char U+00A3 (decimal 163)
   defining Unicode char U+00A4 (decimal 164)
   defining Unicode char U+00A5 (decimal 165)
   defining Unicode char U+00A6 (decimal 166)
   defining Unicode char U+00A7 (decimal 167)
   defining Unicode char U+00A8 (decimal 168)
   defining Unicode char U+00A9 (decimal 169)
   defining Unicode char U+00AA (decimal 170)
   defining Unicode char U+00AC (decimal 172)
   defining Unicode char U+00AE (decimal 174)
   defining Unicode char U+00AF (decimal 175)
   defining Unicode char U+00B0 (decimal 176)
   defining Unicode char U+00B1 (decimal 177)
   defining Unicode char U+00B2 (decimal 178)
   defining Unicode char U+00B3 (decimal 179)
   defining Unicode char U+00B4 (decimal 180)
   defining Unicode char U+00B5 (decimal 181)
   defining Unicode char U+00B6 (decimal 182)
   defining Unicode char U+00B7 (decimal 183)
   defining Unicode char U+00B9 (decimal 185)
   defining Unicode char U+00BA (decimal 186)
   defining Unicode char U+00BC (decimal 188)
   defining Unicode char U+00BD (decimal 189)
   defining Unicode char U+00BE (decimal 190)
   defining Unicode char U+00D7 (decimal 215)
   defining Unicode char U+00F7 (decimal 247)
   defining Unicode char U+0192 (decimal 402)
   defining Unicode char U+02C7 (decimal 711)
   defining Unicode char U+02D8 (decimal 728)
   defining Unicode char U+02DD (decimal 733)
   defining Unicode char U+0E3F (decimal 3647)
   defining Unicode char U+2016 (decimal 8214)
   defining Unicode char U+2020 (decimal 8224)
   defining Unicode char U+2021 (decimal 8225)
   defining Unicode char U+2022 (decimal 8226)
   defining Unicode char U+2030 (decimal 8240)
   defining Unicode char U+2031 (decimal 8241)
   defining Unicode char U+203B (decimal 8251)
   defining Unicode char U+203D (decimal 8253)
   defining Unicode char U+2044 (decimal 8260)
   defining Unicode char U+204E (decimal 8270)
   defining Unicode char U+2052 (decimal 8274)
   defining Unicode char U+20A1 (decimal 8353)
   defining Unicode char U+20A4 (decimal 8356)
```

```
   defining Unicode char U+20A6 (decimal 8358)
   defining Unicode char U+20A9 (decimal 8361)
   defining Unicode char U+20AB (decimal 8363)
   defining Unicode char U+20AC (decimal 8364)
   defining Unicode char U+20B1 (decimal 8369)
   defining Unicode char U+2103 (decimal 8451)
   defining Unicode char U+2116 (decimal 8470)
   defining Unicode char U+2117 (decimal 8471)
   defining Unicode char U+211E (decimal 8478)
   defining Unicode char U+2120 (decimal 8480)
   defining Unicode char U+2122 (decimal 8482)
   defining Unicode char U+2126 (decimal 8486)
   defining Unicode char U+2127 (decimal 8487)
   defining Unicode char U+212E (decimal 8494)
   defining Unicode char U+2190 (decimal 8592)
   defining Unicode char U+2191 (decimal 8593)
   defining Unicode char U+2192 (decimal 8594)
   defining Unicode char U+2193 (decimal 8595)
   defining Unicode char U+2329 (decimal 9001)
   defining Unicode char U+3008 (decimal 12296)
   defining Unicode char U+232A (decimal 9002)
   defining Unicode char U+3009 (decimal 12297)
   defining Unicode char U+2422 (decimal 9250)
   defining Unicode char U+25E6 (decimal 9702)
   defining Unicode char U+25EF (decimal 9711)
   defining Unicode char U+266A (decimal 9834)
   defining Unicode char U+27E8 (decimal 10216)
   defining Unicode char U+27E9 (decimal 10217)
   defining Unicode char U+FEFF (decimal 65279)
)
Now handling font encoding OMX ...
... no UTF-8 mapping file for font encoding OMX
Now handling font encoding U ...
... no UTF-8 mapping file for font encoding U
Now handling font encoding PD1 ...
... no UTF-8 mapping file for font encoding PD1
Now handling font encoding PU ...
... no UTF-8 mapping file for font encoding PU
   defining Unicode char U+00A9 (decimal 169)
   defining Unicode char U+00AA (decimal 170)
   defining Unicode char U+00AE (decimal 174)
   defining Unicode char U+00BA (decimal 186)
   defining Unicode char U+02C6 (decimal 710)
   defining Unicode char U+02DC (decimal 732)
   defining Unicode char U+200C (decimal 8204)
   defining Unicode char U+2026 (decimal 8230)
   defining Unicode char U+2122 (decimal 8482)
   defining Unicode char U+2423 (decimal 9251)
   defining Unicode char U+FEFF (decimal 65279)
)) (c:/TeXLive/2022/texmf-dist/tex/generic/babel/babel.sty
Package: babel 2023/02/13 3.86 The Babel package
\babel@savecnt=\count285
\U@D=\dimen154
\l@unhyphenated=\language87
```

```
(c:/TeXLive/2022/texmf-dist/tex/generic/babel/txtbabel.def)
\bbl@readstream=\read2
\bbl@dirlevel=\count286
(c:/TeXLive/2022/texmf-dist/tex/generic/babel-english/english.ldf
Language: english 2017/06/06 v3.3r English support from the babel system
Package babel Info: Hyphen rules for 'canadian' set to \l@english
(babel)             (\language0). Reported on input line 102.
Package babel Info: Hyphen rules for 'australian' set to \l@ukenglish
(babel)             (\language21). Reported on input line 105.
Package babel Info: Hyphen rules for 'newzealand' set to \l@ukenglish
(babel)             (\language21). Reported on input line 108.
)) (c:/TeXLive/2022/texmf-dist/tex/generic/babel/locale/en/babel-
english.tex
Package babel Info: Importing font and identification data for english
(babel)             from babel-en.ini. Reported on input line 11.
) (c:/TeXLive/2022/texmf-dist/tex/latex/lm/lmodern.sty
Package: lmodern 2015/05/01 v1.6.1 Latin Modern Fonts
LaTeX Font Info:    Overwriting symbol font `operators' in version
`normal'
(Font)                  OT1/ztmcm/m/n --> OT1/lmr/m/n on input line 22.
LaTeX Font Info:    Overwriting symbol font `letters' in version `normal'
(Font)                  OML/ztmcm/m/it --> OML/lmm/m/it on input line 23.
LaTeX Font Info:    Overwriting symbol font `symbols' in version `normal'
(Font)                  OMS/ztmcm/m/n --> OMS/lmsy/m/n on input line 24.
LaTeX Font Info:    Overwriting symbol font `largesymbols' in version
`normal'
(Font)                  OMX/ztmcm/m/n --> OMX/lmex/m/n on input line 25.
LaTeX Font Info:    Overwriting symbol font `operators' in version `bold'
(Font)                  OT1/ztmcm/m/n --> OT1/lmr/bx/n on input line 26.
LaTeX Font Info:    Overwriting symbol font `letters' in version `bold'
(Font)                  OML/ztmcm/m/it --> OML/lmm/b/it on input line 27.
LaTeX Font Info:    Overwriting symbol font `symbols' in version `bold'
(Font)                  OMS/ztmcm/m/n --> OMS/lmsy/b/n on input line 28.
LaTeX Font Info:    Overwriting symbol font `largesymbols' in version
`bold'
(Font)                  OMX/ztmcm/m/n --> OMX/lmex/m/n on input line 29.
LaTeX Font Info:    Overwriting math alphabet `\mathbf' in version
`normal'
(Font)                  OT1/ptm/bx/n --> OT1/lmr/bx/n on input line 31.
LaTeX Font Info:    Overwriting math alphabet `\mathsf' in version
`normal'
(Font)                  OT1/cmss/m/n --> OT1/lmss/m/n on input line 32.
LaTeX Font Info:    Overwriting math alphabet `\mathit' in version
`normal'
(Font)                  OT1/ptm/m/it --> OT1/lmr/m/it on input line 33.
LaTeX Font Info:    Overwriting math alphabet `\mathtt' in version
`normal'
(Font)                  OT1/cmtt/m/n --> OT1/lmtt/m/n on input line 34.
LaTeX Font Info:    Overwriting math alphabet `\mathbf' in version `bold'
(Font)                  OT1/ptm/bx/n --> OT1/lmr/bx/n on input line 35.
LaTeX Font Info:    Overwriting math alphabet `\mathsf' in version `bold'
(Font)                  OT1/cmss/bx/n --> OT1/lmss/bx/n on input line 36.
LaTeX Font Info:    Overwriting math alphabet `\mathit' in version `bold'
(Font)                  OT1/ptm/m/it --> OT1/lmr/bx/it on input line 37.
```

```
LaTeX Font Info:    Overwriting math alphabet `\mathtt' in version `bold'
(Font)                  OT1/cmtt/m/n --> OT1/lmtt/m/n on input line 38.
) (c:/TeXLive/2022/texmf-dist/tex/latex/siunitx/siunitx.sty
Package: siunitx 2023-03-04 v3.2.2 A comprehensive (SI) units package
\l__siunitx_angle_tmp_dim=\dimen155
\l__siunitx_angle_marker_box=\box53
\l__siunitx_angle_unit_box=\box54
\l__siunitx_compound_count_int=\count287
(c:/TeXLive/2022/texmf-dist/tex/latex/translations/translations.sty
Package: translations 2022/02/05 v1.12 internationalization of LaTeX2e
packages
 (CN)
(c:/TeXLive/2022/texmf-dist/tex/latex/etoolbox/etoolbox.sty
Package: etoolbox 2020/10/05 v2.5k e-TeX tools for LaTeX (JAW)
\etb@tempcnta=\count288
))
\l__siunitx_number_exponent_fixed_int=\count289
\l__siunitx_number_min_decimal_int=\count290
\l__siunitx_number_min_integer_int=\count291
\l__siunitx_number_round_precision_int=\count292
\l__siunitx_number_lower_threshold_int=\count293
\l__siunitx_number_upper_threshold_int=\count294
\l__siunitx_number_group_first_int=\count295
\l__siunitx_number_group_size_int=\count296
\l__siunitx_number_group_minimum_int=\count297
\l__siunitx_table_tmp_box=\box55
\l__siunitx_table_tmp_dim=\dimen156
\l__siunitx_table_column_width_dim=\dimen157
\l__siunitx_table_integer_box=\box56
\l__siunitx_table_decimal_box=\box57
\l__siunitx_table_uncert_box=\box58
\l__siunitx_table_before_box=\box59
\l__siunitx_table_after_box=\box60
\l__siunitx_table_before_dim=\dimen158
\l__siunitx_table_carry_dim=\dimen159
\l__siunitx_unit_tmp_int=\count298
\l__siunitx_unit_position_int=\count299
\l__siunitx_unit_total_int=\count300
(c:/TeXLive/2022/texmf-dist/tex/latex/tools/array.sty
Package: array 2022/09/04 v2.5g Tabular extension package (FMi)
\col@sep=\dimen160
\ar@mcellbox=\box61
\extrarowheight=\dimen161
\NC@list=\toks24
\extratabsurround=\skip56
\backup@length=\skip57
\ar@cellbox=\box62
)) (c:/TeXLive/2022/texmf-dist/tex/latex/textgreek/textgreek.sty
Package: textgreek 2011/10/09 v0.7 Greek symbols in text
Package textgreek Info: Loading the definitions for the Greek
fontencoding. on
input line 39.
(c:/TeXLive/2022/texmf-dist/tex/latex/greek-fontenc/lgrenc.def
File: lgrenc.def 2023/02/28 2.2 LGR Greek font encoding definitions
```

```
Now handling font encoding LGR ...
... processing UTF-8 mapping file for font encoding LGR
(c:/TeXLive/2022/texmf-dist/tex/latex/greek-inputenc/lgrenc.dfu
File: lgrenc.dfu 2023/02/21 1.8 UTF-8 support for Greek
   defining Unicode char U+00A8 (decimal 168)
   defining Unicode char U+00AB (decimal 171)
   defining Unicode char U+00AF (decimal 175)
   defining Unicode char U+00B4 (decimal 180)
   defining Unicode char U+00B7 (decimal 183)
   defining Unicode char U+00BB (decimal 187)
   defining Unicode char U+0259 (decimal 601)
   defining Unicode char U+02D8 (decimal 728)
   defining Unicode char U+0374 (decimal 884)
   defining Unicode char U+0375 (decimal 885)
   defining Unicode char U+037A (decimal 890)
   defining Unicode char U+037E (decimal 894)
   defining Unicode char U+0384 (decimal 900)
   defining Unicode char U+0385 (decimal 901)
   defining Unicode char U+0386 (decimal 902)
   defining Unicode char U+0387 (decimal 903)
   defining Unicode char U+0388 (decimal 904)
   defining Unicode char U+0389 (decimal 905)
   defining Unicode char U+038A (decimal 906)
   defining Unicode char U+038C (decimal 908)
   defining Unicode char U+038E (decimal 910)
   defining Unicode char U+038F (decimal 911)
   defining Unicode char U+0390 (decimal 912)
   defining Unicode char U+0391 (decimal 913)
   defining Unicode char U+0392 (decimal 914)
   defining Unicode char U+0393 (decimal 915)
   defining Unicode char U+0394 (decimal 916)
   defining Unicode char U+0395 (decimal 917)
   defining Unicode char U+0396 (decimal 918)
   defining Unicode char U+0397 (decimal 919)
   defining Unicode char U+0398 (decimal 920)
   defining Unicode char U+0399 (decimal 921)
   defining Unicode char U+039A (decimal 922)
   defining Unicode char U+039B (decimal 923)
   defining Unicode char U+039C (decimal 924)
   defining Unicode char U+039D (decimal 925)
   defining Unicode char U+039E (decimal 926)
   defining Unicode char U+039F (decimal 927)
   defining Unicode char U+03A0 (decimal 928)
   defining Unicode char U+03A1 (decimal 929)
   defining Unicode char U+03A3 (decimal 931)
   defining Unicode char U+03A4 (decimal 932)
   defining Unicode char U+03A5 (decimal 933)
   defining Unicode char U+03A6 (decimal 934)
   defining Unicode char U+03A7 (decimal 935)
   defining Unicode char U+03A8 (decimal 936)
   defining Unicode char U+03A9 (decimal 937)
   defining Unicode char U+03AA (decimal 938)
   defining Unicode char U+03AB (decimal 939)
   defining Unicode char U+03AC (decimal 940)
```

```
defining Unicode char U+03AD (decimal 941)
defining Unicode char U+03AE (decimal 942)
defining Unicode char U+03AF (decimal 943)
defining Unicode char U+03B0 (decimal 944)
defining Unicode char U+03B1 (decimal 945)
defining Unicode char U+03B2 (decimal 946)
defining Unicode char U+03B3 (decimal 947)
defining Unicode char U+03B4 (decimal 948)
defining Unicode char U+03B5 (decimal 949)
defining Unicode char U+03B6 (decimal 950)
defining Unicode char U+03B7 (decimal 951)
defining Unicode char U+03B8 (decimal 952)
defining Unicode char U+03B9 (decimal 953)
defining Unicode char U+03BA (decimal 954)
defining Unicode char U+03BB (decimal 955)
defining Unicode char U+03BC (decimal 956)
defining Unicode char U+03BD (decimal 957)
defining Unicode char U+03BE (decimal 958)
defining Unicode char U+03BF (decimal 959)
defining Unicode char U+03C0 (decimal 960)
defining Unicode char U+03C1 (decimal 961)
defining Unicode char U+03C2 (decimal 962)
defining Unicode char U+03C3 (decimal 963)
defining Unicode char U+03C4 (decimal 964)
defining Unicode char U+03C5 (decimal 965)
defining Unicode char U+03C6 (decimal 966)
defining Unicode char U+03C7 (decimal 967)
defining Unicode char U+03C8 (decimal 968)
defining Unicode char U+03C9 (decimal 969)
defining Unicode char U+03CA (decimal 970)
defining Unicode char U+03CB (decimal 971)
defining Unicode char U+03CC (decimal 972)
defining Unicode char U+03CD (decimal 973)
defining Unicode char U+03CE (decimal 974)
defining Unicode char U+03D0 (decimal 976)
defining Unicode char U+03D1 (decimal 977)
defining Unicode char U+03D5 (decimal 981)
defining Unicode char U+03D6 (decimal 982)
defining Unicode char U+03D8 (decimal 984)
defining Unicode char U+03D9 (decimal 985)
defining Unicode char U+03DA (decimal 986)
defining Unicode char U+03DB (decimal 987)
defining Unicode char U+03DC (decimal 988)
defining Unicode char U+03DD (decimal 989)
defining Unicode char U+03DE (decimal 990)
defining Unicode char U+03DF (decimal 991)
defining Unicode char U+03E0 (decimal 992)
defining Unicode char U+03E1 (decimal 993)
defining Unicode char U+03F0 (decimal 1008)
defining Unicode char U+03F1 (decimal 1009)
defining Unicode char U+03F4 (decimal 1012)
defining Unicode char U+03F5 (decimal 1013)
defining Unicode char U+1F00 (decimal 7936)
defining Unicode char U+1F01 (decimal 7937)
```

defining Unicode char U+1F02 (decimal 7938)
defining Unicode char U+1F03 (decimal 7939)
defining Unicode char U+1F04 (decimal 7940)
defining Unicode char U+1F05 (decimal 7941)
defining Unicode char U+1F06 (decimal 7942)
defining Unicode char U+1F07 (decimal 7943)
defining Unicode char U+1F08 (decimal 7944)
defining Unicode char U+1F09 (decimal 7945)
defining Unicode char U+1F0A (decimal 7946)
defining Unicode char U+1F0B (decimal 7947)
defining Unicode char U+1F0C (decimal 7948)
defining Unicode char U+1F0D (decimal 7949)
defining Unicode char U+1F0E (decimal 7950)
defining Unicode char U+1F0F (decimal 7951)
defining Unicode char U+1F10 (decimal 7952)
defining Unicode char U+1F11 (decimal 7953)
defining Unicode char U+1F12 (decimal 7954)
defining Unicode char U+1F13 (decimal 7955)
defining Unicode char U+1F14 (decimal 7956)
defining Unicode char U+1F15 (decimal 7957)
defining Unicode char U+1F18 (decimal 7960)
defining Unicode char U+1F19 (decimal 7961)
defining Unicode char U+1F1A (decimal 7962)
defining Unicode char U+1F1B (decimal 7963)
defining Unicode char U+1F1C (decimal 7964)
defining Unicode char U+1F1D (decimal 7965)
defining Unicode char U+1F20 (decimal 7968)
defining Unicode char U+1F21 (decimal 7969)
defining Unicode char U+1F22 (decimal 7970)
defining Unicode char U+1F23 (decimal 7971)
defining Unicode char U+1F24 (decimal 7972)
defining Unicode char U+1F25 (decimal 7973)
defining Unicode char U+1F26 (decimal 7974)
defining Unicode char U+1F27 (decimal 7975)
defining Unicode char U+1F28 (decimal 7976)
defining Unicode char U+1F29 (decimal 7977)
defining Unicode char U+1F2A (decimal 7978)
defining Unicode char U+1F2B (decimal 7979)
defining Unicode char U+1F2C (decimal 7980)
defining Unicode char U+1F2D (decimal 7981)
defining Unicode char U+1F2E (decimal 7982)
defining Unicode char U+1F2F (decimal 7983)
defining Unicode char U+1F30 (decimal 7984)
defining Unicode char U+1F31 (decimal 7985)
defining Unicode char U+1F32 (decimal 7986)
defining Unicode char U+1F33 (decimal 7987)
defining Unicode char U+1F34 (decimal 7988)
defining Unicode char U+1F35 (decimal 7989)
defining Unicode char U+1F36 (decimal 7990)
defining Unicode char U+1F37 (decimal 7991)
defining Unicode char U+1F38 (decimal 7992)
defining Unicode char U+1F39 (decimal 7993)
defining Unicode char U+1F3A (decimal 7994)
defining Unicode char U+1F3B (decimal 7995)

```
defining Unicode char U+1F3C (decimal 7996)
defining Unicode char U+1F3D (decimal 7997)
defining Unicode char U+1F3E (decimal 7998)
defining Unicode char U+1F3F (decimal 7999)
defining Unicode char U+1F40 (decimal 8000)
defining Unicode char U+1F41 (decimal 8001)
defining Unicode char U+1F42 (decimal 8002)
defining Unicode char U+1F43 (decimal 8003)
defining Unicode char U+1F44 (decimal 8004)
defining Unicode char U+1F45 (decimal 8005)
defining Unicode char U+1F48 (decimal 8008)
defining Unicode char U+1F49 (decimal 8009)
defining Unicode char U+1F4A (decimal 8010)
defining Unicode char U+1F4B (decimal 8011)
defining Unicode char U+1F4C (decimal 8012)
defining Unicode char U+1F4D (decimal 8013)
defining Unicode char U+1F50 (decimal 8016)
defining Unicode char U+1F51 (decimal 8017)
defining Unicode char U+1F52 (decimal 8018)
defining Unicode char U+1F53 (decimal 8019)
defining Unicode char U+1F54 (decimal 8020)
defining Unicode char U+1F55 (decimal 8021)
defining Unicode char U+1F56 (decimal 8022)
defining Unicode char U+1F57 (decimal 8023)
defining Unicode char U+1F59 (decimal 8025)
defining Unicode char U+1F5B (decimal 8027)
defining Unicode char U+1F5D (decimal 8029)
defining Unicode char U+1F5F (decimal 8031)
defining Unicode char U+1F60 (decimal 8032)
defining Unicode char U+1F61 (decimal 8033)
defining Unicode char U+1F62 (decimal 8034)
defining Unicode char U+1F63 (decimal 8035)
defining Unicode char U+1F64 (decimal 8036)
defining Unicode char U+1F65 (decimal 8037)
defining Unicode char U+1F66 (decimal 8038)
defining Unicode char U+1F67 (decimal 8039)
defining Unicode char U+1F68 (decimal 8040)
defining Unicode char U+1F69 (decimal 8041)
defining Unicode char U+1F6A (decimal 8042)
defining Unicode char U+1F6B (decimal 8043)
defining Unicode char U+1F6C (decimal 8044)
defining Unicode char U+1F6D (decimal 8045)
defining Unicode char U+1F6E (decimal 8046)
defining Unicode char U+1F6F (decimal 8047)
defining Unicode char U+1F70 (decimal 8048)
defining Unicode char U+1F71 (decimal 8049)
defining Unicode char U+1F72 (decimal 8050)
defining Unicode char U+1F73 (decimal 8051)
defining Unicode char U+1F74 (decimal 8052)
defining Unicode char U+1F75 (decimal 8053)
defining Unicode char U+1F76 (decimal 8054)
defining Unicode char U+1F77 (decimal 8055)
defining Unicode char U+1F78 (decimal 8056)
defining Unicode char U+1F79 (decimal 8057)
```

```
defining Unicode char U+1F7A (decimal 8058)
defining Unicode char U+1F7B (decimal 8059)
defining Unicode char U+1F7C (decimal 8060)
defining Unicode char U+1F7D (decimal 8061)
defining Unicode char U+1F80 (decimal 8064)
defining Unicode char U+1F81 (decimal 8065)
defining Unicode char U+1F82 (decimal 8066)
defining Unicode char U+1F83 (decimal 8067)
defining Unicode char U+1F84 (decimal 8068)
defining Unicode char U+1F85 (decimal 8069)
defining Unicode char U+1F86 (decimal 8070)
defining Unicode char U+1F87 (decimal 8071)
defining Unicode char U+1F88 (decimal 8072)
defining Unicode char U+1F89 (decimal 8073)
defining Unicode char U+1F8A (decimal 8074)
defining Unicode char U+1F8B (decimal 8075)
defining Unicode char U+1F8C (decimal 8076)
defining Unicode char U+1F8D (decimal 8077)
defining Unicode char U+1F8E (decimal 8078)
defining Unicode char U+1F8F (decimal 8079)
defining Unicode char U+1F90 (decimal 8080)
defining Unicode char U+1F91 (decimal 8081)
defining Unicode char U+1F92 (decimal 8082)
defining Unicode char U+1F93 (decimal 8083)
defining Unicode char U+1F94 (decimal 8084)
defining Unicode char U+1F95 (decimal 8085)
defining Unicode char U+1F96 (decimal 8086)
defining Unicode char U+1F97 (decimal 8087)
defining Unicode char U+1F98 (decimal 8088)
defining Unicode char U+1F99 (decimal 8089)
defining Unicode char U+1F9A (decimal 8090)
defining Unicode char U+1F9B (decimal 8091)
defining Unicode char U+1F9C (decimal 8092)
defining Unicode char U+1F9D (decimal 8093)
defining Unicode char U+1F9E (decimal 8094)
defining Unicode char U+1F9F (decimal 8095)
defining Unicode char U+1FA0 (decimal 8096)
defining Unicode char U+1FA1 (decimal 8097)
defining Unicode char U+1FA2 (decimal 8098)
defining Unicode char U+1FA3 (decimal 8099)
defining Unicode char U+1FA4 (decimal 8100)
defining Unicode char U+1FA5 (decimal 8101)
defining Unicode char U+1FA6 (decimal 8102)
defining Unicode char U+1FA7 (decimal 8103)
defining Unicode char U+1FA8 (decimal 8104)
defining Unicode char U+1FA9 (decimal 8105)
defining Unicode char U+1FAA (decimal 8106)
defining Unicode char U+1FAB (decimal 8107)
defining Unicode char U+1FAC (decimal 8108)
defining Unicode char U+1FAD (decimal 8109)
defining Unicode char U+1FAE (decimal 8110)
defining Unicode char U+1FAF (decimal 8111)
defining Unicode char U+1FB0 (decimal 8112)
defining Unicode char U+1FB1 (decimal 8113)
```

```
defining Unicode char U+1FB2 (decimal 8114)
defining Unicode char U+1FB3 (decimal 8115)
defining Unicode char U+1FB4 (decimal 8116)
defining Unicode char U+1FB6 (decimal 8118)
defining Unicode char U+1FB7 (decimal 8119)
defining Unicode char U+1FB8 (decimal 8120)
defining Unicode char U+1FB9 (decimal 8121)
defining Unicode char U+1FBA (decimal 8122)
defining Unicode char U+1FBB (decimal 8123)
defining Unicode char U+1FBC (decimal 8124)
defining Unicode char U+1FBD (decimal 8125)
defining Unicode char U+1FBE (decimal 8126)
defining Unicode char U+1FBF (decimal 8127)
defining Unicode char U+1FC0 (decimal 8128)
defining Unicode char U+1FC1 (decimal 8129)
defining Unicode char U+1FC2 (decimal 8130)
defining Unicode char U+1FC3 (decimal 8131)
defining Unicode char U+1FC4 (decimal 8132)
defining Unicode char U+1FC6 (decimal 8134)
defining Unicode char U+1FC7 (decimal 8135)
defining Unicode char U+1FC8 (decimal 8136)
defining Unicode char U+1FC9 (decimal 8137)
defining Unicode char U+1FCA (decimal 8138)
defining Unicode char U+1FCB (decimal 8139)
defining Unicode char U+1FCC (decimal 8140)
defining Unicode char U+1FCD (decimal 8141)
defining Unicode char U+1FCE (decimal 8142)
defining Unicode char U+1FCF (decimal 8143)
defining Unicode char U+1FD0 (decimal 8144)
defining Unicode char U+1FD1 (decimal 8145)
defining Unicode char U+1FD2 (decimal 8146)
defining Unicode char U+1FD3 (decimal 8147)
defining Unicode char U+1FD6 (decimal 8150)
defining Unicode char U+1FD7 (decimal 8151)
defining Unicode char U+1FD8 (decimal 8152)
defining Unicode char U+1FD9 (decimal 8153)
defining Unicode char U+1FDA (decimal 8154)
defining Unicode char U+1FDB (decimal 8155)
defining Unicode char U+1FDD (decimal 8157)
defining Unicode char U+1FDE (decimal 8158)
defining Unicode char U+1FDF (decimal 8159)
defining Unicode char U+1FE0 (decimal 8160)
defining Unicode char U+1FE1 (decimal 8161)
defining Unicode char U+1FE2 (decimal 8162)
defining Unicode char U+1FE3 (decimal 8163)
defining Unicode char U+1FE4 (decimal 8164)
defining Unicode char U+1FE5 (decimal 8165)
defining Unicode char U+1FE6 (decimal 8166)
defining Unicode char U+1FE7 (decimal 8167)
defining Unicode char U+1FE8 (decimal 8168)
defining Unicode char U+1FE9 (decimal 8169)
defining Unicode char U+1FEA (decimal 8170)
defining Unicode char U+1FEB (decimal 8171)
defining Unicode char U+1FEC (decimal 8172)
```

```
   defining Unicode char U+1FED (decimal 8173)
   defining Unicode char U+1FEE (decimal 8174)
   defining Unicode char U+1FEF (decimal 8175)
   defining Unicode char U+1FF2 (decimal 8178)
   defining Unicode char U+1FF3 (decimal 8179)
   defining Unicode char U+1FF4 (decimal 8180)
   defining Unicode char U+1FF6 (decimal 8182)
   defining Unicode char U+1FF7 (decimal 8183)
   defining Unicode char U+1FF8 (decimal 8184)
   defining Unicode char U+1FF9 (decimal 8185)
   defining Unicode char U+1FFA (decimal 8186)
   defining Unicode char U+1FFB (decimal 8187)
   defining Unicode char U+1FFC (decimal 8188)
   defining Unicode char U+1FFD (decimal 8189)
   defining Unicode char U+1FFE (decimal 8190)
   defining Unicode char U+2013 (decimal 8211)
   defining Unicode char U+2014 (decimal 8212)
   defining Unicode char U+2018 (decimal 8216)
   defining Unicode char U+2019 (decimal 8217)
   defining Unicode char U+201A (decimal 8218)
   defining Unicode char U+2030 (decimal 8240)
   defining Unicode char U+2039 (decimal 8249)
   defining Unicode char U+203A (decimal 8250)
   defining Unicode char U+20AC (decimal 8364)
   defining Unicode char U+2126 (decimal 8486)
   defining Unicode char U+10144 (decimal 65860)
   defining Unicode char U+10145 (decimal 65861)
   defining Unicode char U+10146 (decimal 65862)
   defining Unicode char U+10147 (decimal 65863)
) (c:/TeXLive/2022/texmf-dist/tex/latex/greek-fontenc/greek-fontenc.def
File: greek-fontenc.def 2023/03/08 2.2.1 Common Greek font encoding
definitions

))
Package textgreek Info: Loaded fontmap: {eur/*/*}{U/eur/*/*}
{phv/*/*}{U/psy/*/
*}. on input line 69.
) (c:/TeXLive/2022/texmf-dist/tex/latex/cleveref/cleveref.sty
Package: cleveref 2018/03/27 v0.21.4 Intelligent cross-referencing
Package cleveref Info: `hyperref' support loaded on input line 2370.
LaTeX Info: Redefining \cref on input line 2370.
LaTeX Info: Redefining \Cref on input line 2370.
LaTeX Info: Redefining \crefrange on input line 2370.
LaTeX Info: Redefining \Crefrange on input line 2370.
LaTeX Info: Redefining \cpageref on input line 2370.
LaTeX Info: Redefining \Cpageref on input line 2370.
LaTeX Info: Redefining \cpagerefrange on input line 2370.
LaTeX Info: Redefining \Cpagerefrange on input line 2370.
LaTeX Info: Redefining \labelcref on input line 2370.
LaTeX Info: Redefining \labelcpageref on input line 2370.
) (c:/TeXLive/2022/texmf-dist/tex/latex/gensymb/gensymb.sty
Package: gensymb 2022/10/17 v1.0.2 (KJH)
) (c:/TeXLive/2022/texmf-dist/tex/latex/base/textcomp.sty
Package: textcomp 2020/02/02 v2.0n Standard LaTeX package
```

```
) (c:/TeXLive/2022/texmf-dist/tex/latex/mhchem/mhchem.sty
Package: mhchem 2021/12/31 v4.09 for typesetting chemical formulae
(c:/TeXLive/2022/texmf-dist/tex/latex/l3kernel/expl3.sty
Package: expl3 2023-02-22 L3 programming layer (loader)

! LaTeX Error: Mismatched LaTeX support files detected.
(LaTeX)        Loading 'expl3.sty' aborted!
(LaTeX)
(LaTeX)        The L3 programming layer in the LaTeX format
(LaTeX)        is dated 2022-12-17, but in your TeX tree the files
require
(LaTeX)        at least 2023-02-22.

For immediate help type H <return>.
 ...

l.77      \ExplLoaderFileDate{expl3.sty}}
                                         %

The most likely causes are:
 - A recent format generation failed;
 - A stray format file in the user tree which needs to be removed or
rebuilt;
 - You are running a manually installed version of expl3.sty
   which is incompatible with the version in LaTeX.

LaTeX will abort loading the incompatible support files but this may lead
to
later errors. Please ensure that your LaTeX format is correctly
regenerated.


) (c:/TeXLive/2022/texmf-dist/tex/latex/l3packages/l3keys2e/l3keys2e.sty
Package: l3keys2e 2023-02-02 LaTeX2e option processing using LaTeX3 keys
) (c:/TeXLive/2022/texmf-dist/tex/latex/tools/calc.sty
Package: calc 2017/05/25 v4.3 Infix arithmetic (KKT,FJ)
\calc@Acount=\count301
\calc@Bcount=\count302
\calc@Adimen=\dimen162
\calc@Bdimen=\dimen163
\calc@Askip=\skip58
\calc@Bskip=\skip59
LaTeX Info: Redefining \setlength on input line 80.
LaTeX Info: Redefining \addtolength on input line 81.
\calc@Ccount=\count303
\calc@Cskip=\skip60
) (c:/TeXLive/2022/texmf-dist/tex/latex/chemgreek/chemgreek.sty
(c:/TeXLive/202
2/texmf-dist/tex/latex/l3packages/xparse/xparse.sty
Package: xparse 2023-02-02 L3 Experimental document command parser
)
Package: chemgreek 2020/01/16 v1.1a interface for upright Greek letters
for use
 in chemistry (CN)
```

```
\l__chemgreek_tmpa_int=\count304
\g__chemgreek_tmpa_int=\count305
) (c:/TeXLive/2022/texmf-dist/tex/latex/graphics/graphics.sty
Package: graphics 2022/03/10 v1.4e Standard LaTeX Graphics (DPC,SPQR)
(c:/TeXLive/2022/texmf-dist/tex/latex/graphics/trig.sty
Package: trig 2021/08/11 v1.11 sin cos tan (DPC)
) (c:/TeXLive/2022/texmf-dist/tex/latex/graphics-cfg/graphics.cfg
File: graphics.cfg 2016/06/04 v1.11 sample graphics configuration
)
Package graphics Info: Driver file: pdftex.def on input line 107.
)
\l__mhchem_ce_distanceFromLastComma_int=\count306
\__mhchem_arrow_extPgfLength_skip=\skip61
\mhchem@minispace@tmp=\skip62
\mhchem@minibackspace@tmp=\skip63
\mhchem@bondwidth=\skip64
\mhchem@bondheight=\skip65
\mhchem@smallbondwidth@tmpA=\skip66
\mhchem@smallbondwidth@tmpB=\skip67
\mhchem@smallbondwidth=\skip68
\mhchem@bondtmp@minussidebearingleft=\skip69
\mhchem@bondtmp@minussidebearingright=\skip70
\l__mhchem_option_version_int=\count307
\mhchem@option@minusmathsidebearingleft=\dimen164
\mhchem@option@minusmathsidebearingright=\dimen165
\mhchem@option@minustextsidebearingleft=\dimen166
\mhchem@option@minustextsidebearingright=\dimen167
) (c:/TeXLive/2022/texmf-dist/tex/latex/psnfss/helvet.sty
Package: helvet 2020/03/25 PSNFSS-v9.3 (WaS)
) (c:/TeXLive/2022/texmf-dist/tex/latex/base/fontenc.sty
Package: fontenc 2021/04/29 v2.0v Standard LaTeX package
LaTeX Font Info:    Trying to load font information for T1+lmr on input line 11
2.
(c:/TeXLive/2022/texmf-dist/tex/latex/lm/t1lmr.fd
File: t1lmr.fd 2015/05/01 v1.6.1 Font defs for Latin Modern
)) (c:/TeXLive/2022/texmf-dist/tex/latex/lettrine/lettrine.sty
File: lettrine.sty 2022-09-25 v2.30 (Daniel Flipo)
(c:/TeXLive/2022/texmf-dist/tex/latex/xkeyval/xkeyval.sty
Package: xkeyval 2022/06/16 v2.9 package option processing (HA)
(c:/TeXLive/2022/texmf-dist/tex/generic/xkeyval/xkeyval.tex
(c:/TeXLive/2022/te
xmf-dist/tex/generic/xkeyval/xkvutils.tex
\XKV@toks=\toks25
\XKV@tempa@toks=\toks26
)
\XKV@depth=\count308
File: xkeyval.tex 2014/12/03 v2.7a key=value parser (HA)
)) (c:/TeXLive/2022/texmf-dist/tex/generic/minifp/minifp.sty
Package: minifp 2013/12/30 v0.96. Macros for real number operations and a stack
-based programing language.
\MFP@loopctr=\count309
)
```

```
\c@DefaultLines=\count310
\c@DefaultDepth=\count311
\DefaultFindent=\dimen168
\DefaultNindent=\dimen169
\DefaultSlope=\dimen170
\DiscardVskip=\dimen171
Loading lettrine.cfg
(c:/TeXLive/2022/texmf-dist/tex/latex/lettrine/lettrine.cfg)
\L@lbox=\box63
\L@tbox=\box64
\c@L@lines=\count312
\c@L@depth=\count313
\L@Pindent=\dimen172
\L@Findent=\dimen173
\L@Nindent=\dimen174
\L@lraise=\dimen175
\L@first=\dimen176
\L@next=\dimen177
\L@slope=\dimen178
\L@height=\dimen179
\L@novskip=\dimen180
\L@target@ht=\dimen181
\L@target@dp=\dimen182
\L@target@tht=\dimen183
\LettrineWidth=\dimen184
\LettrineHeight=\dimen185
\LettrineDepth=\dimen186
\Llist@everypar=\toks27
) (c:/TeXLive/2022/texmf-dist/tex/latex/sidecap/sidecap.sty
Package: sidecap 2023/01/24 v1.7a SideCap Package (RN/HjG)
(c:/TeXLive/2022/texmf-dist/tex/latex/base/ifthen.sty
Package: ifthen 2022/04/13 v1.1d Standard LaTeX ifthen package (DPC)
)
\SC@BOXWD=\dimen187
\SC@CAPWD=\dimen188
\SC@tempdima=\dimen189
\SC@tempdimb=\dimen190
\c@SC@C=\count314
\SC@BOX=\box65
) (c:/TeXLive/2022/texmf-dist/tex/latex/ifsym/ifsym.sty
Package: ifsym 2000/04/18 I.Kloeckl
\c@ifsymcnt=\count315
) (c:/TeXLive/2022/texmf-dist/tex/latex/bbding/bbding.sty
Package: bbding 1999/04/15 v1.01 Dingbats symbols
) (c:/TeXLive/2022/texmf-dist/tex/latex/geometry/geometry.sty
Package: geometry 2020/01/02 v5.9 Page Geometry
(c:/TeXLive/2022/texmf-dist/tex/generic/iftex/ifvtex.sty
Package: ifvtex 2019/10/25 v1.7 ifvtex legacy package. Use iftex instead.
)
\Gm@cnth=\count316
\Gm@cntv=\count317
\c@Gm@tempcnt=\count318
\Gm@bindingoffset=\dimen191
\Gm@wd@mp=\dimen192
```

```
\Gm@odd@mp=\dimen193
\Gm@even@mp=\dimen194
\Gm@layoutwidth=\dimen195
\Gm@layoutheight=\dimen196
\Gm@layouthoffset=\dimen197
\Gm@layoutvoffset=\dimen198
\Gm@dimlist=\toks28
) (c:/TeXLive/2022/texmf-dist/tex/latex/caption/caption.sty
Package: caption 2022/03/01 v3.6b Customizing captions (AR)
(c:/TeXLive/2022/texmf-dist/tex/latex/caption/caption3.sty
Package: caption3 2022/03/17 v2.3b caption3 kernel (AR)
\caption@tempdima=\dimen199
\captionmargin=\dimen256
\caption@leftmargin=\dimen257
\caption@rightmargin=\dimen258
\caption@width=\dimen259
\caption@indent=\dimen260
\caption@parindent=\dimen261
\caption@hangindent=\dimen262
Package caption Info: Standard document class detected.
)
Package caption Info: Option `figurename' requires the `newfloat' package
on in
put line 169.
(c:/TeXLive/2022/texmf-dist/tex/latex/newfloat/newfloat.sty
Package: newfloat 2019/09/02 v1.1l Defining new floating environments
(AR)
Package newfloat Info: Unsupported document class, or \@chapter
(newfloat)             was already redefined by another package.
Package newfloat Info: \@chapter = macro:->\def \Hy@next
{\Hy@MakeCurrentHrefAu
to {\Hy@chapapp *}\Hy@raisedlink {\hyper@anchorstart {\@currentHref
}\hyper@anc
horend }}\ifnum \c@secnumdepth >\m@ne \ltx@IfUndefined
{if@mainmatter}\iftrue {
\csname if@mainmatter\endcsname }\let \Hy@next \relax \fi \fi \Hy@next
\Hy@org@
chapter .
Package newfloat Info: \Hy@org@chapter = macro:[#1]#2->\NR@gettitle
{#1}\NR@cha
pter [{#1}]{#2}.
Package newfloat Info: `sidecap' package detected.
)
\c@caption@flags=\count319
\c@continuedfloat=\count320
Package caption Info: hyperref package is loaded.
Package caption Info: sidecap package is loaded.
)
   defining Unicode char U+00B0 (decimal 176)
(c:/TeXLive/2022/texmf-dist/tex/latex/psnfss/times.sty
Package: times 2020/03/25 PSNFSS-v9.3 (SPQR)
)
\@maxsep=\dimen263
\@dblmaxsep=\dimen264
```

```
(c:/TeXLive/2022/texmf-dist/tex/latex/titlesec/titlesec.sty
Package: titlesec 2021/07/05 v2.14 Sectioning titles
\ttl@box=\box66
\beforetitleunit=\skip71
\aftertitleunit=\skip72
\ttl@plus=\dimen265
\ttl@minus=\dimen266
\ttl@toksa=\toks29
\titlewidth=\dimen267
\titlewidthlast=\dimen268
\titlewidthfirst=\dimen269
)
\c@ttlp@side=\count321
\ttlp@side=\count322
(c:/TeXLive/2022/texmf-dist/tex/latex/xwatermark/xwatermark.sty
(c:/TeXLive/202
2/texmf-dist/tex/latex/catoptions/catoptions.sty
\m@one=\count323
\z@pt=\dimen270
Package: catoptions 2014/08/29 v0.2.7h Securing catcodes and parsing
options (A
M)
\cpt@csvdepth=\count324
\cpt@optdepth=\count325
\cpt@cnta=\count326
\indrisdepth=\count327
\cpt@foreachdepth=\count328
\cpt@exprcnt=\count329
)
Package: xwatermark 2012/10/23 v1.5.2d A dynamic watermarking scheme
(Ahmed Mus
a)
(c:/TeXLive/2022/texmf-dist/tex/latex/base/fix-cm.sty
Package: fix-cm 2020/11/24 v1.1t fixes to LaTeX
(c:/TeXLive/2022/texmf-dist/tex/latex/base/ts1enc.def
File: ts1enc.def 2001/06/05 v3.0e (jk/car/fm) Standard LaTeX file
LaTeX Font Info:    Redeclaring font encoding TS1 on input line 47.
)) (c:/TeXLive/2022/texmf-dist/tex/latex/picture/picture.sty
Package: picture 2020-04-22 v1.6 Dimens for picture macros (HO)
Package picture Info: Calculation method: calc.
) (c:/TeXLive/2022/texmf-dist/tex/latex/graphics/graphicx.sty
Package: graphicx 2021/09/16 v1.2d Enhanced LaTeX Graphics (DPC,SPQR)
\Gin@req@height=\dimen271
\Gin@req@width=\dimen272
) (c:/TeXLive/2022/texmf-dist/tex/latex/fancyhdr/fancyhdr.sty
Package: fancyhdr 2022/11/09 v4.1 Extensive control of page headers and
footers

\f@nch@headwidth=\skip73
\f@nch@O@elh=\skip74
\f@nch@O@erh=\skip75
\f@nch@O@olh=\skip76
\f@nch@O@orh=\skip77
\f@nch@O@elf=\skip78
```

```
\f@nch@O@erf=\skip79
\f@nch@O@olf=\skip80
\f@nch@O@orf=\skip81
) (c:/TeXLive/2022/texmf-dist/tex/latex/ltxkeys/ltxkeys.sty
Package: ltxkeys 2012/11/17 v0.0.3c A robust key-value parser (AM) (Ahmed
Musa)

\ltxkeys@csvdepth=\count330
\ltxkeys@keydepth=\count331
) (c:/TeXLive/2022/texmf-dist/tex/latex/framed/framed.sty
Package: framed 2011/10/22 v 0.96: framed or shaded text with page breaks
\OuterFrameSep=\skip82
\fb@frw=\dimen273
\fb@frh=\dimen274
\FrameRule=\dimen275
\FrameSep=\dimen276
)
\xwm@boxa=\box67
\xwm@boxb=\box68
\xwm@cnta=\count332
\xwm@wrt=\write3
(./main.xwm)
\openout3 = `main.xwm'.

) (c:/TeXLive/2022/texmf-dist/tex/latex/tools/verbatim.sty
Package: verbatim 2022-07-02 v1.5u LaTeX2e package for verbatim
enhancements
\every@verbatim=\toks30
\verbatim@line=\toks31
\verbatim@in@stream=\read3
) (c:/TeXLive/2022/texmf-dist/tex/latex/cite/chapterbib.sty
\c@inputfile=\count333
Package: chapterbib 2010/09/18  v 1.17
) (c:/TeXLive/2022/texmf-dist/tex/latex/natbib/natbib.sty
Package: natbib 2010/09/13 8.31b (PWD, AO)
\bibhang=\skip83
\bibsep=\skip84
LaTeX Info: Redefining \cite on input line 694.
\c@NAT@ctr=\count334
)
\tmpitemindent=\dimen277
\c@theorem=\count335
\c@definition=\count336
\c@corollary=\count337
(c:/TeXLive/2022/texmf-dist/tex/latex/lastpage/lastpage.sty
Package: lastpage 2023/03/07 v2.0a lastpage: 2.09 or 2e? (HMM)
(c:/TeXLive/2022/texmf-dist/tex/latex/lastpage/lastpage2e.sty
Package: lastpage2e 2023/03/07 v2.0a Decide which 2e lastpage version to
use (H
MM)
(c:/TeXLive/2022/texmf-dist/tex/latex/lastpage/lastpagemodern.sty
Package: lastpagemodern 2023-03-07 v2.0a Refers to last page's name (HMM;
JPG)
)
```

```
))) (c:/TeXLive/2022/texmf-dist/tex/latex/blindtext/blindtext.sty
Package: blindtext 2012/01/06 V2.0 blindtext-Package
(c:/TeXLive/2022/texmf-dist/tex/latex/tools/xspace.sty
Package: xspace 2014/10/28 v1.13 Space after command names (DPC,MH)
)
\c@blindtext=\count338
\c@Blindtext=\count339
\c@blind@countparstart=\count340
\blind@countxx=\count341
\blindtext@numBlindtext=\count342
\blind@countyy=\count343
\c@blindlist=\count344
\c@blindlistlevel=\count345
\c@blindlist@level=\count346
\blind@listitem=\count347
\c@blind@listcount=\count348
\c@blind@levelcount=\count349
\blind@mathformula=\count350
\blind@Mathformula=\count351
\c@blind@randomcount=\count352
\c@blind@randommax=\count353
\c@blind@pangramcount=\count354
\c@blind@pangrammax=\count355
) (c:/TeXLive/2022/texmf-dist/tex/latex/sttools/flushend.sty
Package: flushend 2021/10/04 v4.0 Balancing columns in twocolumn mode
\flushend@@lastskip@a=\skip85
\flushend@@lastskip@b=\skip86
\flushend@@lastnode=\count356
\var@@loop@iter=\count357
\var@@temp@spread=\dimen278
\var@@temp@a=\dimen279
\var@@temp@loop=\dimen280
\flushend@@page@rule=\dimen281
\flushend@@varbox@lastpage=\box69
\flushend@@varbox@a=\box70
\flushend@@varbox@c=\box71
\flushend@@tempbox@a=\box72
\flushend@@tempbox@c=\box73
\flushend@@floatbox=\box74
\@viper=\box75
\hold@viper=\box76
\atColsBreak=\toks32
\atColsEnd=\toks33
\oldbreak@skip=\dimen282
) (c:/TeXLive/2022/texmf-dist/tex/latex/booktabs/booktabs.sty
Package: booktabs 2020/01/12 v1.61803398 Publication quality tables
\heavyrulewidth=\dimen283
\lightrulewidth=\dimen284
\cmidrulewidth=\dimen285
\belowrulesep=\dimen286
\belowbottomsep=\dimen287
\aboverulesep=\dimen288
\abovetopsep=\dimen289
\cmidrulesep=\dimen290
```

```
\cmidrulekern=\dimen291
\defaultaddspace=\dimen292
\@cmidla=\count358
\@cmidlb=\count359
\@aboverulesep=\dimen293
\@belowrulesep=\dimen294
\@thisruleclass=\count360
\@lastruleclass=\count361
\@thisrulewidth=\dimen295
) (c:/TeXLive/2022/texmf-dist/tex/latex/multirow/multirow.sty
Package: multirow 2021/03/15 v2.8 Span multiple rows of a table
\multirow@colwidth=\skip87
\multirow@cntb=\count362
\multirow@dima=\skip88
\bigstrutjot=\dimen296
) (c:/TeXLive/2022/texmf-dist/tex/latex/tools/tabularx.sty
Package: tabularx 2020/01/15 v2.11c `tabularx' package (DPC)
\TX@col@width=\dimen297
\TX@old@table=\dimen298
\TX@old@col=\dimen299
\TX@target=\dimen300
\TX@delta=\dimen301
\TX@cols=\count363
\TX@ftn=\toks34
)
Package hyperref Info: Option `draft' set `true' on input line 14.
! Extra \endgroup.
\document ->\endgroup
                      \let \BeforeStartOfDocument \@firstofone
\cpt@beforest...
l.15 \begin{document}

Things are pretty mixed up, but I think the worst is over.

LaTeX Font Info:    Trying to load font information for T1+ptm on input
line 15
.
(c:/TeXLive/2022/texmf-dist/tex/latex/psnfss/t1ptm.fd
File: t1ptm.fd 2001/06/04 font definitions for T1/ptm.
) (c:/TeXLive/2022/texmf-dist/tex/latex/l3backend/l3backend-pdftex.def
File: l3backend-pdftex.def 2023-01-16 L3 backend support: PDF output
(pdfTeX)
\l__color_backend_stack_int=\count364
\l__pdf_internal_box=\box77
) (./main.aux)
\openout1 = `main.aux'.

LaTeX Font Info:    Checking defaults for OML/cmm/m/it on input line 15.
LaTeX Font Info:    ... okay on input line 15.
LaTeX Font Info:    Checking defaults for OMS/cmsy/m/n on input line 15.
LaTeX Font Info:    ... okay on input line 15.
LaTeX Font Info:    Checking defaults for OT1/cmr/m/n on input line 15.
LaTeX Font Info:    ... okay on input line 15.
LaTeX Font Info:    Checking defaults for T1/cmr/m/n on input line 15.
```

```
LaTeX Font Info:    ... okay on input line 15.
LaTeX Font Info:    Checking defaults for TS1/cmr/m/n on input line 15.
LaTeX Font Info:    ... okay on input line 15.
LaTeX Font Info:    Checking defaults for OMX/cmex/m/n on input line 15.
LaTeX Font Info:    ... okay on input line 15.
LaTeX Font Info:    Checking defaults for U/cmr/m/n on input line 15.
LaTeX Font Info:    ... okay on input line 15.
LaTeX Font Info:    Checking defaults for PD1/pdf/m/n on input line 15.
LaTeX Font Info:    ... okay on input line 15.
LaTeX Font Info:    Checking defaults for PU/pdf/m/n on input line 15.
LaTeX Font Info:    ... okay on input line 15.
LaTeX Font Info:    Checking defaults for LGR/cmr/m/n on input line 15.
LaTeX Font Info:    Trying to load font information for LGR+cmr on input
line 1
5.
(c:/TeXLive/2022/texmf-dist/tex/latex/cbfonts-fd/lgrcmr.fd
File: lgrcmr.fd 2017/07/29 v1.2 Greek European Computer Regular
)
LaTeX Font Info:    ... okay on input line 15.

Package hyperref Warning: Draft mode on.

Package hyperref Info: Link coloring ON on input line 15.
(c:/TeXLive/2022/texmf-dist/tex/context/base/mkii/supp-pdf.mkii
[Loading MPS to PDF converter (version 2006.09.02).]
\scratchcounter=\count365
\scratchdimen=\dimen302
\scratchbox=\box78
\nofMPsegments=\count366
\nofMParguments=\count367
\everyMPshowfont=\toks35
\MPscratchCnt=\count368
\MPscratchDim=\dimen303
\MPnumerator=\count369
\makeMPintoPDFobject=\count370
\everyMPtoPDFconversion=\toks36
) (c:/TeXLive/2022/texmf-dist/tex/latex/epstopdf-pkg/epstopdf-base.sty
Package: epstopdf-base 2020-01-24 v2.11 Base part for package epstopdf
Package epstopdf-base Info: Redefining graphics rule for `.eps' on input
line 4
85.
(c:/TeXLive/2022/texmf-dist/tex/latex/latexconfig/epstopdf-sys.cfg
File: epstopdf-sys.cfg 2010/07/13 v1.3 Configuration of (r)epstopdf for
TeX Liv
e
))
(c:/TeXLive/2022/texmf-dist/tex/latex/translations/translations-basic-
dictionar
y-english.trsl
File: translations-basic-dictionary-english.trsl (english translation
file `tra
nslations-basic-dictionary')
)
```

```
Package translations Info: loading dictionary `translations-basic-
dictionary' f
or `english'. on input line 15.
\symgns@font=\mathgroup8
LaTeX Font Info:    Overwriting symbol font `gns@font' in version `bold'
(Font)                  TS1/ptm/m/n --> TS1/ptm/b/n on input line 15.
Package gensymb Info: Math companion symbols declared on input line 15.
LaTeX Info: Redefining \degree on input line 15.
LaTeX Info: Redefining \celsius on input line 15.
Package gensymb Info: Using text companion symbols for \degree, \celsius
and \p
erthousand on input line 15.
LaTeX Info: Redefining \ohm on input line 15.
Package gensymb Info: Using \textohm for \ohm on input line 15.
LaTeX Info: Redefining \micro on input line 15.
Package gensymb Info: Using \textmu for \micro on input line 15.

Package chemgreek Info: Activating mapping `textgreek' on line 15.

(c:/TeXLive/2022/texmf-dist/tex/latex/ragged2e/ragged2e.sty
Package: ragged2e 2023/02/25 v3.4 ragged2e Package
\CenteringLeftskip=\skip89
\RaggedLeftLeftskip=\skip90
\RaggedRightLeftskip=\skip91
\CenteringRightskip=\skip92
\RaggedLeftRightskip=\skip93
\RaggedRightRightskip=\skip94
\CenteringParfillskip=\skip95
\RaggedLeftParfillskip=\skip96
\RaggedRightParfillskip=\skip97
\JustifyingParfillskip=\skip98
\CenteringParindent=\skip99
\RaggedLeftParindent=\skip100
\RaggedRightParindent=\skip101
\JustifyingParindent=\skip102
)
*geometry* driver: auto-detecting
*geometry* detected driver: pdftex
*geometry* verbose mode - [ preamble ] result:
* driver: pdftex
* paper: letterpaper
* layout: <custom>
* layout(width,height): (587.19374pt,785.93625pt)
* layoutoffset:(h,v)=(13.55061pt,4.51686pt)
* modes: includehead includefoot twoside
* h-part:(L,W,R)=(38.5pt, 505.69374pt, 43.0pt)
* v-part:(T,H,B)=(43.0pt, 710.93625pt, 32.0pt)
* \paperwidth=614.295pt
* \paperheight=794.96999pt
* \textwidth=505.69374pt
* \textheight=691.40196pt
* \oddsidemargin=-20.21938pt
* \evensidemargin=-15.71938pt
* \topmargin=-45.5244pt
```

```
* \headheight=12.0pt
* \headsep=17.92523pt
* \topskip=10.0pt
* \footskip=28.45274pt
* \marginparwidth=20.0pt
* \marginparsep=10.0pt
* \columnsep=19.91692pt
* \skip\footins=10.0pt plus 4.0pt minus 2.0pt
* \hoffset=0.0pt
* \voffset=0.0pt
* \mag=1000
* \@twocolumntrue
* \@twosidetrue
* \@mparswitchtrue
* \@reversemarginfalse
* (1in=72.27pt=25.4mm, 1cm=28.453pt)

Package caption Info: Begin \AtBeginDocument code.
Package caption Info: End \AtBeginDocument code.
Package newfloat Info: Trying Plan B...
LaTeX Font Info:    Trying to load font information for T1+phv on input
line 30
.
(c:/TeXLive/2022/texmf-dist/tex/latex/psnfss/t1phv.fd
File: t1phv.fd 2020/03/25 scalable font definitions for T1/phv.
)
LaTeX Font Info:    Font shape `T1/phv/m/n' will be
(Font)              scaled to size 11.39996pt on input line 30.
LaTeX Font Info:    Font shape `T1/phv/b/n' will be
(Font)              scaled to size 11.39996pt on input line 30.
LaTeX Font Info:    Font shape `T1/phv/b/n' will be
(Font)              scaled to size 8.54997pt on input line 30.
LaTeX Font Info:    Trying to load font information for OT1+lmr on input
line 3
0.
(c:/TeXLive/2022/texmf-dist/tex/latex/lm/ot1lmr.fd
File: ot1lmr.fd 2015/05/01 v1.6.1 Font defs for Latin Modern
)
LaTeX Font Info:    Trying to load font information for OML+lmm on input
line 3
0.
(c:/TeXLive/2022/texmf-dist/tex/latex/lm/omllmm.fd
File: omllmm.fd 2015/05/01 v1.6.1 Font defs for Latin Modern
)
LaTeX Font Info:    Trying to load font information for OMS+lmsy on input
line
30.
(c:/TeXLive/2022/texmf-dist/tex/latex/lm/omslmsy.fd
File: omslmsy.fd 2015/05/01 v1.6.1 Font defs for Latin Modern
)
LaTeX Font Info:    Trying to load font information for OMX+lmex on input
line
30.
(c:/TeXLive/2022/texmf-dist/tex/latex/lm/omxlmex.fd
```

```
File: omxlmex.fd 2015/05/01 v1.6.1 Font defs for Latin Modern
)
LaTeX Font Info:    External font `lmex10' loaded for size
(Font)              <9> on input line 30.
LaTeX Font Info:    External font `lmex10' loaded for size
(Font)              <7> on input line 30.
LaTeX Font Info:    External font `lmex10' loaded for size
(Font)              <5> on input line 30.
LaTeX Font Info:    Trying to load font information for OT1+ptm on input
line 3
0.
(c:/TeXLive/2022/texmf-dist/tex/latex/psnfss/ot1ptm.fd
File: ot1ptm.fd 2001/06/04 font definitions for OT1/ptm.
)
LaTeX Font Info:    Font shape `OT1/ptm/bx/n' in size <9> not available
(Font)              Font shape `OT1/ptm/b/n' tried instead on input line
30.
LaTeX Font Info:    Font shape `OT1/ptm/bx/n' in size <7> not available
(Font)              Font shape `OT1/ptm/b/n' tried instead on input line
30.
LaTeX Font Info:    Font shape `OT1/ptm/bx/n' in size <5> not available
(Font)              Font shape `OT1/ptm/b/n' tried instead on input line
30.
LaTeX Font Info:    Trying to load font information for TS1+ptm on input
line 3
0.
(c:/TeXLive/2022/texmf-dist/tex/latex/psnfss/ts1ptm.fd
File: ts1ptm.fd 2001/06/04 font definitions for TS1/ptm.
)
LaTeX Font Info:    Font shape `T1/phv/b/n' will be
(Font)              scaled to size 6.64998pt on input line 30.
LaTeX Font Info:    Font shape `T1/phv/m/n' will be
(Font)              scaled to size 8.54997pt on input line 30.
LaTeX Font Info:    Trying to load font information for U+ifsym on input
line 3
0.
(c:/TeXLive/2022/texmf-dist/tex/latex/ifsym/uifsym.fd)
LaTeX Font Info:    Font shape `T1/phv/m/n' will be
(Font)              scaled to size 6.64998pt on input line 30.
LaTeX Font Info:    Font shape `T1/phv/m/n' will be
(Font)              scaled to size 4.74998pt on input line 30.
LaTeX Font Info:    Font shape `T1/phv/m/n' will be
(Font)              scaled to size 9.49997pt on input line 54.
LaTeX Font Info:    Font shape `T1/phv/b/n' will be
(Font)              scaled to size 9.49997pt on input line 56.
LaTeX Font Info:    Font shape `T1/phv/b/it' in size <7> not available
(Font)              Font shape `T1/phv/b/sl' tried instead on input line
58.
LaTeX Font Info:    Font shape `T1/phv/b/sl' will be
(Font)              scaled to size 6.64998pt on input line 58.
LaTeX Font Info:    Font shape `T1/phv/b/it' in size <10> not available
(Font)              Font shape `T1/phv/b/sl' tried instead on input line
60.
LaTeX Font Info:    Font shape `T1/phv/b/sl' will be
```

```
(Font)              scaled to size 9.49997pt on input line 60.

Package natbib Warning: Citation `stephens_big_2015' on page 1 undefined
on inp
ut line 64.


Package natbib Warning: Citation `stein_case_2010' on page 1 undefined on
input
 line 65.


Package natbib Warning: Citation `goodwin_coming_2016' on page 1
undefined on i
nput line 65.


Package natbib Warning: Citation `prins_toward_2015' on page 1 undefined
on inp
ut line 66.


Package natbib Warning: Citation `perkel_workflow_2019' on page 1
undefined on
input line 67.


Package natbib Warning: Citation `banati_evaluating_2016' on page 1
undefined o
n input line 68.


Package natbib Warning: Citation `noauthor_software_2014' on page 1
undefined o
n input line 70.


Package natbib Warning: Citation `noauthor_rebooting_2015' on page 1
undefined
on input line 70.


Package natbib Warning: Citation `baker_1500_2016' on page 1 undefined on
input
 line 71.


Package natbib Warning: Citation `drummond_replicability_2009' on page 1
undefi
ned on input line 73.


Package natbib Warning: Citation `acm_artifact_2021' on page 1 undefined
on inp
```

```
ut line 74.


Package natbib Warning: Citation `acm_artifact_2021' on page 1 undefined
on inp
ut line 85.


Package natbib Warning: Citation `acm_artifact_2021' on page 1 undefined
on inp
ut line 85.

LaTeX Font Info:    External font `lmex10' loaded for size
(Font)              <10> on input line 87.
LaTeX Font Info:    External font `lmex10' loaded for size
(Font)              <7.4> on input line 87.
LaTeX Font Info:    External font `lmex10' loaded for size
(Font)              <6> on input line 87.
LaTeX Font Info:    Font shape `OT1/ptm/bx/n' in size <10> not available
(Font)              Font shape `OT1/ptm/b/n' tried instead on input line
87.
LaTeX Font Info:    Font shape `OT1/ptm/bx/n' in size <7.4> not available
(Font)              Font shape `OT1/ptm/b/n' tried instead on input line
87.
LaTeX Font Info:    Font shape `OT1/ptm/bx/n' in size <6> not available
(Font)              Font shape `OT1/ptm/b/n' tried instead on input line
87.

Package natbib Warning: Citation `leprevost_best_2014' on page 1
undefined on i
nput line 98.


Package natbib Warning: Citation `wratten_reproducible_2021' on page 1
undefine
d on input line 98.


Package natbib Warning: Citation `amstutz_existing_2021' on page 1
undefined on
 input line 98.


Package natbib Warning: Citation `crusoe_methods_2022' on page 1
undefined on i
nput line 101.


Package natbib Warning: Citation `voss_full-stack_2017' on page 1
undefined on
input line 101.
```

```
Package natbib Warning: Citation `di_tommaso_nextflow_2017' on page 1
undefined
 on input line 101.


Package natbib Warning: Citation `koster_snakemake_2012' on page 1
undefined on
 input line 101.


Package natbib Warning: Citation `leprevost_biocontainers_2017' on page 1
undef
ined on input line 103.


Underfull \vbox (badness 4279) has occurred while \output is active []

LaTeX Font Info:    Font shape `T1/phv/m/n' will be
(Font)              scaled to size 7.59998pt on input line 105.
LaTeX Font Info:    Font shape `T1/phv/m/it' in size <7> not available
(Font)              Font shape `T1/phv/m/sl' tried instead on input line
105.
LaTeX Font Info:    Font shape `T1/phv/m/sl' will be
(Font)              scaled to size 6.64998pt on input line 105.
LaTeX Font Info:    Trying to load font information for T1+bch on input
line 10
5.
(c:/TeXLive/2022/texmf-dist/tex/latex/psnfss/t1bch.fd
File: t1bch.fd 2004/10/18 font definitions for T1/bch.
) [1{c:/TeXLive/2022/texmf-var/fonts/map/pdftex/updmap/pdftex.map}



]

Package natbib Warning: Citation `soiland-reyes_packaging_2022' on page 2
undef
ined on input line 107.


Package natbib Warning: Citation `khan_sharing_2019' on page 2 undefined
on inp
ut line 107.


Package natbib Warning: Citation `goble_implementing_2021' on page 2
undefined
on input line 108.


Package natbib Warning: Citation `oconnor_dockstore_2017' on page 2
undefined o
n input line 108.
```

```
Package natbib Warning: Citation `ewels_nf-core_2020' on page 2 undefined
on in
put line 108.


Package natbib Warning: Citation `ivie_reproducibility_2019' on page 2
undefine
d on input line 113.


Package natbib Warning: Citation `suetake_sapporo_2022' on page 2
undefined on
input line 125.

LaTeX Font Info:    Trying to load font information for T1+pcr on input
line 12
5.
(c:/TeXLive/2022/texmf-dist/tex/latex/psnfss/t1pcr.fd
File: t1pcr.fd 2001/06/04 font definitions for T1/pcr.
)
Underfull \vbox (badness 5274) has occurred while \output is active []


Package natbib Warning: Citation `danecek_twelve_2021' on page 2
undefined on i
nput line 146.


Package natbib Warning: Citation `patel_rnaseq_2022' on page 2 undefined
on inp
ut line 151.

[2]

Package natbib Warning: Citation `rehm2021ga4gh' on page 3 undefined on
input l
ine 194.


Package natbib Warning: Citation `soiland-reyes_packaging_2022' on page 3
undef
ined on input line 200.

LaTeX Font Info:    External font `lmex10' loaded for size
(Font)              <8> on input line 202.
LaTeX Font Info:    Font shape `OT1/ptm/bx/n' in size <8> not available
(Font)              Font shape `OT1/ptm/b/n' tried instead on input line
202.

Underfull \hbox (badness 10000) in paragraph at lines 202--202
[]$[]$[][]$\T1/pcr/m/n/8 https : / / raw . githubusercontent . com /
sapporo-[]
wes /
```

```
 []


Package natbib Warning: Citation `ison_edam_2013' on page 3 undefined on
input
line 209.


Package natbib Warning: Citation `danecek_variant_2011' on page 3
undefined on
input line 210.


LaTeX Warning: File `figures/example_file_entity.pdf' not found on input
line 2
44.


! Package pdftex.def Error: File `figures/example_file_entity.pdf' not
found: u
sing draft setting.

See the pdftex.def package documentation for explanation.
Type  H <return>  for immediate help.
 ...

l.244 ...xtwidth]{figures/example_file_entity.pdf}

Try typing  <return>  to proceed.
If that doesn't work, type  X <return>  to quit.


Package natbib Warning: Citation `suetake_workflow_2022' on page 3
undefined on
 input line 255.

[3]

LaTeX Warning: File `figures/tonkaz_overview.pdf' not found on input line
275.


! Package pdftex.def Error: File `figures/tonkaz_overview.pdf' not found:
using
 draft setting.

See the pdftex.def package documentation for explanation.
Type  H <return>  for immediate help.
 ...

l.275 ...8\textwidth]{figures/tonkaz_overview.pdf}

Try typing  <return>  to proceed.
If that doesn't work, type  X <return>  to quit.
```

```
LaTeX Warning: File `figures/tonkaz_repro_scale.pdf' not found on input
line 30
4.


! Package pdftex.def Error: File `figures/tonkaz_repro_scale.pdf' not
found: us
ing draft setting.

See the pdftex.def package documentation for explanation.
Type  H <return>  for immediate help.
 ...

l.304 ...extwidth]{figures/tonkaz_repro_scale.pdf}

Try typing  <return>  to proceed.
If that doesn't work, type  X <return>  to quit.


LaTeX Warning: File `figures/tonkaz_cli_example.pdf' not found on input
line 32
0.


! Package pdftex.def Error: File `figures/tonkaz_cli_example.pdf' not
found: us
ing draft setting.

See the pdftex.def package documentation for explanation.
Type  H <return>  for immediate help.
 ...

l.320 ...extwidth]{figures/tonkaz_cli_example.pdf}

Try typing  <return>  to proceed.
If that doesn't work, type  X <return>  to quit.


Package natbib Warning: Citation `depristo_framework_2011' on page 4
undefined
on input line 334.


Package natbib Warning: Citation `broad_gatk_2021' on page 4 undefined on
input
 line 334.


Package natbib Warning: Citation `ewels_nf-core_2020' on page 4 undefined
on in
put line 334.
```

```
Package natbib Warning: Citation `patel_rnaseq_2022' on page 4 undefined on inp
ut line 334.


Package natbib Warning: Citation `kodama_ddbj_2015' on page 4 undefined on inpu
t line 334.


Package natbib Warning: Citation `nbdc_whole-genome_2023' on page 4 undefined o
n input line 334.


Package natbib Warning: Citation `ddbj_jga-analysis_2023' on page 4 undefined o
n input line 334.


Package natbib Warning: Citation `suetake_tonkaz-test-methods_2022' on page 4 u
ndefined on input line 337.


Package natbib Warning: Citation `suetake_tonkaz-raw-data_2022' on page 4 undef
ined on input line 337.


Underfull \vbox (badness 10000) has occurred while \output is active []

[4]
Underfull \vbox (badness 10000) has occurred while \output is active []


Underfull \vbox (badness 10000) has occurred while \output is active []

[5]
Underfull \hbox (badness 10000) in alignment at lines 366--366
[][][][][]
 []


Package natbib Warning: Citation `mckenna_genome_2010' on page 6 undefined on i
nput line 400.


Package natbib Warning: Citation `dobin_star_2013' on page 6 undefined on input
 line 406.
```

```
Underfull \vbox (badness 10000) has occurred while \output is active []


Underfull \vbox (badness 10000) has occurred while \output is active []

[6]
Underfull \vbox (badness 10000) has occurred while \output is active []


Underfull \vbox (badness 10000) has occurred while \output is active []

[7]
Underfull \vbox (badness 10000) has occurred while \output is active []


Underfull \vbox (badness 10000) has occurred while \output is active []

[8]

Package natbib Warning: Citation `pertea_stringtie_2015' on page 9
undefined on
 input line 418.


Package natbib Warning: Citation `nf-core_test-datasets_2018' on page 9
undefin
ed on input line 426.


Package natbib Warning: Citation `suetake_small-dataset_2022' on page 9
undefin
ed on input line 426.


Underfull \hbox (badness 2376) in paragraph at lines 431--435
\T1/ptm/m/n/10 where all the out-put files were com-pared. Most of
 []


Underfull \hbox (badness 1661) in paragraph at lines 431--435
\T1/ptm/m/n/10 the files were Level 3 or Level 2; how-ever, 16 files
 []


Underfull \hbox (badness 10000) in paragraph at lines 431--435
\T1/ptm/m/n/10 ple, \T1/pcr/m/n/10 mqc_mqc_mplplot_gtnuqiebfc_1.pdf
\T1/ptm/m/n
/10 and
 []


Overfull \hbox (5.61159pt too wide) in paragraph at lines 431--435
\T1/pcr/m/n/10 execution_report_2022-09-08_06-28-19.html\T1/ptm/m/n/10 .
```

```
 []


Underfull \vbox (badness 1297) has occurred while \output is active []


Package natbib Warning: Citation `nust_codecheck_2021' on page 9
undefined on i
nput line 449.

[9]

Package natbib Warning: Citation `ewels_multiqc_2016' on page 10
undefined on i
nput line 470.


Package natbib Warning: Citation `wf-run_wf-run-crate_2023' on page 10
undefine
d on input line 472.


Underfull \vbox (badness 3068) has occurred while \output is active []


Package natbib Warning: Citation `baker_1500_2016' on page 10 undefined
on inpu
t line 476.


Underfull \hbox (badness 6063) in paragraph at lines 489--490
[]\T1/ptm/m/n/10 Project home page: $\T1/pcr/m/n/10 https : / / github .
com /
 []

[10]
Underfull \hbox (badness 6063) in paragraph at lines 504--505
[]\T1/ptm/m/n/10 Project home page: $\T1/pcr/m/n/10 https : / / github .
com /
 []


Underfull \hbox (badness 6063) in paragraph at lines 519--520
[]\T1/ptm/m/n/10 Project home page: $\T1/pcr/m/n/10 https : / / github .
com /
 []


Package natbib Warning: Citation `suetake_tonkaz-test-methods_2022' on
page 11
undefined on input line 535.
```

```
Package natbib Warning: Citation `suetake_test-workflow_2022' on page 11
undefi
ned on input line 536.


Package natbib Warning: Citation `suetake_tonkaz-raw-data_2022' on page
11 unde
fined on input line 537.

No file main.bbl.

Package natbib Warning: There were undefined citations.

- LAST -
Extra skip:0.0pt
Left:454.43332pt/0.0pt
Right:479.24968pt/4.20004pt
Split: 475.59148pt
Output:475.59148pt
Pageshrink: 8.0pt
Pagestretch: 15.0pt
@colht:691.40196pt
FLUSHEND [output]: 475.59148pt
FLUSHEND [leftcolumn badness]: 7
FUSHEND [rightcolumn badness]: 2
[11]
enddocument/afterlastpage: lastpage setting LastPage.
(./main.aux) )
(\end occurred inside a group at level 1)

### semi simple group (level 1) entered at line 15 (\begingroup)
### bottom level
Here is how much of TeX's memory you used:
 28253 strings out of 476093
 539390 string characters out of 5795584
 1881787 words of memory out of 5000000
 48174 multiletter control sequences out of 15000+600000
 622070 words of font info for 141 fonts, out of 8000000 for 9000
 1141 hyphenation exceptions out of 8191
 87i,14n,131p,1227b,3803s stack positions out of
10000i,1000n,20000p,200000b,200000s
{c:/TeXLive/2022/texmf-dist/fonts/enc/dvips/lm/lm-rm.enc}{c:/Te
XLive/2022/texmf-dist/fonts/enc/dvips/base/8r.enc}{c:/TeXLive/2022/texmf-
dist/f
onts/enc/dvips/lm/lm-mathit.enc} <c:/Users/asynch/.texlive2022/texmf-
var/fonts/
pk/ljfour/public/ifsym/ifsym10.420pk><c:/TeXLive/2022/texmf-
dist/fonts/type1/bi
tstrea/charter/bchb8a.pfb><c:/TeXLive/2022/texmf-
dist/fonts/type1/public/lm/lmm
i7.pfb><c:/TeXLive/2022/texmf-
dist/fonts/type1/public/lm/lmr6.pfb><c:/TeXLive/2
022/texmf-dist/fonts/type1/urw/courier/ucrr8a.pfb><c:/TeXLive/2022/texmf-
dist/f
```

```
onts/type1/urw/helvetic/uhvb8a.pfb><c:/TeXLive/2022/texmf-
dist/fonts/type1/urw/
helvetic/uhvbo8a.pfb><c:/TeXLive/2022/texmf-
dist/fonts/type1/urw/helvetic/uhvr8
a.pfb><c:/TeXLive/2022/texmf-
dist/fonts/type1/urw/helvetic/uhvro8a.pfb><c:/TeXL
ive/2022/texmf-
dist/fonts/type1/urw/times/utmb8a.pfb><c:/TeXLive/2022/texmf-dis
t/fonts/type1/urw/times/utmbi8a.pfb><c:/TeXLive/2022/texmf-
dist/fonts/type1/urw
/times/utmr8a.pfb><c:/TeXLive/2022/texmf-
dist/fonts/type1/urw/times/utmri8a.pfb
>
Output written on main.pdf (11 pages, 175838 bytes).
PDF statistics:
 108 PDF objects out of 1000 (max. 8388607)
 69 compressed objects within 1 object stream
 0 named destinations out of 1000 (max. 500000)
 1 words of extra memory for PDF output out of 10000 (max. 10000000)
```

```json
{
  "@context": ["https://w3id.org/ro/crate/1.1/context", { ... }],
  "@graph": [
    ...,
    {
      "@id": "outputs/star_salmon/RAP1_UNINDUCED_REP2.markdup.sorted.bam",
      "@type": ["File", "FormalParameter", "OutputFile"],
      "contentSize": 3279083,
      "dateModified": "2022-09-08T08:52:19.755363",
      "encodingFormat": "application/gzip",
      "format": {
        "@id": "http://edamontology.org/format_2572"
      },
      "gid": 1000,
      "mode": "-rw-r--r--",
      "sha512": "2d6c8436dd1da0e4e49f9bdfbf8d656d7740f7eae149bb2add417d6739c05aeb441aa84239041f9aac87688042c7b31bfca5c95d0dfaf742512f2e740a788979",
      "stats": {
        "@id": "#31d3ba80-21df-4ab7-93e7-558154d07161"
      },
      "uid": 1000,
      "url": "http://localhost:1122/runs/93f4d8bf-424d-4d5e-bc79-9482e1620be9/data/outputs/star_salmon/RAP1_UNINDUCED_REP2.markdup.sorted.bam"
    },
    {
      "@id": "#31d3ba80-21df-4ab7-93e7-558154d07161",
      "@type": ["FileStats"],
      "duplicateRate": 0.8027008887713803,
      "duplicateReads": 78936,
      "generatedBy": {
        "@id": "#samtools"
      },
      "mappedRate": 1.0,
      "mappedReads": 98338,
      "totalReads": 98338,
      "unmappedRate": 0.0,
      "unmappedReads": 0
    },
    ...
  ]
}
```

LaTeX - Figure (eps, ps, etc.)
Click here to access/download;LaTeX - Figure (eps, ps, etc.);tonkaz_cli_example.pdf

```
...

=== Level3 ⭐⭐⭐ (Same Checksum, 13/25 files)

  - star_salmon/rseqc/junction_annotation/bed/RAP1_IAA_30M_REP1.junction.Interact.bed
  - star_salmon/rseqc/junction_annotation/bed/RAP1_IAA_30M_REP1.junction.bed
  - star_salmon/rseqc/junction_annotation/bed/RAP1_UNINDUCED_REP1.junction.Interact.bed
  - star_salmon/rseqc/junction_annotation/bed/RAP1_UNINDUCED_REP1.junction.bed
  - star_salmon/rseqc/junction_annotation/bed/RAP1_UNINDUCED_REP2.junction.Interact.bed
  - star_salmon/rseqc/junction_annotation/bed/RAP1_UNINDUCED_REP2.junction.bed
  - star_salmon/rseqc/junction_annotation/bed/WT_REP2.junction.Interact.bed
  - star_salmon/rseqc/junction_annotation/bed/WT_REP2.junction.bed
  - star_salmon/stringtie/RAP1_IAA_30M_REP1.coverage.gtf
  - star_salmon/stringtie/RAP1_UNINDUCED_REP1.coverage.gtf
  - star_salmon/stringtie/RAP1_UNINDUCED_REP2.coverage.gtf
  - star_salmon/stringtie/WT_REP1.coverage.gtf
  - star_salmon/stringtie/WT_REP2.coverage.gtf

=== Level2 ⭐⭐ (Similar Features, 12/25 files)

  - star_salmon/WT_REP1.markdup.sorted.bam
    .---------------------------------------------------------------------.
    |                |         in Crate1         |         in Crate2         |
    |----------------|---------------------------|---------------------------|
    | File Size      | 6.86 MB (7196616)         | 6.86 MB (7192272)         |
    | Total Reads    | 188243                    | 188241                    |
    |   # Mapped     | 188243 (100.00%)          | 188241 (100.00%)          |
    |   # Duplicate  | 38470 (20.44%)            | 38470 (20.44%)            |
    '---------------------------------------------------------------------'

  - star_salmon/rseqc/junction_annotation/bed/WT_REP1.junction.Interact.bed
    .---------------------------------------------------------------------.
    |                |         in Crate1         |         in Crate2         |
    |----------------|---------------------------|---------------------------|
    | File Size      | 22.46 KB (22995)          | 22.32 KB (22856)          |
    | Line Count     | 162                       | 161                       |
    '---------------------------------------------------------------------'

  - star_salmon/stringtie/WT_REP2.transcripts.gtf
    .---------------------------------------------------------------------.
    |                |         in Crate1         |         in Crate2         |
    |----------------|---------------------------|---------------------------|
    | File Size      | 37.82 KB (38729)          | 37.82 KB (38729)          |
    | Line Count     | 259                       | 259                       |
    '---------------------------------------------------------------------'

...

=== Level1 ⭐ (Different Features, 0/25 files)

=== Level0 (Not Found, Crate1: 0 files, Crate2: 0 files)

Summarize compare result:

  .---------------------------------------------------------------------------.
  |       Reproducibility        |   Level    |     Definition      |   File #    |
  |------------------------------|------------|---------------------|-------------|
  | Fully Reproduced             | ⭐⭐⭐     | Same Checksum       | 13 files    |
  | Acceptable Differences       | ⭐⭐       | Similar Features    | 12 files    |
  | Unacceptable Differences     | ⭐         | Different Features  | 0 files     |
  | Not Reproduced               |            | Not Found           | 0 files     |
  '---------------------------------------------------------------------------'
```

LaTeX - Figure (eps, ps, etc.)

Click here to access/download;LaTeX - Figure (eps, ps, etc.);tonkaz_overview.pdf

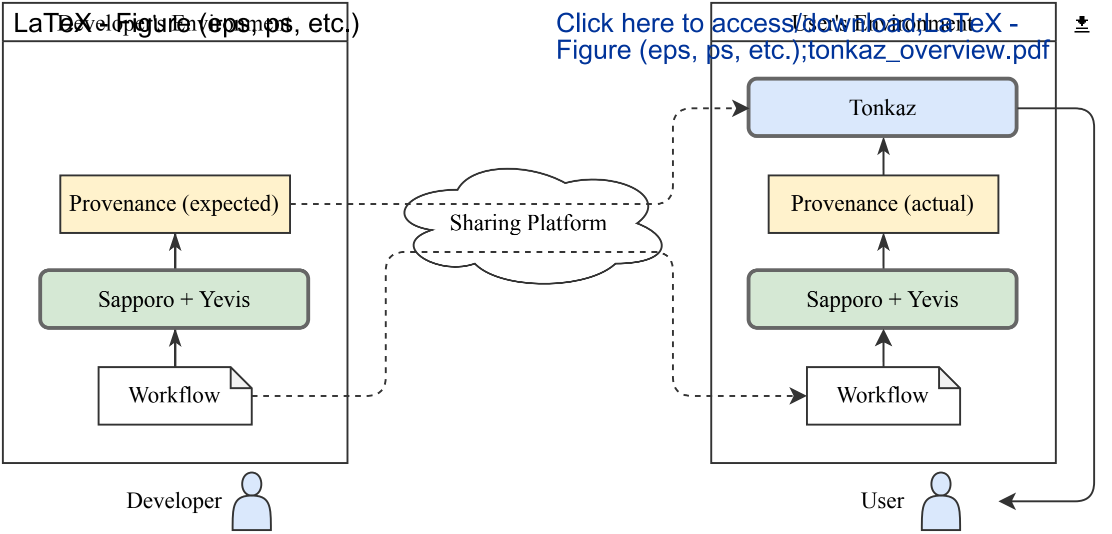

LaTeX - Figure (eps, ps, etc.)
Click here to access/download;LaTeX - Figure

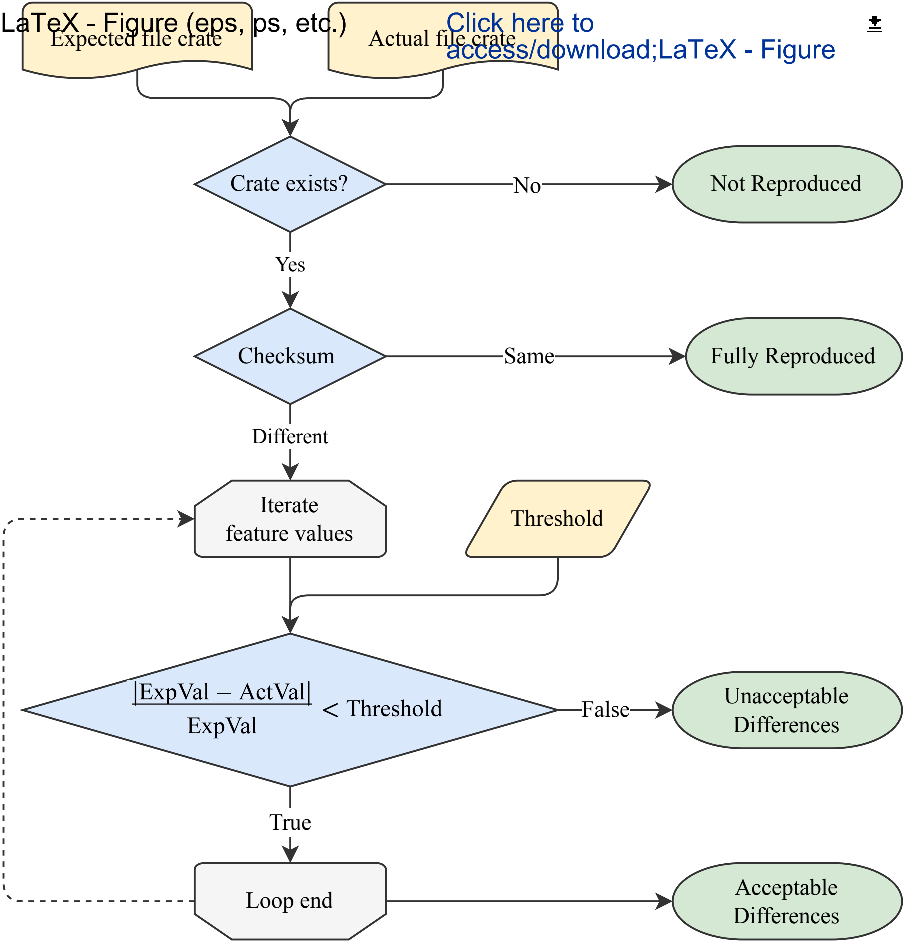

Manuscript
Click here to access/download;Manuscript;Tonkaz_Paper___bioRxiv (1).pdf

A workflow reproducibility scale for automatic validation of biological interpretation results

Hirotaka Suetake[1], Tsukasa Fukusato[2], Takeo Igarashi[3], and Tazro Ohta[4]✉

[1]Department of Creative Informatics, Graduate School of Information Science and Technology, The University of Tokyo, Tokyo, Japan. ORCID: https://orcid.org/0000-0003-2765-0049
[2]Department of Computer Science, Graduate School of Information Science and Technology, The University of Tokyo, Tokyo, Japan. ORCID: https://orcid.org/0000-0002-5090-1443
[3]Department of Creative Informatics, Graduate School of Information Science and Technology, The University of Tokyo, Tokyo, Japan. ORCID: https://orcid.org/0000-0002-5495-6441
[4]Database Center for Life Science, Joint Support-Center for Data Science Research, Research Organization of Information and Systems, Shizuoka, Japan. ORCID: https://orcid.org/0000-0003-3777-5945

Background: **Reproducibility of data analysis workflow is a key issue in the field of bioinformatics. Recent computing technologies, such as virtualization, have made it possible to reproduce workflow execution with ease. However, the reproducibility of results is not well discussed; that is, there is no standard way to verify whether the biological interpretation of reproduced results are the same. Therefore, it still remains a challenge to automatically evaluate the reproducibility of results.**

Results: **We propose a new metric, a reproducibility scale of workflow execution results, to evaluate the reproducibility of results. This metric is based on the idea of evaluating the reproducibility of results using biological feature values (e.g., number of reads, mapping rate, and variant frequency) representing their biological interpretation. We also implemented a prototype system that automatically evaluates the reproducibility of results using the proposed metric. To demonstrate our approach, we conducted an experiment using workflows used by researchers in real research projects and the use cases that are frequently encountered in the field of bioinformatics.**

Conclusions: **Our approach enables automatic evaluation of the reproducibility of results using a fine-grained scale. By introducing our approach, it is possible to evolve from a binary view of whether the results are superficially identical or not to a more graduated view. We believe that our approach will contribute to more informed discussion on reproducibility in bioinformatics.**

Workflow | Provenance | Reproducibility
Correspondence: ***t.ohta@dbcls.rois.ac.jp***

Background

Bioinformatics is big data science and is considered the most demanding domain in terms of data acquisition, storage, distribution, and analysis (1). Because the low cost and high throughput of measurement instruments have made it possible to generate large amounts of data, large-scale data analysis using a computer is required to extract valuable knowledge from the data (2, 3). For each data analysis process, such as data transformation, public database referencing and merging, and statistical processing, much open-source software is developed and released (4). Researchers typically choose appropriate software for each analysis process, build a workflow by combining the software, and execute the workflow in a computing environment (5). However, it can be challenging to ensure the reproducibility of data analysis due to a number of factors, such as a large amount of data, the diversity of data types and software, and the complexity of the computing environment (6).

Reproducibility of research is an essential issue in the scientific community (7, 8). However, Baker raised the alarm of a "reproducibility crisis" based on survey results that *"more than 70% of researchers have tried and failed to reproduce another scientist's experiments, and more than half have failed to reproduce their own experiments"* (9, p. 452). The key here is the requirement for research to be considered reproducible. Drummond argued that replicability and reproducibility are often confused, but they are different concepts and need to be clearly distinguished (10). The Association for Computing Machinery (ACM) also attempts to define the terms repeatability, reproducibility, and replicability (Table 1) (11). While these definitions are in the context of computerized analysis, it should be noted that most existing studies have focused on whether the execution can be reproduced or not and have not considered the verification of the results. That is, they only state that the resulting data are exactly the same as in the original but do not adequately discuss the verification of whether the results are reproducible or not. Therefore, the reproducibility of data analysis can be divided into two parts: the execution of the analysis and the verification of the results. We will focus our discussion on the second part, verification.

DRAFT

Many workflow systems have been developed to improve the efficiency of building and executing complex data analysis (12–14). Each system has unique characteristics, but in particular, workflow systems can have a syntax for describing the data analysis, called a workflow language. Large user communities have been formed around these workflow languages. The Common Workflow Language (CWL) (15), the Workflow Description Language (WDL) (16), Nextflow (17), and Snakemake (18) are typical examples. These systems also have execution systems that work with computational frameworks, such as job schedulers, container runtimes, and package managers. Thus, these workflow systems facilitate the execution of data analysis by different teams and in different environments through the use of virtualization technology and syntax that abstracts software and computational require-

Table 1. Repeatability, reproducibility, and replicability. According to the ACM (11), repeatability is defined as a researcher can reliably repeat her own computation. Reproducibility is defined as an independent group can obtain the same result using the author's own artifacts, and replicability is defined as an independent group can obtain the same result using artifacts which they develop completely independently.

Term	Team	Environment	Setup (code and data)	Result
Repeatability	Same	Same	Same	Same
Reproducibility	Different	Different	Same	Same
Replicability	Different	Different	Different	Same

ments (19).

The advent and widespread use of workflow systems have facilitated data analysis re-execution. However, as mentioned earlier, to ensure reproducibility, it is necessary to verify the execution results, that is, whether the same biological interpretation is obtained or not. To address this issue, frameworks, such as Research Object Crate (RO-Crate) (20) and CWLProv (21), have been proposed to generate workflow provenance, a structured archive that packages workflow-related metadata, such as workflow descriptions, execution parameters, input and output data, tests, and documentation, in a machine-readable format. This provenance information is distributed on workflow sharing platforms, such as WorkflowHub (22), Dockstore (23), and nf-core (24). When appropriate provenance is provided by the author, a researcher can use this information to verify new execution results making the process reproducible.

However, the process of comparing the provenance and execution results is often incomplete and inefficient. In automatic comparison, the checksums of the output files are used; however, they do not always match. This is because these checksums may differ depending on the software version, timestamps, heuristic algorithms, and computing environments (e.g., OS and CPU architecture, etc.) (25). However, the same biological interpretation may be obtained even when the output files do not match exactly; for example, only the timestamps in the output files may differ. Thus, a simple comparison using a checksum is incomplete in verifying results. Another method is to have humans semantically interpret the results. However, due to its inefficiency, this method is not possible when the scale of the data analysis execution is large. From the above, the verification of results using provenance remains challenging because the current procedure is limited to incomplete automatic comparison and inefficient manual checking.

Automation is essential for the verification of practical workflows that output many files; however, binary determination by checking checksums is not sufficient. Thus, it is necessary to introduce a fine-grained scale to determine the degree of reproducibility of the results. Automatic verification of results using this scale will make verification of workflow reproducibility practical. In this paper, we propose a reproducibility scale of workflow execution results based on some discussion and experiments and a validation method using this scale. We implement a workflow execution system that generates a workflow provenance that contains metadata required for verification. This implementation is an extension of Sapporo (26) `(RRID:SCR_023202)`, an existing workflow execution service (WES). Sapporo's extensibility makes it compatible with various workflow languages and execution systems. In addition, we adapt RO-Crate as the workflow provenance format. We also develop Tonkaz: a command-line tool that verifies the reproducibility of data analysis results by comparing the workflow provenances. To demonstrate the effectiveness of our approach, we apply it to workflows used by researchers in real research projects. The full reproducibility of research is still an issue that has not been fully resolved. Nevertheless, we hope our approach will contribute to solving this problem by increasing the resolution of the definition of reproducibility.

DRAFT

Methods

Reproducibility scale of workflow execution results. A workflow is a sequence of computational steps that combine analysis tools according to their inputs and outputs. The first tool takes input data and passes its output on as input for the next tool. Thus, the result of the workflow execution is the cumulative output of each tool or the last tool in the workflow. It should be noted that the output of a tool includes not only output files, but also execution logs (e.g., standard output and error) and runtime information (e.g., exit code, start time, and end time). Returning to the purpose of data analysis here, it is to obtain useful biological knowledge from the data. Therefore, it is not sufficient to consider the output files and logs as the only result of the workflow execution; the biological feature values interpreted from the output files and logs should be considered as the result of the workflow execution.

The format to represent biological features obtained from data analysis is not standardized and varies depending on the analysis tool. For example, there are summarized formats (tabular and graph) and formats that express biological features themselves, such as Sequence Alignment/Map (SAM) and the Variant Call Format (VCF). To interpret and verify the results, the individual executing the workflow visually checks the output graph or uses a tool to extract a numerical feature value from the file, for example, SAMtools (27) `(RRID:SCR_002105)` to extract mapping statistics from the SAM format. Because these processes require domain knowledge, it is ideal that the workflow itself provides a structured summary and a way to interpret it. However, this depends on the skill and effort of the individual workflow developer, and the diversity of tools and workflows makes it challenging to provide them in a standardized way.

There are several workflows that provide a way to verify reproducibility using biological feature values. For example,

the RNA-seq workflow (28) distributed by the nf-core project has a test mode to verify that the workflow is working as expected. In this mode, the workflow is executed with a small test dataset, and the biological feature values are compared with the expected values. The mapping rate, which represents the percentage of reads that are mapped to the reference genome, is used as a biological feature value. If the difference between the values is within the threshold, the workflow is considered to be working as expected. As a preliminary experiment, we compared the output files without using such biological feature values—that is, the checksum method was used to verify an exact match of a file. As a result, when we executed the above RNA-seq workflow twice in the same environment and compared the file output BAM files (the compressed binary version of the SAM files), we found that the checksum values were different and the file sizes differed by several bytes (see the Section "Results" for details). It is ideal that the output files are exactly the same, but it is difficult to achieve this goal because the output files are generated by the analysis tools, and these tools are not designed to produce the same output all the time. Therefore, we concluded that it is not sufficient to check only the exact match of output files to verify the reproducibility of workflow execution results and that a method using biological feature values and threshold should also be introduced.

Based on the above discussion and preliminary experiments, we propose a method to verify the reproducibility of workflow execution results using biological feature values and threshold. The method consists of two steps: (1) extracting biological feature values from the output files and logs and (2) comparing the extracted biological feature values with the expected values using threshold values. A detailed description of each step is provided in the Sections "Generation of workflow provenance containing biological feature values" and "Automatic verification of reproducibility." We also propose a scale to evaluate the reproducibility of workflow execution results based on the method (Table 2). This allows the reproducibility of results to be expressed at a higher resolution than a binary measure of whether the results are the same or not.

Generation of workflow provenance containing biological feature values. To verify the reproducibility of workflow execution results using biological feature values, it is necessary to package the workflow execution results as the workflow provenance in a standardized format. Because there are many workflow languages and execution engines, we first abstracted the workflow execution itself. Thus, we extended Sapporo, an existing WES implementation. Sapporo has an API scheme that satisfies the Global Alliance for Genomics and Health (GA4GH) Workflow Execution Service (WES) standard (29), enabling the workflow execution and results acquisition in a standardized manner. In addition, due to its extensibility, it can execute workflows written in various languages, such as CWL, WDL, Nextflow, and Snakemake. Therefore, by extending Sapporo, workflow execution written in various languages can be handled in the same way.

When a workflow is executed in Sapporo, the files related to the execution are stored in the file system as workflow provenance. This provenance directory contains the workflow definition files, input files, intermediate files, output files, log files, execution parameters, runtime information, etc. Thus, we converted Sapporo's provenance into RO-Crate (20), a standardized format for packaging research objects expressed in JSON-LD. Because the RO-Crate use case included the packaging of workflow execution results, it was sufficient to map Sapporo's provenance to the ontology provided by RO-Crate. However, for verification, we defined some additional terms and properties[1]. For example, we defined the property "mappedRate" to represent the mapping rate of the output file, which is a biological feature value used for verification. In addition, RO-Crate is designed to rely on the local file system for file location resolution and checksum representation. However, we prioritized the portability of being able to carry the provenance in a single file, so we put all the information necessary for verification, such as checksums, biological feature values, and contents of files of small size, in the RO-Crate file.

Because the workflow output freely produces a large number of diverse files, it is impractical to extract biological feature values for all files. Thus, we used the file extension to determine the file type and used an appropriate tool to extract the biological feature values. We used the file types defined in the EDAM ontology (30) `(RRID:SCR_006620)`, which are widely used to express biological interpretations (Table 3). For example, if the file type is SAM, we used SAMtools to extract the number of reads and the mapping rate, and if the file type is VCF, we used VCFtools (31) `(RRID:SCR_001235)` to extract the number of variants and the variant frequency. In addition, the number of lines in the file is also a biological feature value. For example, if the file type is FASTQ, four lines represent one sequence read. Figure 1 is an example of a file entity in RO-Crate, which contains the biological feature values, such as the statistics obtained from the file, file size, and the number of lines.

For provenance enrichment and sharing, we integrated Sapporo and Yevis (32) `(RRID:SRC_023204)`. Yevis is a system that builds a workflow registry and also acts as a client for Sapporo. The workflow metadata file used in Yevis contains not only the information required to execute the workflow but also the information for workflow availability and traceability in workflow sharing, such as author, open-source license, and documentation link. Thus, by executing the workflow in Sapporo via Yevis, the availability and traceability of the generated provenance are improved. In addition, because Yevis' workflow sharing feature enables the attachment of generated provenance to shared workflows, the reproducibility of shared workflows can be verified by other users.

From the above, by executing the workflow with Sapporo and Yevis (Figure 2), a workflow provenance containing feature values representing a biological interpretation is generated as RO-Crate. This method also applies to workflows written in

[1] `https://raw.githubusercontent.com/sapporo-wes/sapporo-service/main/sapporo/ro-terms.csv`

DRAFT

Table 2. Reproducibility scale of workflow execution results. For each of the output files, this is determined by comparing the expected provenance with the provenance of the actual execution. If the file of the same name in the expected provenance and the actual execution are identical, the file is considered to be fully reproduced. If the file of the same name is not identical, it is determined whether its difference is acceptable or not using the feature and threshold values. If the difference is acceptable, the file is considered to be partially reproduced. If the file exists in the expected provenance but not in the actual execution, it is considered to be not reproduced.

Reproducibility scale	Level	Description
Fully Reproduced	3	Output files are identical with the same checksum.
Acceptable Differences	2	Output files are not identical, but their biological feature values (e.g., number of reads, mapping rate, and variant frequency) are similar (within a threshold).
Unacceptable Differences	1	Output files are not identical, and their biological feature values are not similar (beyond threshold).
Not Reproduced	0	The workflow does not produce output files.

Table 3. File types and extensions defined in EDAM ontology. These file types and extensions are used to extract biological feature values from the output files.

EDAM ID	File type	Extension
format_1929	FASTA	.fa, .fasta
format_1930	FASTQ	.fq, .fastq, .fq.gz, .fastq.gz
format_1975	GFF	.gff, .gff3
format_2306	GTF	.gtf
format_2572	BAM	.bam
format_2573	SAM	.sam
format_3003	Bed	.bed
format_3004	BigBed	.bb
format_3005	Wig	.wig
format_3006	BigWig	.bw
format_3016	VCF	.vcf, .vcf.gz

```json
{
  "@context": ["https://w3id.org/ro/crate/1.1/context", { ... }],
  "@graph": [
    ...,
    {
      "@id": "outputs/star_salmon/RAP1_UNINDUCED_REP2.markdup.sorted.bam",
      "@type": ["File", "FormalParameter", "OutputFile"],
      "contentSize": 3279083,
      "dateModified": "2022-09-08T08:52:19.755363",
      "encodingFormat": "application/gzip",
      "format": {
        "@id": "http://edamontology.org/format_2572"
      },
      "gid": 1000,
      "mode": "-rw-r--r--",
      "sha512": "2d6c8436dd1da0e4e49f9bdfbf8d656d7740f7eae149bb2add417d6739c05aeb441aa84239041f9aac87688042c7b31bfca5c95d0dfaf742512f2e740a788979",
      "stats": {
        "@id": "#31d3ba80-21df-4ab7-93e7-558154d07161"
      },
      "uid": 1000,
      "url": "http://localhost:1122/runs/93f4d8bf-424d-4d5e-bc79-9482e1620be9/data/outputs/star_salmon/RAP1_UNINDUCED_REP2.markdup.sorted.bam"
    },
    {
      "@id": "#31d3ba80-21df-4ab7-93e7-558154d07161",
      "@type": ["FileStats"],
      "duplicateRate": 0.8027008887713803,
      "duplicateReads": 78936,
      "generatedBy": {
        "@id": "#samtools"
      },
      "mappedRate": 1.0,
      "mappedReads": 98338,
      "totalReads": 98338,
      "unmappedRate": 0.0,
      "unmappedReads": 0
    },
    ...
  ]
}
```

Fig. 1. An example of a file entity in RO-Crate. This is a part of the actual workflow execution results and uses the RNA-seq workflow distributed by the nf-core project. The output file in this example is a BAM file, and its biological feature values are the file size, number of mapped reads, and mapping rate. Thus, the file entity contains properties such as “contentSize,” “stats:mappedReads,” and “stats:mappedRate”. These are defined as additional terms in Sapporo, and the values are extracted from the file using SAMtools.

various languages and can address a wide range of use cases, such as workflow sharing. Therefore, by generating and sharing a provenance containing biological feature values, it is possible to verify the reproducibility of the workflow execution results in other users' environments.

Automatic verification of reproducibility. We developed Tonkaz to automatically verify the reproducibility of workflow execution results by comparing the biological feature values contained in the workflow provenance. One use case of Tonkaz is to compare the expected result, which is provided by a workflow developer, and the actual result, which is generated in the user's environment (Figure 2). That is, Tonkaz verifies that the results are the same according to the ACM's definition of reproducibility (Table 1). Another use case is ACM's definition of repeatability, which is to verify that the results are the same even if a workflow is executed multiple times in the same environment, and it will not be broken by updates to dependencies. Thus, these use cases indicate that we must verify the reproducibility of the results, regardless of the differences in execution methods and environments.

We designed Tonkaz to accept as arguments two RO-Crates, one containing the expected provenance and the other containing the actual provenance. Tonkaz compares the biological feature values of the output files in the two RO-Crates and calculates the reproducibility scale for each file. Among the various types of output files, including analysis results, summary reports, or execution logs, the system needs to select the files to compare. We aimed to compare the analysis results that led to a biological interpretation and to avoid the comparison of the output files that are not in a standard format. Therefore, we selected the file types to be compared as an initial set and selected the corresponding EDAM ontology terms listed in Table 3. With this selection, for example, the nf-core RNA-seq workflow produces 872 files, but only 25 files are assigned to the EDAM ontology and compared. In the process of comparing files and calculating the reproducibility scale, Tonkaz first checks whether the files are identical using a checksum (Figure 3). If the files are identical, the reproducibility scale is "Fully Reproduced." If the files are not identical, Tonkaz compares the biological feature values of the files using a threshold value to determine whether the differences are acceptable or not. The default threshold value used is 0.05, but this value can be changed according to the use case. This is because some workflows for medical applications or quality control of biological materials require a lower threshold value. The comparison result is finally summarized in a table, and the reproducibility scale of each file is also displayed in a table (Figure 4). However, Tonkaz does not score the reproducibility of the entire workflow. This is because, again, the purpose of comparison may differ depending on the use case, and it is not practical to automate the final decision. Thus, we implemented an option to generate structured data in Tonkaz. In addition, we believe that workflow developers should use this option and write conditions or scripts to determine the reproducibility for each use case.

Results

To demonstrate the effectiveness of our approach, we verified the reproducibility of workflow execution results by comparing the results of public workflows used by researchers in real research projects, not simple ones for testing. We selected these workflows as they are fairly representative and mature current best-practice for sequencing pipelines, implemented in different but typical workflow systems, and have similar set of genomics features that we can assess for provenance comparison. This verification was based on the following five practical use cases: (1) execution in the same environment, (2) execution in a different environment, (3) execution of different versions of the workflow, (4) execution with missing datasets, and (5) comparison using all output files. We used the following three workflows: (1) the mitochondrial short variant discovery workflow distributed a GATK best practice workflow (hereafter referred to as GATK workflow, language: WDL) (33, 34), (2) RNA-Seq workflow distributed by nf-core (hereafter referred to as RNA-seq workflow, language: Nextflow) (24, 28), and (3) GATK best practice-compatible germline short variant discovery workflow, which is used to process whole-genome sequencing data of the Japanese Genotype-phenotype Archive (hereafter referred to as JGA workflow, language: CWL) (35–37). We used the following two execution environments: (1) Ubuntu 20.04 LTS (CPU: Intel Xeon E5-2640 @ 2.50GHz, RAM: 24GB, Docker: 20.10.8) and (2) macOS 12.5.1 (CPU: Apple M1 Max, RAM: 64GB, Docker: 20.10.16). Table 4 shows the setting for each execution as a combination, and Table 5 summarizes the verification results based on the use cases. The methods and results of the workflow execution and verification are described in the online documentation "sapporo-wes/tonkaz - tests/README.md" (38) and are published on Zenodo (39).

The Comparisons C1, C3, and C5 present execution results in the same environment. Comparison C1 was performed using the GATK workflow, and the output file types were BAM and VCF. The reproducibility scale value was Level 2 (Acceptable Difference) for all files, with no differences in biological feature values expressing biological interpretation (e.g., mapping rate and variant frequency). The difference between these files was due to the fact that both the BAM and VCF files included the file paths of the original input file and timestamps in the header lines. Thus, when using the analysis tool GATK (40), it is challenging to fully reproduce the output files because of the behavior that the output files contain the file paths and timestamps. Comparison C3 was performed using the JGA workflow, and the output file types were VCF. This result also showed no differences in biological feature values, and the differences in file contents were due to the behavior of GATK. Comparison C5 was performed using the RNA-seq workflow, and the output file types were BAM, GTF, and BED. All GTF and BED files were Level 3 (Fully Reproduced), and all BAM files were Level 2 (Acceptable Difference). The difference between BAM files was due to the different order of the mapped reads in the BAM file. These BAM files were mapped by

DRAFT

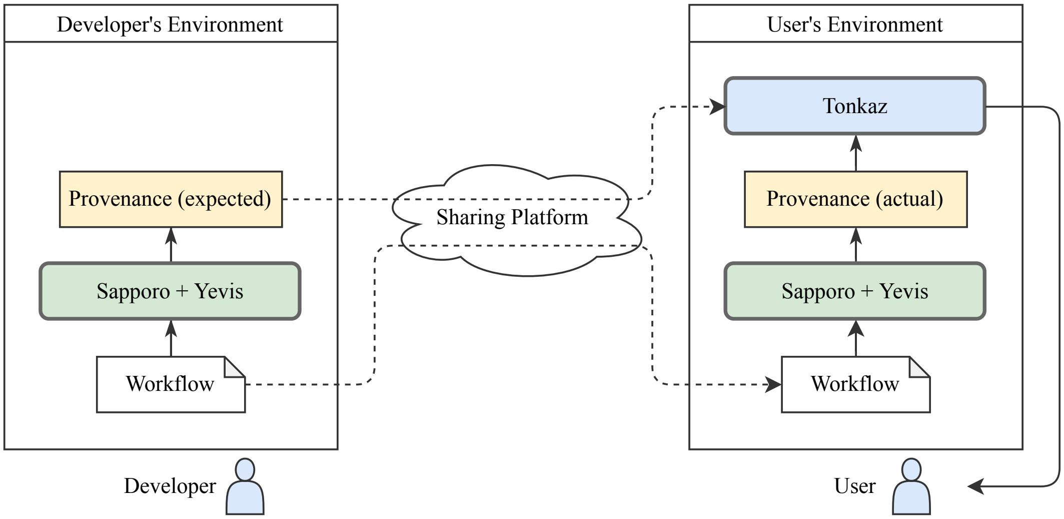

Fig. 2. The flowchart representing the Tonkaz use case. The workflow built by the workflow developer is executed by WES, which is a combination of Sapporo and Yevis, and the workflow provenance, including feature values of the output files, is generated in RO-Crate format. This provenance is used as the expected value for the verification of reproducibility. Using the shared workflow, the user executes the shared workflow in his/her own environment using WES. Using Tonkaz, the user then compares the shared provenance with the provenance generated by the user's workflow execution and verifies the reproducibility of the workflow execution results.

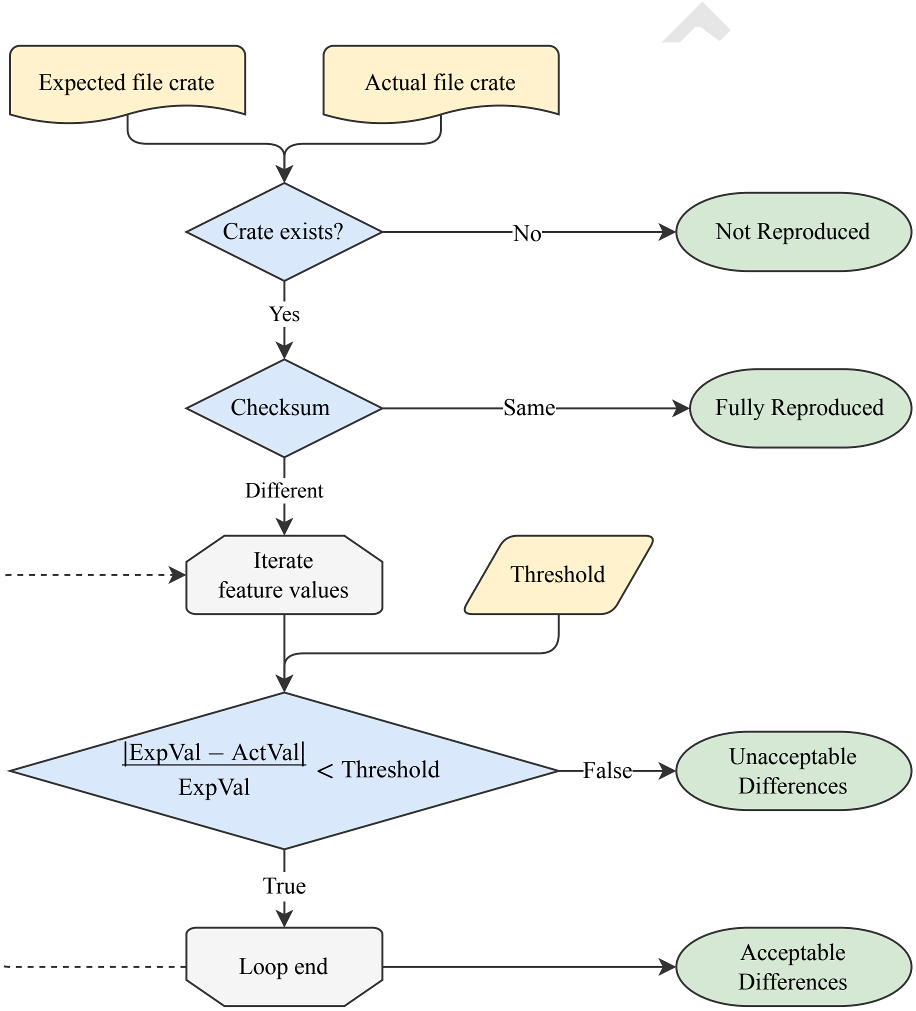

Fig. 3. The process for calculating the reproducibility scale of a file. Tonkaz first checks whether the files are identical using a checksum. If the files are identical, the reproducibility scale value is "Fully Reproduced." If the files are not identical, Tonkaz compares the biological feature values of the files using a threshold value to determine whether the differences are acceptable or not. If the differences are acceptable, the reproducibility scale value is "Acceptable Difference." If the differences are unacceptable, the reproducibility scale value is "Unacceptable Difference." The default threshold value used is 0.05, but this value can be changed according to the use case. If the file entity exists only in one of the two RO-Crates, the reproducibility scale value is "Not Reproduced."

```
...

=== Level3 ⭐⭐⭐ (Same Checksum, 13/25 files)

  - star_salmon/rseqc/junction_annotation/bed/RAP1_IAA_30M_REP1.junction.Interact.bed
  - star_salmon/rseqc/junction_annotation/bed/RAP1_IAA_30M_REP1.junction.bed
  - star_salmon/rseqc/junction_annotation/bed/RAP1_UNINDUCED_REP1.junction.Interact.bed
  - star_salmon/rseqc/junction_annotation/bed/RAP1_UNINDUCED_REP1.junction.bed
  - star_salmon/rseqc/junction_annotation/bed/RAP1_UNINDUCED_REP2.junction.Interact.bed
  - star_salmon/rseqc/junction_annotation/bed/RAP1_UNINDUCED_REP2.junction.bed
  - star_salmon/rseqc/junction_annotation/bed/WT_REP2.junction.Interact.bed
  - star_salmon/rseqc/junction_annotation/bed/WT_REP2.junction.bed
  - star_salmon/stringtie/RAP1_IAA_30M_REP1.coverage.gtf
  - star_salmon/stringtie/RAP1_UNINDUCED_REP1.coverage.gtf
  - star_salmon/stringtie/RAP1_UNINDUCED_REP2.coverage.gtf
  - star_salmon/stringtie/WT_REP1.coverage.gtf
  - star_salmon/stringtie/WT_REP2.coverage.gtf

=== Level2 ⭐⭐ (Similar Features, 12/25 files)

  - star_salmon/WT_REP1.markdup.sorted.bam
    .---------------------------------------------------------------------.
    |                 |        in Crate1          |        in Crate2          |
    |-----------------|---------------------------|---------------------------|
    | File Size       | 6.86 MB (7196616)         | 6.86 MB (7192272)         |
    | Total Reads     | 188243                    | 188241                    |
    |   # Mapped      | 188243 (100.00%)          | 188241 (100.00%)          |
    |   # Duplicate   | 38470 (20.44%)            | 38470 (20.44%)            |
    '---------------------------------------------------------------------'

  - star_salmon/rseqc/junction_annotation/bed/WT_REP1.junction.Interact.bed
    .---------------------------------------------------------------------.
    |                 |        in Crate1          |        in Crate2          |
    |-----------------|---------------------------|---------------------------|
    | File Size       | 22.46 KB (22995)          | 22.32 KB (22856)          |
    | Line Count      | 162                       | 161                       |
    '---------------------------------------------------------------------'

  - star_salmon/stringtie/WT_REP2.transcripts.gtf
    .---------------------------------------------------------------------.
    |                 |        in Crate1          |        in Crate2          |
    |-----------------|---------------------------|---------------------------|
    | File Size       | 37.82 KB (38729)          | 37.82 KB (38729)          |
    | Line Count      | 259                       | 259                       |
    '---------------------------------------------------------------------'

...

=== Level1 ⭐ (Different Features, 0/25 files)

=== Level0 (Not Found, Crate1: 0 files, Crate2: 0 files)

Summarize compare result:

  .----------------------------------------------------------------------------.
  |      Reproducibility        |   Level   |     Definition       |   File #   |
  |-----------------------------|-----------|----------------------|------------|
  | Fully Reproduced            | ⭐⭐⭐    | Same Checksum        | 13 files   |
  | Acceptable Differences      | ⭐⭐      | Similar Features     | 12 files   |
  | Unacceptable Differences    | ⭐        | Different Features   | 0 files    |
  | Not Reproduced              |           | Not Found            | 0 files    |
  '----------------------------------------------------------------------------'
```

Fig. 4. Example of the Tonkaz output. Tonkaz displays a table for each file and a final summary table. The user checks those summary tables to determine the reproducibility of the entire workflow and the differences between the expected and actual files (e.g., by using the diff command).

Table 4. Combination table of workflow execution and execution settings. The first column is the definition of the execution name. In the second column and below are the workflow execution settings. The blank cells in the third and fourth columns indicate that there are no differences in the execution settings.

Execution name	Workflow	Version	Dataset	Environment
GATK_1st				Linux
GATK_2nd	GATK			Linux
GATK_mac				Mac
JGA_1st				Linux
JGA_2nd	JGA			Linux
JGA_mac				Mac
RNA-seq_1st		v3.7	Standard	Linux
RNA-seq_2nd		v3.7	Standard	Linux
RNA-seq_mac	RNA-seq	v3.7	Standard	Mac
RNA-seq_v3.6		v3.6	Standard	Linux
RNA-seq_small		v3.7	Small	Linux

Table 5. Comparisons of execution and verification results. The definition of each execution is defined in Table 4. Five use cases are assigned according to the combination of executions. In the fifth column and below are the number of files for each reproducibility scale defined in Table 2: Level 3 is "Fully Reproduced," Level 2 is "Acceptable Difference," Level 1 is "Unacceptable Difference," and Level 0 is "Not Reproduced."

ID	Source execution	Target execution	Use case	Level 3	Level 2	Level 1	Level 0
C1	GATK_1st	GATK_2nd	Same environment	0	5	0	0
C2	GATK_1st	GATK_mac	Different environment	0	0	0	5
C3	JGA_1st	JGA_2nd	Same environment	0	4	0	0
C4	JGA_1st	JGA_mac	Differentf environment	0	0	0	4
C5	RNA-seq_1st	RNA-seq_2nd	Same environment	20	5	0	0
C6	RNA-seq_1st	RNA-seq_mac	Different environment	0	0	0	25
C7	RNA-seq_1st	RNA-seq_v3.6	Different version	13	12	0	0
C8	RNA-seq_1st	RNA-seq_small	Missing dataset	8	5	7	5
C9	RNA-seq_1st	RNA-seq_2nd	All output files	557	306	1	8

DRAFT

STAR (41) (`RRID:SCR_004463`), and sorted by SAMtools; however, differences occurred. These results show cases in which the output files were not identical, although the biological feature values were equal, due to the behavior of the analysis tool.

The Comparisons C2, C4, and C6 present the execution results in different environments. All of these comparison results were Level 0 (Not Reproduced) because all execution in the Mac environment either failed or never finished. All workflows used in this experiment were Docker containerized and were designed to be very reproducible in the execution context; however, runtime errors occurred due to the Arm processor architecture of the Mac environment. Thus, even a very well-considered workflow may not be reproducible in a different environment. In such cases, it is essential to increase the debuggability of the cause of the irreproducibility of the execution results. Therefore, the importance of this debuggability indicates that it is helpful to include information about the execution environment in the workflow provenance; our approach and RO-Crate address them.

Comparison C7 presents the execution results in different versions. Workflow developers often check for workflow breakage when updating versions of analysis tools included in the workflow. In the RNA-seq workflow used in this comparison, the dependent analysis tools STAR, SAMtools, and StringTie (42) (`RRID:SCR_016323`) were updated with the workflow update from v3.6 to v3.7. As a result of the comparison (C7), the number of files with Level 2 increased compared to C5, a comparison involving the same version. The file types that became Level 2 were GTF, BED, and BAM; the GTF and BED files were newly changed from Level 3 to Level 2 when compared to C5. The differences between the GTF files were due to differences in the FPKM field values and the version of StringTie included in the header line. The BED files had a different number of lines, and the BAM files had a different number of mapped reads; however, those differences were within the threshold value. This result indicates that verification using biological feature values and threshold is effective because apparent differences occur in output due to version updates and other reasons, and it is necessary to determine whether these differences are acceptable or not.

Comparison C8 presents the execution results in a case where the input dataset was partially missing. The dataset used in RNA-seq_1st contains six sequence read files (FASTQ) (43), while the dataset used in RNA-seq_small contained four sequence read files (44). As a result of the comparison, the output files related to the sample with half the number of reads were Level 1 (Unacceptable Difference), while the sample with zero reads was Level 0. In this case, setting the threshold used for verification to, for example, 0.5 instead of 0.05 (default value) will verify that the workflow is functioning

as expected. That is, this suggests that the threshold value and final decision may vary depending on the objectives of developers and users.

Comparison C9 presents the execution results in a case where all the output files were compared. Most of the files were Level 3 or Level 2; however, 16 files were not reproduced (Level 0). These level 0 files had random names or timestamps in the file names, for example, `mqc_mqc_mplplot_gtnuqiebfc_1.pdf` and `execution_report_2022-09-08_06-28-19.html`. Therefore, it is not appropriate to use all files to verify the reproducibility of execution results; it is essential to focus on characteristic files, such as BAM and GTF files.

For the five practical use cases, we found that our approach was well suited to verify the reproducibility of the workflow execution results. In all use cases, existing methods that use checksums to verify exact file matches can produce false positives; this means that the workflow is considered not reproduced, even though it is working as expected. Therefore, it is important to introduce a reproducibility scale and verify the workflow execution results' reproducibility at higher resolutions.

Discussion

Despite its complexity, data analysis in bioinformatics is considered reproducible and is being shared. In particular, the workflows shared by nf-core and GATK best practices are well maintained and include test datasets, documentation, and open-source licenses. Ideally, all shared workflows would be like these; however, in reality, this is challenging because of the amount of work and domain knowledge required. Thus, we aim to facilitate workflow sharing by providing a workflow provenance model and a workflow provenance verification method. However, our approach is not applicable in domains where it is difficult to verify the results and inferences using a computer. In such cases, it is first necessary to discuss an ontology or structured format for representing the research.

A related project, CODECHECK (45), aims to provide the verification of the reproducibility of data analysis by a third party in scientific publishing. CODECHECK proposes a procedure similar to a peer review system, in which the workflow associated with research articles is verified at the time of publication by a reviewer called a CODECHECKER. However, this project focuses on increasing the availability of the workflow, and does not verify the execution results. As such, it is unlikely to address the case of our concern that the execution results are not exactly the same, but the conclusions of the study remain the same. Our proposed metrics, a reproducibility scale of workflow execution results, would be useful in such workflow reproducibility validation in publishing as well.

In a scientific context, automated verification is a crucial process that should be performed for various reasons. Workflow developers can use it to easily add or update code and improve development efficiency. Administrators of workflow registries can use it to perform quality control, such as checking for broken links between the analysis tools and data used internally. Users of the workflow can also use it to validate the behavior of the workflow as an acceptance test in their own environment, thereby improving the reliability of their research projects. Tonkaz aims to support these validation efforts in different use cases and promote open science.

When generating workflow provenance using a format, such as RO-Crate, it is important to consider licensing issues. The provenance includes not only the execution results, but also the executed workflow, input datasets, and software used internally. These files and software may have different licenses, and combining them under a single license can cause relicensing problems. In RO-Crate, a license can be specified for each entity; however, this approach is not currently possible as Sapporo automatically generates provenance from run requests and execution results without the original license information. This limitation can be overcome if data and software are consistently able to present their licenses, but this would require a generic method to get the license information of files retrieved from the internet.

Software begins to degrade from the moment it is developed, and it is not easy to maintain the same quality over time. Cases in which an error, including a stack trace, is thrown are quite fortunate; in many cases, the software cannot be executed in the first place, the process does not finish, or the output is inaccurate without throwing an error. Dealing with such cases and improving debuggability is accomplished by packaging the expected behavior of the software at the time it is developed. In our approach, we were able to attach information, such as OS, CPU architecture, and dependent software versions, to the expected workflow provenance due to RO-Crate's extensibility. However, when analysis tools are used internally, as in a workflow, the behavior of the analysis tools tends to be a black box. Therefore, if an option to display the reproducibility of the execution for each analysis tool is provided, it will be possible to identify the cause of the irreproducibility of the workflow execution results.

In the Result section, we showed the cases where the differences are found in the outputs but the biological interpretation will be identical. However, there are cases where users find differences that affect the interpretation even when comparing the same workflow definitions. For example, the output results may change when the workflow has a tool that dynamically uses external databases, which may be regularly updated over time. Another case when the impact on the results can be observed is a comparison of runs of the workflow which does not explicitly specify the software version nor properly packaged.

As the system allows users to change the reporting threshold in comparison to the outputs, users need to be aware of the acceptable differences in the outputs of the given workflow. Although the threshold needs to be low for workflows used in applications that require severe quality control, such as medical data analysis, users can set it higher for workflows that can generate different outputs per run. For example, workflows using external databases, or used for environmental monitoring purposes, may have outputs that vary per

DRAFT

run. The system alerts when a change was found, however, as the interpretation depends on the cases, users need to understand the reason from the workflow description.

Though Tonkaz aims to improve the reproducibility of data analysis, the system itself also has a challenge in the reproducibility of its function. The system uses the file extension to check the file type, then specifies an external tool to extract the biological features from the file to compare the workflow outputs. However, the extracted features may change by the updates in the external tools, which results in the inconsistency of the results of comparison by Tonkaz. Another issue we see in the reproducibility of the comparison is the system's dependency on Sapporo, our WES implementation. Ideally, the results, analysis summaries, logs, etc. generated by analysis tools should be in a standardized format so any system can generate comparable statistics. The bioinformatics community needs to have a consensus for such outputs of data analysis. As a related project, MultiQC attempts to summarize the results of multiple analysis tools (46) `(RRID:SCR_014982)` . The Tonkaz system may improve its future consistency by integrating with a community effort like MultiQC, which can share the effort to extract the information from the analysis tools.

We used RO-Crate to express the provenance of our study and added additional terms and properties to the "@context" declaration for verification purposes. These terms are currently located on our own GitHub repository, but we are discussing with the RO-Crate community moving them to a more authoritative location, such as `https://github.com/ResearchObject/ro-terms`. In future work, we are also considering using the Workflow Run RO-Crate profile (47), which is currently under development to capture the provenance of executing a computational workflow, instead of our custom terms.

The proposed method is currently dependent on our software implementation; however, it can be generalized by the following three steps: A) Extract the statistics of biological features from the output, B) Represent the statistics in a standardized format, C) Compare the statistics, and report in the reproducibility scale. Although we implemented A and B in Sapporo, it is ideal to let workflow execution systems have those two steps rather than a WES implementation. Once steps A and B became common, step C can be implemented in many kinds of data analysis platforms, while Tonkaz only provides a CLI interface. However, the bioinformatics community needs to have a consensus on the standardized scale for reproducibility.

In the article regarding a "reproducibility crisis," Baker quoted a Johns Hopkins microbiologist as stating, *"The next step may be identifying what is the problem and to get a consensus."* (9, p. 452). Subsequently, the proliferation of virtualization technology and workflow systems has lowered the bar for re-executing data analysis that an individual or others have previously built. Despite this, workflow developers are always anxious about whether their workflows are broken. In response to this anxiety, we realized that the cause is our binary view of whether the workflow could be reproduced or not. To remove this anxiety, we proposed a new approach to verify the reproducibility of workflows by providing a range of reproducibility of execution results. With the development of sharing platforms, workflow sharing has become more active. Therefore, we hope that by verifying reproducibility and sharing the results, more workflows will be reused with confidence, which, in turn, will lead to increased scientific progress.

Availability of source code and requirements

- Project name: Tonkaz
- Project home page: `https://github.com/sapporo-wes/tonkaz`
- DOI: 10.5281/zenodo.7559433
- biotoolsID: tonkaz
- RRID: SCR_023206
- Operating system(s): Platform independent
- Programming language: TypeScript
- Other requirements: Deno
- License: Apache License, Version 2.0

- Project name: Sapporo-service
- Project home page: `https://github.com/sapporo-wes/sapporo-service`
- DOI: 10.5281/zenodo.7559425
- biotoolsID: sapporo-service
- RRID: SCR_023202
- Operating system(s): Platform independent
- Programming language: Python
- Other requirements: Docker recommended
- License: Apache License, Version 2.0

- Project name: Yevis-cli
- Project home page: `https://github.com/sapporo-wes/yevis-cli`
- DOI: 10.5281/zenodo.7546102
- biotoolsID: yevis-cli
- RRID: SCR_023204
- Operating system(s): Platform independent
- Programming language: Rust
- Other requirements: Docker recommended
- License: Apache License, Version 2.0

DRAFT

Data availability

The workflow, result, and documentation related to the experiment described in the Section "Results" are available on GitHub and Zenodo as follows:

- Execution method and result (38)
- Workflow definitions (48)
- Raw data of workflow execution results (39)

Declarations

List of abbreviations. ACM: Association for Computing Machinery; CPU: Central Processing Unit; CWL: Common Workflow Language; GA4GH: Global Alliance for Genomics and Health; ID: Identifier; OS: Operating System; RO-Crate: Research Object Crate; SAM: Sequence Alignment/Map; VCF: Variant Call Format; WDL: Workflow Description Language; WES: Workflow Execution Service;

Ethical Approval. Not applicable for this study.

Consent for publication. Not applicable for this study.

Competing Interests. The authors declare that they have no competing interests.

Funding. This study was supported by JSPS KAKENHI (Grant Number 20J22439, assigned to H.S.), the CREST program of the Japan Science and Technology Agency (Grant Number JPMJCR17A1, assigned to T.I.), and the Life Science Database Integration Project, NBDC of Japan Science and Technology Agency.

Author's Contributions. H.S. and T.O. conceived and developed the methodology and software and conducted the investigation. H.S., T.F., and T.O. wrote the manuscript. T.F., T.O., and T.I. supervised the project. All authors read and approved the final version of the manuscript.

Acknowledgements

We acknowledge and thank the following scientific communities and their collaborative events where several of the authors engaged in irreplaceable discussions and development throughout the project: the Pitagora Meetup, Workflow Meetup Japan, NBDC/DBCLS BioHackathon Series, and RO-Crate community. We also acknowledge Prof. Masahiro Kasahara for his valuable comments on the project.

References

1. Zachary D. Stephens, Skylar Y. Lee, Faraz Faghri, Roy H. Campbell, Chengxiang Zhai, Miles J. Efron, et al. Big data: Astronomical or genomical? *PLOS Biology*, 13(7):e1002195, 2015. doi: 10.1371/journal.pbio.1002195.
2. Lincoln D. Stein. The case for cloud computing in genome informatics. *Genome Biology*, 11(5):207, 2010. doi: 10.1186/gb-2010-11-5-207.
3. Sara Goodwin, John D. McPherson, and Richard W. McCombie. Coming of age: Ten years of next-generation sequencing technologies. *Nature Reviews Genetics*, 17(6):333–351, 2016. doi: 10.1038/nrg.2016.49.
4. Pjotr Prins, Joep de Ligt, Artem Tarasov, Ritsert C. Jansen, Edwin Cuppen, and Philip E. Bourne. Toward effective software solutions for big biology. *Nature Biotechnology*, 33(7): 686–687, 2015. doi: 10.1038/nbt.3240.
5. Jeffrey M. Perkel. Workflow systems turn raw data into scientific knowledge. *Nature*, 573 (7772):149–151, 2019. doi: 10.1038/d41586-019-02619-z.
6. Anna Bánáti, Péter Kacsuk, and Miklós Kozlovszky. Evaluating the reproducibility cost of the scientific workflows. In *2016 IEEE 11th International Symposium on Applied Computational Intelligence and Informatics (SACI)*, pages 187–190, 2016-05. doi: 10.1109/saci.2016.7507367.
7. Software with impact. *Nature Methods*, 11(3):211–211, 2014. doi: 10.1038/nmeth.2880.
8. Rebooting review. *Nature Biotechnology*, 33(4):319–319, 2015. doi: 10.1038/nbt.3202.
9. Monya Baker. 1,500 scientists lift the lid on reproducibility. *Nature*, 533(7604):452–454, 2016. doi: 10.1038/533452a.
10. Chris Drummond. Replicability is not reproducibility: Nor is it good science. In *Proceedings of the Evaluation Methods for Machine Learning Workshop at the 26th ICML*, Volume 1, 2009.
11. Association for Computing Machinery. Artifact review and badging version 1.1, 2020. `https://www.acm.org/publications/policies/artifact-review-and-badging-current`.
12. Felipe da Veiga Leprevost, Valmir C. Barbosa, Eduardo L. Francisco, Yasset Perez-Riverol, and Paulo C. Carvalho. On best practices in the development of bioinformatics software. *Frontiers in Genetics*, 5, 2014. doi: 10.3389/fgene.2014.00199.
13. Laura Wratten, Andreas Wilm, and Jonathan Göke. Reproducible, scalable, and shareable analysis pipelines with bioinformatics workflow managers. *Nature Methods*, 18(10):1161–1168, 2021. doi: 10.1038/s41592-021-01254-9.
14. Peter Amstutz, Maxim Mikheev, Michael R. Crusoe, Nebojša Tijanić, and Samuel Lampa. Existing workflow systems, 2021. `https://s.apache.org/existing-workflow-systems`.
15. Michael R. Crusoe, Sanne Abeln, Alexandru Iosup, Peter Amstutz, John Chilton, Nebojša Tijanić, et al. Methods included: Standardizing computational reuse and portability with the common workflow language. 65(6):54–63, 2022. doi: 10.1145/3486897.
16. Kate Voss, Jeff Gentry, and Geraldine Van Der Auwera. Full-stack genomics pipelining with GATK4 + WDL + Cromwell. *F1000Research*, 2017. doi: 10.7490/f1000research.1114631.1.
17. Paolo Di Tommaso, Maria Chatzou, Evan W. Floden, Pablo Prieto Barja, Emilio Palumbo, and Cedric Notredame. Nextflow enables reproducible computational workflows. *Nature Biotechnology*, 35(4):316–319, 2017. doi: 10.1038/nbt.3820.
18. Johannes Köster and Sven Rahmann. Snakemake—a scalable bioinformatics workflow engine. *Bioinformatics*, 28(19):2520–2522, 2012. doi: 10.1093/bioinformatics/bts480.
19. Felipe da Veiga Leprevost, Björn A. Grüning, Saulo Alves Aflitos, Hannes L. Röst, Julian Uszkoreit, Harald Barsnes, et al. BioContainers: An open-source and community-driven framework for software standardization. *Bioinformatics*, 33(16):2580–2582, 2017. doi: 10.1093/bioinformatics/btx192.
20. Stian Soiland-Reyes, Peter Sefton, Mercè Crosas, Leyla Jael Castro, Frederik Coppens, José M. Fernández, et al. Packaging research artefacts with RO-Crate. *Data Science*, 5(2): 97–138, 2022. doi: 10.3233/ds-210053.
21. Farah Zaib Khan, Stian Soiland-Reyes, Richard O. Sinnott, Andrew Lonie, Carole Goble, and Michael R. Crusoe. Sharing interoperable workflow provenance: A review of best practices and their practical application in CWLProv. *GigaScience*, 8(11):giz095, 2019. doi: 10.1093/gigascience/giz095.
22. Carole Goble, Stian Soiland-Reyes, Finn Bacall, Stuart Owen, Alan Williams, Ignacio Eguinoa, et al. Implementing FAIR digital objects in the EOSC-Life workflow collaboratory. *Zenodo*, 2021. doi: 10.5281/zenodo.4605654.
23. Brian D. O'Connor, Denis Yuen, Vincent Chung, Andrew G. Duncan, Xiang Kun Liu, Janice Patricia, et al. The Dockstore: Enabling modular, community-focused sharing of Docker-based genomics tools and workflows. *F1000Research*, 6:52, 2017. doi: 10.12688/f1000research.10137.1.
24. Philip A. Ewels, Alexander Peltzer, Sven Fillinger, Harshil Patel, Johannes Alneberg, Andreas Wilm, et al. The nf-core framework for community-curated bioinformatics pipelines. *Nature Biotechnology*, 38(3):276–278, 2020. doi: 10.1038/s41587-020-0439-x.
25. Peter Ivie and Douglas Thain. Reproducibility in scientific computing. 51(3):1–36, 2019. doi: 10.1145/3186266.
26. Hirotaka Suetake, Tomoya Tanjo, Manabu Ishii, Bruno P. Kinoshita, Takeshi Fujino, Tsuyoshi Hachiya, et al. Sapporo: A workflow execution service that encourages the reuse of workflows in various languages in bioinformatics. *F1000Research*, 11:889, 2022. doi: 10.12688/f1000research.122924.1.
27. Petr Danecek, James K. Bonfield, Jennifer Liddle, John Marshall, Valeriu Ohan, Martin O. Pollard, et al. Twelve years of SAMtools and BCFtools. *GigaScience*, 10(2):giab008, 2021. doi: 10.1093/gigascience/giab008.
28. Harshil Patel, Phil Ewels, Alexander Peltzer, Rickard Hammarén, Olga Botvinnik, Gregor Sturm, et al. nf-core/rnaseq: nf-core/rnaseq v3.7, 2022. doi: 10.5281/zenodo.6513815.
29. Heidi L. Rehm, Angela J.H. Page, Lindsay Smith, Jeremy B. Adams, Gil Alterovitz, Lawrence J. Babb, et al. GA4GH: International policies and standards for data sharing across genomic research and healthcare. *Cell Genomics*, 1(2):100029, 2021. doi: 10.1016/j.xgen.2021.100029.
30. Jon Ison, Matúš Kalas, Inge Jonassen, Dan Bolser, Mahmut Uludag, Hamish McWilliam, et al. EDAM: An ontology of bioinformatics operations, types of data and identifiers, topics and formats. *Bioinformatics*, 29(10):1325–1332, 2013. doi: 10.1093/bioinformatics/btt113.
31. Petr Danecek, Adam Auton, Goncolo Abecasis, Cornelis A. Albers, Eric Banks, Mark A. DePristo, et al. The variant call format and VCFtools. *Bioinformatics*, 27(15):2156–2158, 2011. doi: 10.1093/bioinformatics/btr330.
32. Hirotaka Suetake, Tsukasa Fukusato, Takeo Igarashi, and Tazro Ohta. Workflow sharing with automated metadata validation and test execution to improve the reusability of published workflows. *GigaScience*, 2023. doi: 10.1093/gigascience/giad006.
33. Mark A. DePristo, Eric Banks, Ryan Poplin, Kiran V. Garimella, Jared R. Maguire, Christopher Hartl, et al. A framework for variation discovery and genotyping using next-generation DNA sequencing data. 43(5):491–498, 2011. doi: 10.1038/ng.806.
34. Anders Peterson, Axel Verdier, Ayman Abdel Ghany, Ben Bimber, Byunggil Yoo, Daniel Gómez-Sánchez, et al. broadinstitute/gatk - scripts/mitochondria_m2_wdl, 2021. `https://github.com/broadinstitute/gatk/tree/`

DRAFT

33bda5e08b6a09b40a729ee525d2e3083e0ecdf8/scripts/mitochondria_m2_wdl.
35. Yuichi Kodama, Jun Mashima, Takehide Kosuge, Toshiaki Katayama, Takatomo Fujisawa, Eli Kaminuma, et al. The DDBJ Japanese Genotype-phenotype Archive for genetic and phenotypic human data. *Nucleic Acids Research*, 43(D1):D18–D22, 2015. doi: 10.1093/nar/gku1120.
36. National Bioscience Database Center. Whole genome sequencing analysis - NBDC Human Database, 2023. https://humandbs.biosciencedbc.jp/en/whole-genome-sequencing.
37. Bioinformation and DDBJ Center. ddbj/jga-analysis, 2023. https://github.com/ddbj/jga-analysis.
38. Hirotaka Suetake. sapporo-wes/tonkaz - tests/README.md, 2022. https://github.com/sapporo-wes/tonkaz/blob/main/tests/README.md. doi: 10.5281/zenodo.7102376.
39. Hirotaka Suetake. Raw data of workflow execution results used in Tonkaz's experiments, 2023. doi: 10.5281/zenodo.7660388.
40. Aaron McKenna, Matthew Hanna, Eric Banks, Andrey Sivachenko, Kristian Cibulskis, Andrew Kernytsky, et al. The Genome Analysis Toolkit: A MapReduce framework for analyzing next-generation DNA sequencing data. *Genome Research*, 20(9):1297–1303, 2010. doi: 10.1101/gr.107524.110.
41. Alexander Dobin, Carrie A. Davis, Felix Schlesinger, Jorg Drenkow, Chris Zaleski, Sonali Jha, et al. STAR: Ultrafast universal RNA-seq aligner. *Bioinformatics*, 29(1):15–21, 2013. doi: 10.1093/bioinformatics/bts635.
42. Mihaela Pertea, Geo M. Pertea, Corina M. Antonescu, Tsung-Cheng Chang, Joshua T. Mendell, and Steven L. Salzberg. StringTie enables improved reconstruction of a transcriptome from RNA-seq reads. *Nature Biotechnology*, 33(3):290–295, 2015. doi: 10.1038/nbt.3122.
43. nf-core Community. nf-core/test-datasets - rnaseq/samplesheet/v3.4/samplesheet_test.csv, 2018. https://raw.githubusercontent.com/nf-core/test-datasets/rnaseq/samplesheet/v3.4/samplesheet_test.csv.
44. Hirotaka Suetake and Tazro Ohta. sapporo-wes/test-workflow: 1.0.2 - assets/nf-core_rnaseq_samplesheet_small_test.csv, 2022. https://raw.githubusercontent.com/sapporo-wes/test-workflow/1.0.2/assets/nf-core_rnaseq_samplesheet_small_test.csv. doi: 10.5281/zenodo.7102664.
45. Daniel Nüst and Stephen J. Eglen. CODECHECK: An Open Science initiative for the independent execution of computations underlying research articles during peer review to improve reproducibility. *F1000Research*, 10:253, 2021. doi: 10.12688/f1000research.51738.1.
46. Philip Ewels, Måns Magnusson, Sverker Lundin, and Max Käller. MultiQC: Summarize analysis results for multiple tools and samples in a single report. *Bioinformatics*, 32(19): 3047–3048, 2016. doi: 10.1093/bioinformatics/btw354.
47. Workflow Run RO-Crate working group. Workflow Run RO-Crate, 2023. https://www.researchobject.org/workflow-run-crate/.
48. Hirotaka Suetake and Tazro Ohta. sapporo-wes/test-workflow: 1.0.2, 2022. doi: 10.5281/zenodo.7102664.

DRAFT
