# Supplementary material for: A workflow reproducibility scale for automatic validation of biological interpretation results
Source: Gigascience. 2023 May 8;12:giad031. doi: 10.1093/gigascience/giad031 (PMC10164546; doi:10.1093/gigascience/giad031)
Supplement: giad031_Response_to_Reviewer_Comments_Original_Submission [file giad031_response_to_reviewer_comments_original_submission.pdf]

Author's Response To Reviewer Comments

Close

GIGA-D-22-00300
A workflow reproducibility scale for automatic validation of biological interpretation results
Hirotaka Suetake; Tsukasa Fukusato; Takeo Igarashi; Tazro Ohta
GigaScience

We would like to thank the reviewers for their positive and constructive feedback. The revised manuscript highlighted the changes in red color. Our responses to the issues pointed out by each reviewer are as follows.

Reviewer #1

> Make explicit why these 3 workflows where selected (see Q2)

> (From Q2:) It is not explicit in the text why these particular workflows were selected, beyond being realistic pipelines used in research. I would suggest something like "these workflows have been selected as fairly representative and mature current best-practice for sequencing pipelines, implemented in different but typical workflow systems, and have similar set of genomics features that we can assess for provenance comparison.

We are appreciated for the suggestion. We added the suggested sentence in the first paragraph of the Result section.

> Make pipeline software citations consistent in manuscript (see Q2, Q5)

We fixed the citation with the appropriate publications and the URL for the software.

> Avoid declaring CC0 within generated RO-Crate -- move this to only apply to the ro-crate-metadata.json

We fixed the indicated license issue in our Zenodo repository by updating the contents. We also updated the DOI in the manuscript. We added the following paragraph to the Discussion section to explain the license issue related to an auto-generated RO-crate:

When generating workflow provenance using a format, such as RO-Crate, it is important to consider licensing issues. The provenance includes not only the execution results, but also the executed workflow, input datasets, and software used internally. These files and software may have different licenses, and combining them under a single license can cause relicensing problems. In RO-Crate, a license can be specified for each entity; however, this approach is not currently possible as Sapporo automatically generates provenance from run requests and execution results without the original license information. This limitation can be overcome if data and software are consistently able to present their licenses, but this would require a generic method to get the license information of files retrieved from the internet.

> Add an outer RO-Crate metadata file to Zenodo deposit to carry the correct licenses and pipeline licenses for each of rnaseq_1st.zip, trimming.zip, etc.

We added the License.txt and ro-crate-metadata.json files to declare the licenses in the zip archived files.

> Improve discussion to better reflect limitations of the features and its own reproducibility issues (see Q7, Q9)

We added the following paragraph to the Discussion section:

Though Tonkaz aims to improve the reproducibility of data analysis, the system itself also has a challenge in the reproducibility of its function. The system uses the file extension to check the file type, then specifies an external tool to extract the biological features from the file to compare the workflow outputs. However, the extracted features may change by the updates in the external tools, which results in the inconsistency of the results of comparison by Tonkaz. Another issue we see in the reproducibility of the comparison is the system's dependency on Sapporo, our WES implementation. Ideally, the results, analysis summaries, logs, etc. generated by analysis tools should be in a standardized format so any system can generate comparable statistics. The bioinformatics community needs to have a consensus for such outputs of data analysis. As a related project, MultiQC attempts to summarize the results of multiple analysis tools~\cite{ewels_multiqc_2016}. The Tonkaz system may improve its future consistency by integrating with a community effort like MultiQC, which can share the effort to extract the information from the analysis tools.

> Consider improvements to the RO-Crate context (see Q10) - this may just be noted as Future Work in the manuscript rather than regenerating the crates

We added the following paragraph to the Discussion section:

We used RO-Crate to express the provenance of our study and added additional terms and properties to the "@context" declaration for verification purposes. These terms are currently located on our own GitHub repository, but we are discussing with the RO-Crate community moving them to a more authoritative location, such as https://github.com/ResearchObject/ro-terms. In future work, we are also considering using the Workflow Run RO-Crate profile, which is currently under development to capture the provenance of executing a computational workflow, instead of our custom terms.

> p2: Add citation for claim on file checksums different depending on software versions etc., for instance,
> p3. "We converted Sapporo's provenance into RO-Crate" -- re-cite (20) as this is the paragraph explaining what it is.
> p10. Citations 7, 8 are missing authors
> p10. Citation 15 is now published, replace with https://doi.org/10.1145/3486897
> p0. Citations 28, 33 is missing DOI

We updated the manuscript following the reviewer's suggestion. Citations 7 and 8 miss the author information because they are the editorials without authors also in their specified citation form. We thank the reviewer for checking in detail.

Reviewer #2

> The manuscript indicates that it's not feasible to compare images automatically. However, this is pretty easy. For example, using the Pillow package in Python, you can calculate a percentage similarity between two image files. I'm not suggesting that the authors should do this in their study. But the text should not preclude this as a possibility.

We agree with the reviewer's suggestion. We updated the second paragraph of the "Automatic verification of reproducibility" section as follows:

Among the various types of output files, including analysis results, summary reports, or execution logs, the system needs to select the files to compare. We aimed to compare the analysis results that led to a biological interpretation and to avoid the comparison of the output files that are not in a standard format. Therefore, we selected the file types to be compared as an initial set and selected the corresponding EDAM ontology terms listed in Table~\ref{tab:edamFileTypes}. With this selection, for example, the nf-core RNA-seq workflow produces 872 files, but only 25 files are assigned to the EDAM ontology and compared.

> The authors describe scenarios where the outputs might be different but these differences would be

immaterial to the overall conclusions. They also describe a few scenarios where the outputs differ for biological features but the differences are relatively small and could be considered to be acceptable. Examples include when BAM files are sorted differently. I think it would be helpful to add a bit more discussion of scenarios where differences in biological features could occur and what would cause those differences.

We added the following paragraph in the Discussion section:

In the Result section, we showed the cases where the differences are found in the outputs but the biological interpretation will be identical. However, there are cases where users find differences that affect the interpretation even when comparing the same workflow definitions. For example, the output results may change when the workflow has a tool that dynamically uses external databases, which may be regularly updated over time. Another case when the impact on the results can be observed is a comparison of runs of the workflow which does not explicitly specify the software version nor properly packaged.

> Although a person checking the outputs can change the numeric threshold, it would be difficult to know what that threshold should be. Perhaps the authors could describe the additional situation(s) where having relatively large differences would be acceptable and other situation(s) where they would not. For example, you could have a single difference in the biological feature outputs and perhaps that would make a huge difference in the interpretation in some cases. Additional discussion would be helpful.

We added the following paragraph to the Discussion section:

As the system allows users to change the reporting threshold in comparison to the outputs, users need to be aware of the acceptable differences in the outputs of the given workflow. Although the threshold needs to be low for workflows used in applications that require severe quality control, such as medical data analysis, users can set it higher for workflows that can generate different outputs per run. For example, workflows using external databases, or used for environmental monitoring purposes, may have outputs that vary per run. The system alerts when a change was found, however, as the interpretation depends on the cases, users need to understand the reason from the workflow description.

> This paper focuses on automating the verification process. I think the big picture could be explained more. Who might perform this verification process in a scientific context? In what context would they do it?

We added the following paragraph to the Discussion section:

In a scientific context, automated verification is a crucial process that should be performed for various reasons. Workflow developers can use it to easily add or update code and improve development efficiency. Administrators of workflow registries can use it to perform quality control, such as checking for broken links between the analysis tools and data used internally. Users of the workflow can also use it to validate the behavior of the workflow as an acceptance test in their own environment, thereby improving the reliability of their research projects. Tonkaz aims to support these validation efforts in different use cases and promote open science.

> Please add brief discussion about generalizing this methodology beyond Tonkaz.

We added the following paragraph to the Discussion section:

The proposed method is currently dependent on our software implementation; however, it can be generalized by the following three steps: A) Extract the statistics of biological features from the output, B) Represent the statistics in a standardized format, C) Compare the statistics, and report in the reproducibility scale. Although we implemented A and B in Sapporo, it is ideal to let workflow execution systems have those two steps rather than a WES implementation. Once steps A and B became common, step C can be implemented in many kinds of data analysis platforms, while Tonkaz only provides a CLI interface. However, the bioinformatics community needs to have a consensus on the standardized scale for reproducibility.

Again, we thank the reviewers for their comments and suggestions that improved the manuscript.

Close
