# Supplementary material for: A workflow reproducibility scale for automatic validation of biological interpretation results
Source: Gigascience. 2023 May 8;12:giad031. doi: 10.1093/gigascience/giad031 (PMC10164546; doi:10.1093/gigascience/giad031)
Supplement: giad031_Reviewer_1_Report_Original_Submission — Stian Soiland-Reyes -- 12/14/2022 Reviewed [file giad031_reviewer_1_report_original_submission.pdf]

Reviewer Report

Title: A workflow reproducibility scale for automatic validation of biological interpretation results

Version: Original Submission **Date:** 12/14/2022

Reviewer name: Stian Soiland-Reyes

Reviewer Comments to Author:

Hi, I am Stian Soiland-Reyes https://orcid.org/0000-0001-9842-9718 and have pledged the Open Peer Review Oath <https://doi.org/10.12688/f1000research.5686.2>:
* Principle 1: I will sign my name to my review
* Principle 2: I will review with integrity
* Principle 3: I will treat the review as a discourse with you; in particular, I will provide constructive criticism
* Principle 4: I will be an ambassador for the practice of open science
This review is licensed under a Creative Commons Attribution 4.0 International License
http://creativecommons.org/licenses/by/4.0/
and is also available at the (For now) *secret* URL
https://gist.github.com/stain/cddf0b309017f3e817d0f5b486947b04
which may better represent the formatting of this review. (See also attached HTML).

This article presents a method for comparing reproducibility of computational workflow runs captured as RO-Crates, by calculating a set of genomics metrics ("features") and adding these to the crate's metadata. Overall I find this a valuable contribution and worthy of publication with GigaScience, primarily as a way for users of workflow systems CWL, Nextflow, Cromwell or Snakemake to ensure reproducibility, but also for workflow engine developers who may want to build on this methodology to improve their provenance support.
In general the method proposed is sound, however it does have some limitations and inherent assumptions that are not highlighted sufficiently in the current manuscript, particularly concerning the selection of features and the reproducibility of the metrics calculation itself. I have detailed this with some points below that I would like the authors to clarify in a minor revision.

Note - the below questions from GigaScience Reviewer Guidelines mainly relate to _data_, but I also here interpret them for the _software_ described.
Q1: Is the rationale for collecting and analyzing the data well defined?
The author's workflow executions <https://doi.org/10.5281/zenodo.7098337> are based on three 3rd-party bioinformatics workflows. Although they are not particularly "large-scale", they are representative best-practice pipelines in this field (data sizes from 200 MB to 6 GB) and also fairly representative for scalable workflow systems (Nextflow, CWL and WDL) used by bioinformaticians.
Q2: Is it clear how data was collected and curated?
It is not explicit in the text why these particular workflows were selected, beyond being realistic

pipelines used in research. I would suggest something like "these workflows have been selected as fairly representative and mature current best-practice for sequencing pipelines, implemented in different but typical workflow systems, and have similar set of genomics features that we can assess for provenance comparison."

The workflows have each been cited, but I would appreciate some consistency so that each workflow is cited both by its closest journal article **and** as their original download sources (e.g. GitHub).

Q3: Is it clear - and was a statement provided - on how data and analyses tools used in the study can be accessed?

Yes, full availability statements have been provided both for data and software, archived on Zenodo for longevity.

Q4: Are accession numbers given or links provided for data that, as a standard, should be submitted to a community approved public repository?

Yes, the tools have been added to https://bio.tools/ -- I don't think it's necessary to further register the data outputs with accession numbers. RRIDs for tools can be considered at a later stage, perhaps only for Sapporo.

Q5: Is the data and software available in the public domain under a Creative Commons license?

Yes, the software and dataset is open source under Apache License, version 2.0.

The dataset <https://doi.org/10.5281/zenodo.7098337> embeds existing workflows and data, however this is OK as included resources such as the rnaseq Nextflow workflow have compatible licenses (MIT) or are also Apache-licensed.

The manuscript has software citations for two of the workflows, but this is missing for the CWL workflow, which is only cited by manuscript (33) (also missing DOI). It is unclear if any of the workflows are registered in <https://workflowhub.eu/> but that should primarily be done by their upstream authors.

The RO-Crates in <https://doi.org/10.5281/zenodo.7098337> don't include any licensing and attribution for the embedded workflows, and its metadata file is misleadingly declaring the crate license as CC0 public domain. While CC0 is appropriate for examples and metadata file itself, the embedded MIT/Apache workflows from third parties can't legally be relicensed in this way and should have their original licenses declared. See <https://www.researchobject.org/ro-crate/1.1/contextual-entities.html#licensing-access-control-and-copyright>

I understand these RO-Crates are generated automatically by Sapporo, which does not directly understand licensing, and for documenting the test runs with Sapporo, I think these should not be modified post-execution. Pending further license support by Sapporo, perhaps a manual outer RO-Crate that aggregate these (e.g. adding a direct top-level ro-crate-metadata.json to the Zenodo entry) can provide more correct metadata as well as workflow citations.

The authors could add to Discussion some consideration on (lack of) propagation of such metadata for auto-generated crates as part of workflow run provenance. For instance, if a workflow run was initiated from a Workflow Crate <https://w3id.org/workflowhub/workflow-ro-crate/> at WorkflowHub, its license, attributions and descriptions could be carried forward to the final Workflow Run Crate provenance together with the Sapporo-calculated features.

Q6: Are the data sound and well controlled?

Yes, the data is sound. The testing on Mac gives null-results, but the authors explain the workflows

failed to execute there due to archicectural differences, which is flagged as a valid concern for reproducibility. It may be worth further investigating if this is due to misconfiguration on that particular test machine in which case these columns should be removed.

Q7: Is the interpretation (Analysis and Discussion) well balanced and supported by the data?

The authors' discussion have some implicit assumptions that should be made more clear, together with implications:

1) The Tonkaz tool assumes the workflow execution has already extracted the features and added them to the RO-Crate
2) This assumes the right features have been correctly extracted by each execution
3) Feature extraction also depend on bioinformatics tools that are subject to change/updates
4) Newer versions of Sapporo-service, and in particular any non-Sapporo executors also making Workflow run Crates, may have a different feature selection
5) Being able to fairly compare two workflow runs therefore depends on careful control of the Sapporo executor versions so that they have consistent feature selection
6) This means the reproducibility metrics proposed has a potential reproducibility challenge itself

This is not to say that the approach is bad, as the feature extraction is using predictable measures such as counting sequences, rather than heuristics. This means Future Work should point out the need for guidelines on what kind of features should be selected, to ensure they are consistent and reproducible. The set of features also depend on the type of data and class of analysis.

As a minimum, the RO-Crate should therefore include provenance of that feature extraction, noting the Sapporo version, and ideally the version of the tools used for that.

The authors may want to consider if feature extraction should be a separate workflow (e.g. in CWL), that itself can be subject to the same reproducibility preservation measures, and therefore also can be performed post-execution as part of Tonkaz' comparison or as a curation activity when storing Workflow Run Crates.

Q8: Are the methods appropriate, well described, and include sufficient details and supporting information to allow others to evaluate and replicate the work?

Yes, it was very easy to replicate the Tonkaz analysis of the workflow run crate that is already provided, as it is provided also as a Docker container. The Docker container is provided as part of GitHub releases, and so is not at risk of Docker Hub's automatic deletion.

I have not tried installing my own Sapporo service to re-execute the workflow, but detailed installation and run details are provided in the README of both Tonkaz <https://github.com/sapporo-wes/tonkaz#readme> and sapporo-service <https://github.com/sapporo-wes/sapporo/blob/main/docs/GettingStarted.md>

Q9: What are the strengths and weaknesses of the methods?

The method provided is strong compared to naive checksum-based comparison of workflow outputs, which has been pointed out as a challenge by previous work. The advantage of the feature extraction is that the statistics can be compared directly and any disreprancies can be displayed to the user at a digestible high-level.

The disadvantage is that this depends wholy on the selection of features, which must be done carefully to cover the purpose of the particular workflow and its type of data. For instance, a workflow that generates diagrams of sequence alignments could not be sufficiently tested in the suggested approach,

as analyzing the diagram for correctness would require tools that may not even exist. Perhaps feature extraction should be a part of the workflow itself, so it can self-determine what is important for its analysis?
The current approach also is quite sensitive to output data filenames, so changes in filename would mean features are not compared, even where such files are equivalent. This should be made more explicit in the manuscript, for instance workflows should ensure they don't include timestamps or random identifiers in their filenames. Further work could have a deeper understanding of the workflow structure to compare outputs based on their corresponding FormalParameter in the RO-Crate.

Q10: Have the authors followed best-practices in reporting standards?

Yes, the details provided are at a sufficient detail level, and the authors have re-used the RO-Crate data packaging.
The RO-Crates created by Sapporo-service adds several terms for the metrics, which are declared on the `@context` according to RO-Crate specs <https://www.researchobject.org/ro-crate/1.1/appendix/jsonld.html#extending-ro-crate>
However the terms point to GitHub "raw" pages, which are not particularly stable, and may change depending on sapporo versions and GitHub's repository behaviour.
I recommend changing the ad-hoc terms to PIDs such as a namespace under https://w3id.org/ or https://purl.org/ so that these terms can be stable semantic artefacts, e.g. submitting them to <https://github.com/ResearchObject/ro-terms> to register <https://w3id.org/ro/terms/sapporo#WorkflowAttachment> that can be used instead of <https://raw.githubusercontent.com/sapporo-wes/sapporo-service/main/sapporo/ro-terms.csv#WorkflowAttachment> or alternatively https://w3id.org/sapporo#WorkflowAttachment could be set up to redirect to the ro-terms.csv on GitHub. (discussed with the authors at ELIXIR Biohackathon)
In doing so you should separate into two namespaces, the general Sapporo terms like "sha512", and the particular genomics feature sets including "totalReads" (e.g. <https://w3id.org/data-features/genomics#WorkflowAttachment>) as the second are a) Not sapporo-specific b) domain-specific.
RO-Crate is developing Workflow Run profiles <https://www.researchobject.org/workflow-run-crate/profiles/>, although these have not been released at time of my review they are now stable, so the authors may want to check <https://www.researchobject.org/workflow-run-crate/profiles/workflow_run_crate> to ensure "FormalParameter" are declared correctly in the generated RO-Crate as separate entities, linked from the "File" using "exampleOfWork".

Q11: Can the writing, organization, tables and figures be improved?

The language and readability of this article is generally very good. Light copy-editing may improve some of the sentences, e.g. reducing the use of "Thus" phrases.

Q12: When revisions are requested.

See suggestions from above for minor revisions:
* Make explicit why these 3 workflows where selected (see Q2)
* Make pipeline software citations consistent in manuscript (see Q2, Q5)
* Avoid declaring CC0 within generated RO-Crate -- move this to only apply to the ro-crate-metadata.json

* Add an outer RO-Crate metadata file to Zenodo deposit to carry the correct licenses and pipeline licenses for each of rnaseq_1st.zip, trimming.zip etc.
* Improve discussion to better reflect limitations of the features and its own reproducibility issues (see Q7, Q9)
* Consider improvements to the RO-Crate context (see Q10) - this may just be noted as Future Work in the manuscript rather than regenerating the crates

In addition:

p2: Add citation for claim on file checksums different depending on software versions etc., for instance <https://doi.org/10.1145/3186266>

p3. "We converted Sapporo's provenance into RO-Crate" -- re-cite (20) as this is the paragraph explaining what it is.

p10. Citations 7, 8 are missing authors

p10. Citation 15 is now published, replace with https://doi.org/10.1145/3486897

p0. Citations 28, 33 is missing DOI

Q13: Are there any ethical or competing interests issues you would like to raise?

No, the third-party pipelines selected for reproducibility testing are already published and are here represented fairly, and only used as executable methods (as intended by their original authors), which I would say do not need ethical approval.

Methods

Are the methods appropriate to the aims of the study, are they well described, and are necessary controls included? Choose an item.

Conclusions

Are the conclusions adequately supported by the data shown? Choose an item.

Reporting Standards

Does the manuscript adhere to the journal's guidelines on minimum standards of reporting? Choose an item.

Choose an item.

Statistics

Are you able to assess all statistics in the manuscript, including the appropriateness of statistical tests used? Choose an item.

Quality of Written English

Please indicate the quality of language in the manuscript: Choose an item.

Declaration of Competing Interests

Please complete a declaration of competing interests, considering the following questions:

- Have you in the past five years received reimbursements, fees, funding, or salary from an organisation that may in any way gain or lose financially from the publication of this manuscript, either now or in the future?
- Do you hold any stocks or shares in an organisation that may in any way gain or lose financially from the publication of this manuscript, either now or in the future?
- Do you hold or are you currently applying for any patents relating to the content of the manuscript?
- Have you received reimbursements, fees, funding, or salary from an organization that holds or has applied for patents relating to the content of the manuscript?
- Do you have any other financial competing interests?
- Do you have any non-financial competing interests in relation to this paper?

If you can answer no to all of the above, write 'I declare that I have no competing interests' below. If your reply is yes to any, please give details below.

I am co-lead of RO-Crate, used by the reviewed work. At the ELIXIR Biohackathon (Nov 2022) the authors asked for my advice on extending RO-Crate for Sapporo, however this submitted work predates that discussion. In this review I mention WorkflowHub, managed by The University of Manchester.

I agree to the open peer review policy of the journal. I understand that my name will be included on my report to the authors and, if the manuscript is accepted for publication, my named report including any attachments I upload will be posted on the website along with the authors' responses. I agree for my report to be made available under an Open Access Creative Commons CC-BY license (http://creativecommons.org/licenses/by/4.0/). I understand that any comments which I do not wish to be included in my named report can be included as confidential comments to the editors, which will not be published.

Choose an item.

To further support our reviewers, we have joined with Publons, where you can gain additional credit to further highlight your hard work (see: https://publons.com/journal/530/gigascience). On publication of this paper, your review will be automatically added to Publons, you can then choose whether or not to claim your Publons credit. I understand this statement.

Yes Choose an item.
