# Supplementary material for: A workflow reproducibility scale for automatic validation of biological interpretation results
Source: Gigascience. 2023 May 8;12:giad031. doi: 10.1093/gigascience/giad031 (PMC10164546; doi:10.1093/gigascience/giad031)
Supplement: giad031_Reviewer_2_Report_Original_Submission — Stephen R Piccolo, Ph.D. -- 12/16/2022 Reviewed [file giad031_reviewer_2_report_original_submission.pdf]

Reviewer Report

Title: A workflow reproducibility scale for automatic validation of biological interpretation results

Version: Original Submission **Date:** 12/16/2022

Reviewer name: Stephen R Piccolo, Ph.D.

Reviewer Comments to Author:

This manuscript describes a methodology for automating evaluation of the reproducibility of data-science workflows for genomics analyses. The authors explain that reproducibility should be evaluated on a scale rather than on a binary basis. They explain concepts related to these issues and apply their methodology to real-world data.

The manuscript was well written and addresses an important issue. I believe this manuscript provides new insights. I have a few minor concerns that I would appreciate being addressed:

- The manuscript indicates that it's not feasible to compare images automatically. However, this is actually pretty easy. For example, using the Pillow package in Python, you can calculate a percentage similarity between two image files. I'm not suggesting that the authors should do this in their study. But the text should not preclude this as a possibility.
- The authors describe scenarios where the outputs might be different but these differences would be immaterial to the overall conclusions. They also describe a few scenarios where the outputs differ for biological features but that the differences are relatively small and could be considered to be acceptable. Examples include when BAM files are sorted differently. I think it would be helpful to add a bit more discussion of scenarios where differences in biological features could occur and what would cause those differences.
- Although a person checking the outputs can change the numeric threshold, it would be difficult to know what that threshold should be. Perhaps the authors could describe additional situation(s) where having relatively large differences would be acceptable and other situation(s) where they would not. For example, you could have a single difference in the biological feature outputs and perhaps that would make a huge difference in the interpretation in some cases. Additional discussion would be helpful.
- This paper focuses on automating the verification process. I think the big picture could be explained more. Who might perform this verification process in a scientific context? In what context would they do it?
- Please add brief discussion about generalizing this methodology beyond Tonkaz.

Methods

Are the methods appropriate to the aims of the study, are they well described, and are necessary controls included? Choose an item.

Conclusions

Are the conclusions adequately supported by the data shown? Choose an item.

Reporting Standards

Does the manuscript adhere to the journal's guidelines on [minimum standards of reporting?] Choose an item.

Choose an item.

Statistics

Are you able to assess all statistics in the manuscript, including the appropriateness of statistical tests used? Choose an item.

Quality of Written English

Please indicate the quality of language in the manuscript: Choose an item.

Declaration of Competing Interests

Please complete a declaration of competing interests, considering the following questions:

- Have you in the past five years received reimbursements, fees, funding, or salary from an organisation that may in any way gain or lose financially from the publication of this manuscript, either now or in the future?
- Do you hold any stocks or shares in an organisation that may in any way gain or lose financially from the publication of this manuscript, either now or in the future?
- Do you hold or are you currently applying for any patents relating to the content of the manuscript?
- Have you received reimbursements, fees, funding, or salary from an organization that holds or has applied for patents relating to the content of the manuscript?
- Do you have any other financial competing interests?
- Do you have any non-financial competing interests in relation to this paper?

If you can answer no to all of the above, write 'I declare that I have no competing interests' below. If your reply is yes to any, please give details below.

I declare that I have no competing interests.

I agree to the open peer review policy of the journal. I understand that my name will be included on my report to the authors and, if the manuscript is accepted for publication, my named report including any attachments I upload will be posted on the website along with the authors' responses. I agree for my report to be made available under an Open Access Creative Commons CC-BY license (http://creativecommons.org/licenses/by/4.0/). I understand that any comments which I do not wish to be included in my named report can be included as confidential comments to the editors, which will not be published.

Choose an item.

To further support our reviewers, we have joined with Publons, where you can gain additional credit to further highlight your hard work (see: https://publons.com/journal/530/gigascience). On publication of this paper, your review will be automatically added to Publons, you can then choose whether or not to claim your Publons credit. I understand this statement.

Yes Choose an item.
